# Efficient synthesis of novel thiadiazolo[2,3-b]quinazolin-6-ones catalyzed by diphenhydramine hydrochloride-CoCl_2_⋅6H_2_O deep eutectic solvent

**DOI:** 10.1038/s41598-024-52017-3

**Published:** 2024-01-16

**Authors:** Mehri Moeini Korbekandi, Iraj Mohammadpoor-Baltork, Majid Moghadam, Shahram Tangestaninejad, Valiollah Mirkhani, Behrouz Notash

**Affiliations:** 1 Department of Chemistry, Catalysis Division, University of Isfahan, Isfahan, 81746-73441 Iran; 2 Department of Inorganic Chemistry, Shahid Beheshti University, Tehran, Iran

**Keywords:** Catalysis, Green chemistry, Organic chemistry, Chemical synthesis

## Abstract

In this research, a new Lewis acid-based deep eutectic solvent (LA-DES) was synthesized using diphenhydramine hydrochloride and CoCl_2_·6H_2_O, (2[HDPH]:CoCl_4_^2−^), and identified by FT-IR and ^1^HNMR techniques. The physicochemical properties of this LA-DES, such as thermal behavior, thermal stability, and solubility in common solvents were also investigated. The catalytic ability of 2[HDPH]:CoCl_4_^2−^ was ascertained in the efficient synthesis of a novel array of thiadiazolo[2,3-b]quinazolin-6-one scaffolds via a one-pot three-component reaction of dimedone/1,3-cyclohexanedione, aldehydes, and 5-aryl-1,3,4-thiadiazol-2-amines/3-(5-amino-1,3,4-thiadiazol-2-yl)-2*H*-chromen-2-one under solvent-free conditions. This catalyst was also successfully utilized for the synthesis of *mono*- and *bis*-thiadiazolo[2,3-b]quinazolin-6-ones from dialdehydes or *bis*-1,3,4-thiadiazol-2-amine. The simplicity of enforcement, short reaction time, avoidance of toxic organic solvents, scalability of the synthesis procedure, excellent atom economy, high reaction mass efficiency, and low E-factor are other outstanding advantages of this newly developed method. Furthermore, due to the convenient recovery and reuse of LA-DES, this protocol is economically justified and environmentally friendly.

## Introduction

Green chemistry and sustainability are two crucial concepts that have gained particular attention in chemical processes^1^. In this respect, designer solvents have been introduced and developed as excellent substitutes for conventional and toxic solvents. Deep eutectic solvents (DESs) are a new generation of designer solvents, generally prepared by mixing substances capable of forming hydrogen bonds^2^. The formation of hydrogen bonds will be associated with charge delocalization, which causes the melting point of the DES to be lower than any of the raw materials^3,4^. Compared to their previous generation, ionic liquids (ILs), DESs are more cost-efficient, relatively non-toxic, more biodegradable, and have a more straightforward preparation process^5–10^. Among the different types of DESs, Lewis acid-based (LA-DES) and bio-based ones have been extensively used in promoting organic transformations^11–16^. To prepare LA-DES, the first-row transition metals can be used as Lewis acid^13,17^. Cobalt is an attractive candidate for catalysis in chemical syntheses^18^. On the other hand, the synthesis of LA-DES from CoCl_2_·6H_2_O as one of the universally used salts of cobalt can improve its catalytic activity^19^. Moreover, the high efficiency, easy recovery, and reusability of this LA-DES make this non-corrosive liquefy catalyst superior to other homogeneous liquid acid catalysts^19^.

LA-DESs have been used as the solvent^20–24^ and/or catalyst^25–30^ in many multicomponent reactions for the synthesis of heterocyclic compounds which is of paramount interest due to their potential pharmaceutical and biological activities^31^. Particularly, fused thiadiazoloquinazolines are a valuable class of fused *N*- and *S*-containing heterocyclic compounds, showing outstanding anticancer^32^, antifungal^33^, antibacterial^34^, and anti-inflammatory^35^ activities. Up to now, due to the significance of fused thiadiazoloquinazolines, several methods have been developed for the synthesis of these heterocycles^36–43^ However, they suffer from certain drawbacks such as multistep reaction sequences, prolonged reaction times, low yield, use of costly starting materials, corrosive reagents, and toxic solvents, as well as non-reusable catalysts which led to serious environmental and safety problems. Thus, the development of a more environmentally friendly and efficient procedure using a green and recoverable catalyst for the preparation of these worthy heterocyclic compounds is still vitally required.

Due to the synthesis of complex and structurally diverse bioactive heterocyclic compounds, multicomponent reactions (MCRs) play a paramount role in organic and medicinal chemistry^44^. Moreover, high convergence, facile and simple performance, pot, atom, step, and cost economy (PASCE), bond-forming-index (BFI), and minimized waste generation are unique advantages of MCRs which make them preferable to the classical stepwise fashions^45,46^. Due to the observance of the principles of green chemistry, MCRs have gained a special place in organic synthesis^47^.

**Figure 1:**
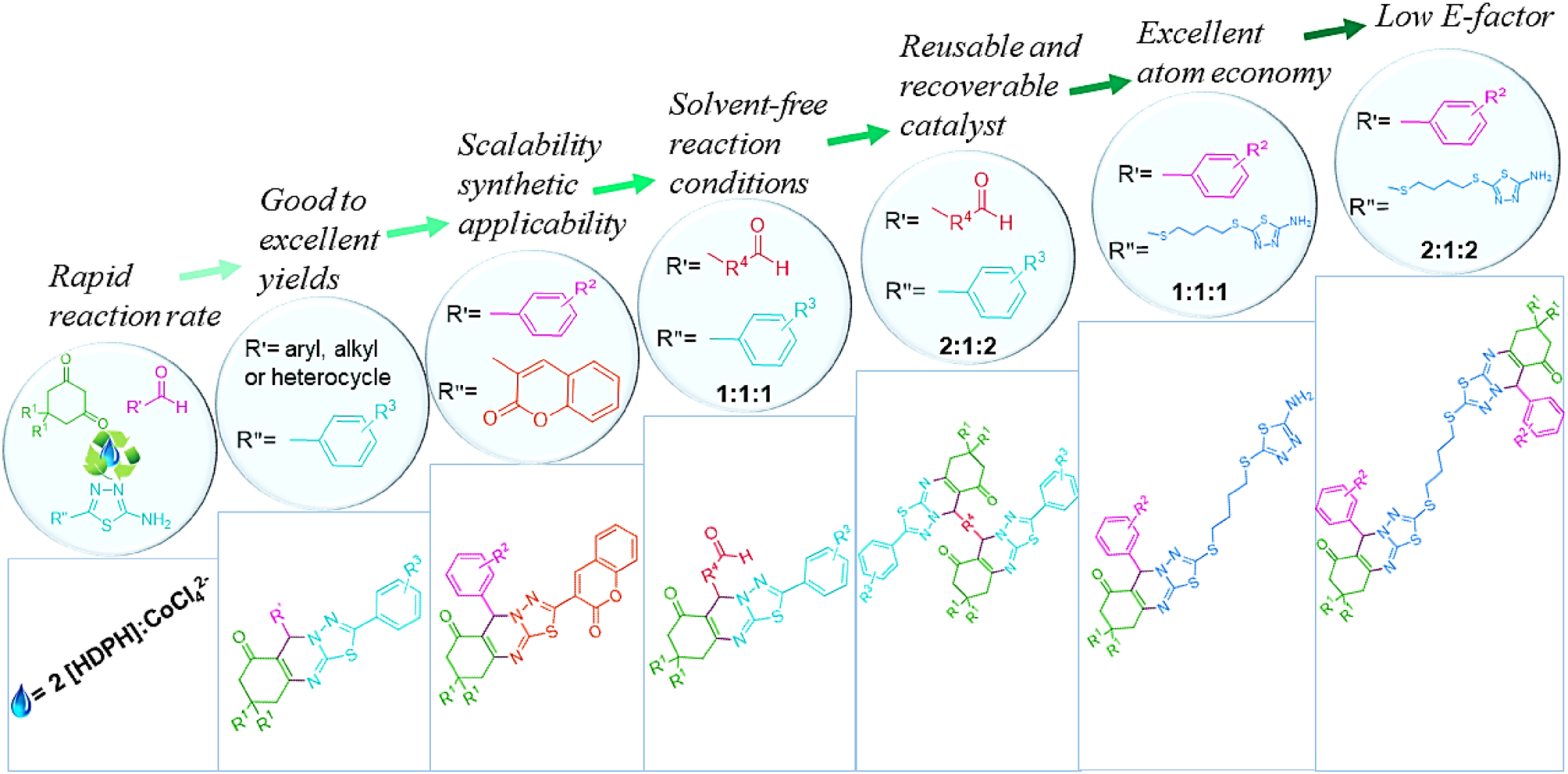
Synthesis of thiadiazolo[2,3-b]quinazolin-6-one catalyzed by 2[HDPH]:CoCl_4_^2*−*^.

As a part of our ongoing research on the synthesis of novel annulated heterocycles using green and reusable catalytic systems via multicomponent strategy^48–51^, we wish to describe an efficient and feasible method for the one-pot three-component synthesis of thiadiazolo[2,3-b]quinazolin-6-ones and also their *mono*- and *bis*-derivatives via the reaction of dimedone/1,3-cyclohexanedione, aldehydes/dialdehydes, and 5-substituted-1,3,4-thiadiazol-2-amines/*bis*-1,3,4-thiadiazol-2-amine using 2[HDPH]:CoCl_4_^2−^ as a novel and new LA-DES under solvent-free and green conditions (Fig. 1).

## Results and discussion

### Synthesis and characterization of 2[HDPH]:CoCl_4_^2−^

As mentioned in the experimental section, the LA-DES, (2[HDPH]:CoCl_4_^2−^) was easily formed by using a mixture of diphenhydramine hydrochloride and CoCl_2_·6H_2_O in a 2:1 molar ratio and identified by FT-IR, ^1^HNMR, DSC, and TGA/DTG techniques.

**Figure 2:**
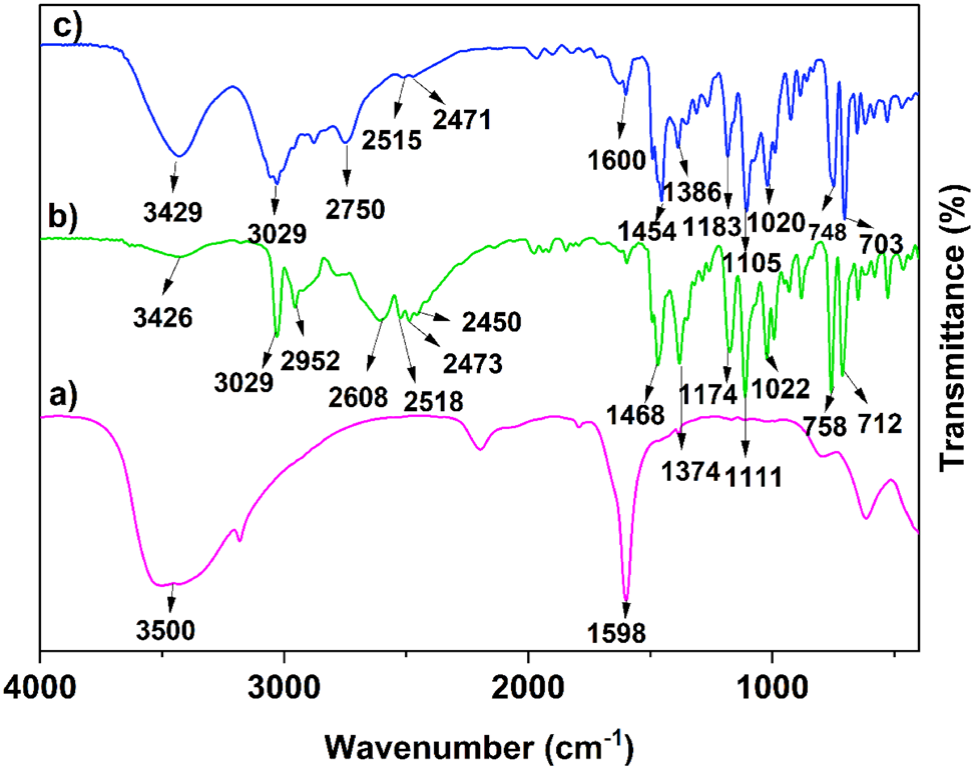
FT-IR spectra: (a) CoCl_2_⋅6H_2_O (magenta), (b) [HDPH]Cl (green), and (c) 2[HDPH]:CoCl_4_^2−^ (blue).

The functional groups of the desired LA-DES were recognized by FT-IR spectroscopy (Fig. 2). In the FT-IR spectrum of CoCl_2_·6H_2_O, the broad bands at 3500 and 1598 cm^−1^ are related to the vibrational modes of surface water^19^. In the spectra of [HDPH]Cl^50^ and [HDPH]:CoCl_4_^2−^, the specific bands were observed at 3029 cm^−1^ (sp^2^ C–H) and 1111 cm^−1^ (C–O–C). The characteristic bands at 1454 and 1386 cm^−1^ are related to CH_2_ and CH_3_ stretching vibrations, respectively. Remarkably, the bands at 2400–2700 cm^−1^ (–N^+^–H) are weakened in the LA-DES spectrum, which is good evidence for the formation of hydrogen bonding.

**Figure 3:**
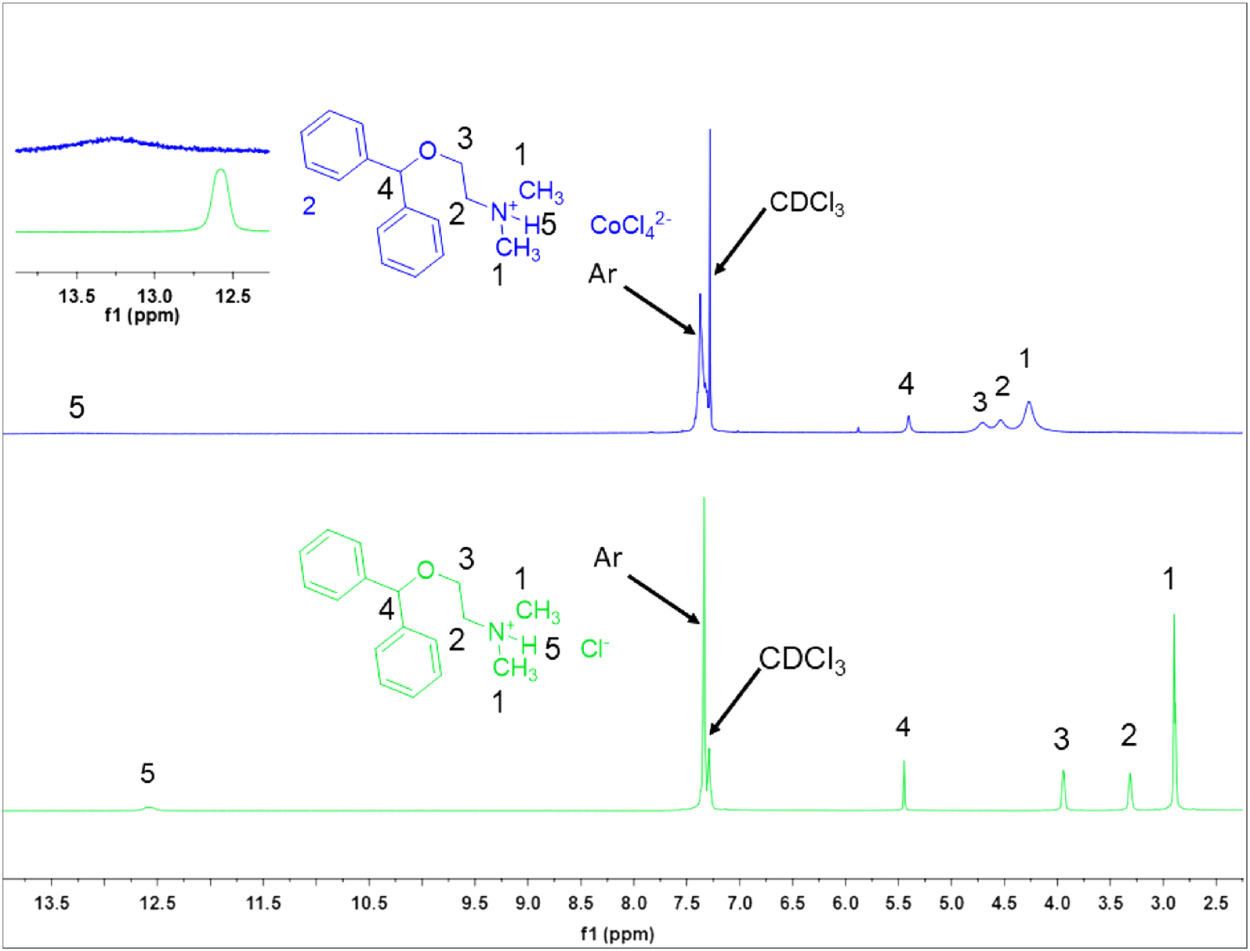
^1^HNMR spectra (400 MHz, CDCl_3_) of: (a) [HDPH]Cl (green) and (b) 2[HDPH]:CoCl_4_^2−^ (blue).

In the ^1^HNMR spectrum of 2[HDPH]:CoCl_4_^2−^, the aromatic hydrogens appeared as multiplet in the range of 7.42–7.31 ppm. The peaks at 4.70, 4.54, and 4.27 ppm are related to H^3^, H^2^, and H^1^_,_ respectively. Surprisingly, All the mentioned peaks are down-fielded compared to the similar peaks in the [HDPH]Cl spectrum^50^. Also, the characteristic peak corresponding to –N^+^–H in 2[HDPH]:CoCl_4_^2−^ shifted from 12.58 to 13.21 ppm and becomes wider (Fig. 3). These observations indicate significant hydrogen bond formation in 2[HDPH]:CoCl_4_^2−^.

**Figure 4:**
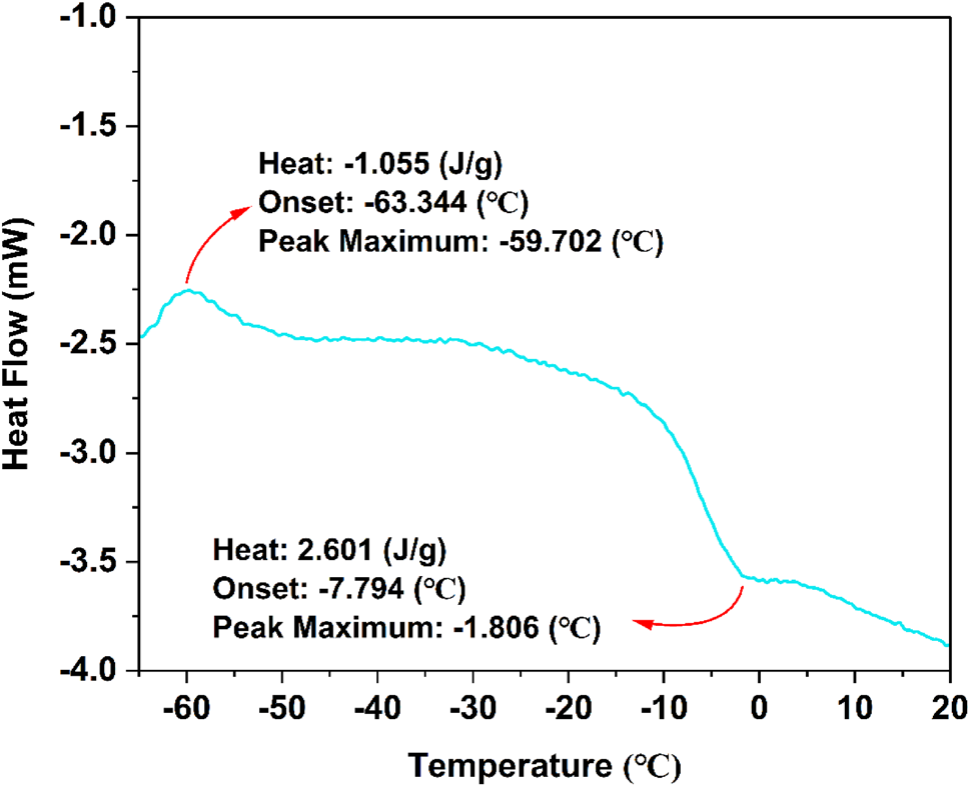
DSC curve of 2[HDPH]:CoCl_4_^2−^.

Differential scanning calorimetry (DSC) was effectively used to diagnose the thermal behavior of 2[HDPH]:CoCl_4_^2−^. The melting point of 2[HDPH]:CoCl_4_^2−^ in the DSC curve was observed at around − 2 °C (Fig. 4), which is lower than those of the raw materials (the melting points of [HDPH]Cl and CoCl_2_⋅6H_2_O are 169 °C, and 56 °C, respectively). Such a depression of melting point can be related to the formation of hydrogen bonds in 2[HDPH]:CoCl_4_^2−^ deep eutectic mixture.

**Figure 5:**
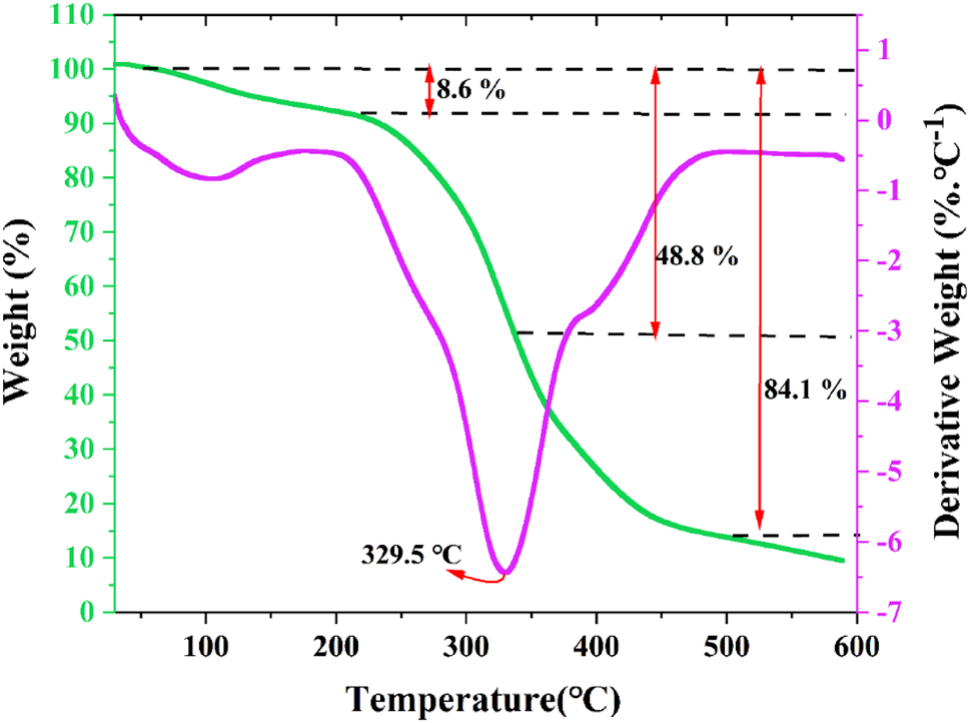
Thermogravimetric analysis and derivative thermogravimetry (TGA/DTG) of 2[HDPH]:CoCl_4_^2−^.

To examine the thermal stability of the 2[HDPH]:CoCl_4_^2−^, TGA analysis was employed. According to Fig. 5, the new LA-DES has good thermal stability up to about 329 °C; weight losses of only 5% at 136.1 °C and 10% at 232.9 °C were observed. The residual weight at 500 ℃ is 15.90%, which matches well with the theoretical CoCl_2_ weight % (15.80%).

**Figure 6:**
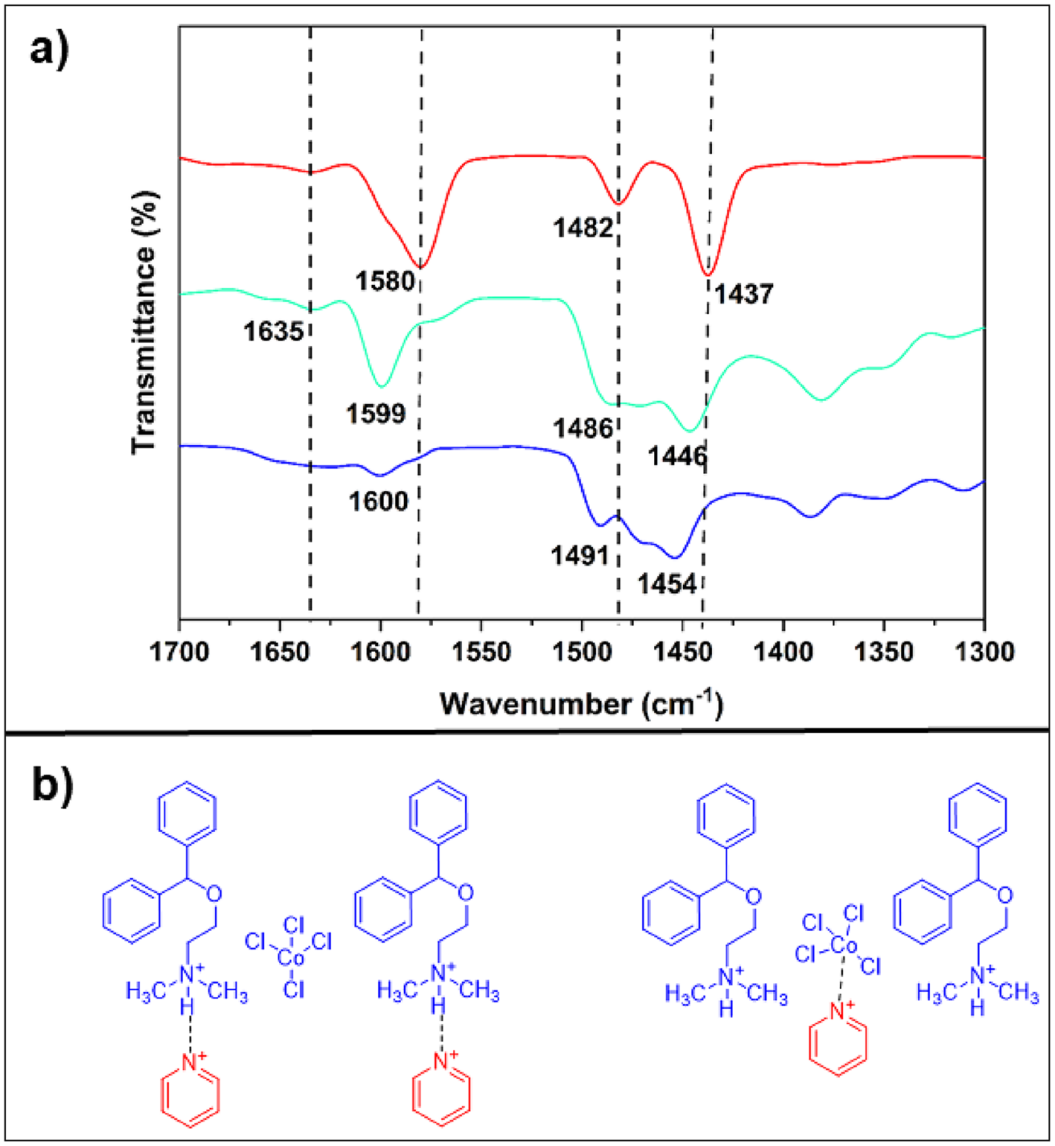
FT-IR spectra: (**a**) Pyridine (red), 2[HDPH]:CoCl_4_^2−^ (blue), and 2[HDPH]:CoCl_4_^2−^ in pyridine (green). (**b**) The interaction between the Lewis and Brønsted acid sites of the catalyst with pyridine.

Acidity is one of the most important physical properties of DESs, making them applicable for industrial usage. To determine the acidity of DES, FT-IR spectroscopy and pyridine as a probe are commonly utilized^28^. For this purpose, the FT-IR spectra of pyridine, 2[HDPH]:CoCl_4_^2−^ and pyridine-2[HDPH]:CoCl_4_^2−^ were investigated^52^. In the FT-IR spectrum of pyridine-2[HDPH]:CoCl_4_^2−^ (Fig. 6a), the characteristic band at 1446 cm^−1^, indicates that 2[HDPH]:CoCl_4_^2−^ has Lewis acidic sites which interact with pyridine groups. Also, the band appeared at 1635 cm^−1^ in the FT-IR spectrum of pyridine-2[HDPH]:CoCl_4_^2−^ could be attributed to the formation of pyridinium ions ([PyH]^+^) upon the interaction of pyridine with Brønsted acid sites of the LA-DES^3,50^. Such an interaction has been displayed in Fig. 6b. To confirm the acidity, pH of the solutions of CoCl_2_⋅6H_2_O (4 × 10^−3^ mol L^−1^), [HDPH]Cl (8 × 10^–3^ mol L^−1^), and 2[HDPH]:CoCl_4_^2−^ (8 × 10^–3^ mol L^−1^) were measured and found to be 5.17, 5.39, and 4.77, respectively. Consequently, 2[HDPH]:CoCl_4_^2−^ can be applied as an efficient acidic catalyst for useful organic transformations.

### Optimization of the reaction conditions

For the initial investigation, the reaction between 4-methylbenzaldehyde, 5-phenyl-1,3,4-thiadiazol-2-amine, and dimedone was selected as a model to optimize the reaction conditions. In the absence of the catalyst, the desired three-component product **4a** was obtained with only 5% yield even after 4 h at 120 ℃ under solvent-free conditions (Table 1, entry 1). Then, the titled reaction was performed in the presence of 0.2 mmol of catalysts including [HDPH]Cl, CoCl_2_·6H_2_O, and different LA-DESs at 120 ℃ under solvent-free conditions (Table 1, entries 2–8). Among these, 2[HDPH]:CoCl_4_^2−^ provided the highest yield (90%) of the desired product **4a** (Table 1, entry 8). Different molar ratios of [HDPH]Cl to CoCl_2_·6H_2_O were examined (Table 1, entries 8–10); 2:1 molar ratio was found to be more suitable for this transformation (Table 1, entry 8). It is noteworthy that increasing the loading of 2[HDPH]:CoCl_4_^2−^ to 0.3 mmol did not improve the product yield, but decreasing the loading of the catalyst to 0.1 mmol led to a lower yield of the product (Table 1, entries 11, 12). In addition, the reaction was also tested at various temperatures (Table 1, entries 8, 13–15), and 120 ℃ was discovered to be more suitable for this transformation. Consequently, the optimum reaction conditions for the synthesis of target product **4a** were attained by using 0.2 mmol of 2[HDPH]:CoCl_4_^2−^ at 120 ℃ under solvent-free conditions (Table 1, entry 8).

**Table 1:** Optimization of the reaction conditions for the model reaction**.**

Entry^a^	Catalyst (mmol)	Molar ratio	Abbreviation	Temperature (℃)	Yield (%)^b^
1	–	–	–	120	5
2	[HDPH]Cl (0.2)	–	–	120	12
3	CoCl_2_⋅6H_2_O (0.2)	–	–	120	31
4	[HDPH]Cl:CuCl_2_⋅2H_2_O (0.2)	2:1	2[HDPH]:CuCl_4_^2−^	120	73
5	[HDPH]Cl:MnCl_2_⋅2H_2_O (0.2)	2:1	2[HDPH]:MnCl_4_^2−^	120	50
6	[ChCl]:CuCl_2_⋅2H_2_O (0.2)	2:1	2[Ch]:CuCl_4_^2−^	120	51
7	[ChCl]:CoCl_2_⋅6H_2_O (0.2)	4:1	2[ChCl]0.2[Ch]:CoCl_4_^2−^	120	69
8	[HDPH]Cl:CoCl_2_⋅6H_2_O (0.2)	2:1	2[HDPH]:CoCl_4_^2−^	120	90
9	[HDPH]Cl:CoCl_2_⋅6H_2_O (0.2)	3:1	[HDPH]Cl.2[HDPH]: CoCl_4_^2−^	120	85
10	[HDPH]Cl:CoCl_2_⋅6H_2_O (0.2)	4:1	2[HDPH]Cl.2[HDPH]: CoCl_4_^2−^	120	87
11	[HDPH]Cl:CoCl_2_⋅6H_2_O (0.1)	2:1	2[HDPH]:CoCl_4_^2−^	120	68
12	[HDPH]Cl:CoCl_2_·6H_2_O (0.3)	2:1	2[HDPH]:CoCl_4_^2−^	120	90
13	[HDPH]Cl:CoCl_2_·6H_2_O (0.2)	2:1	2[HDPH]:CoCl_4_^2−^	130	90
14	[HDPH]Cl:CoCl_2_⋅6H_2_O (0.2)	2:1	2[HDPH]:CoCl_4_^2−^	100	43
15	[HDPH]Cl:CoCl_2_⋅6H_2_O (0.2)	2:1	2[HDPH]:CoCl_4_^2−^	80	20

^a^Reaction conditions: dimedone **1a** (1 mmol), 4-methyl benzaldehyde **2a** (1 mmol), and 5-phenyl-1,3,4-thiadiazol-2-amine **3a** (1 mmol) was performed under solvent-free conditions for 120 min at different conditions.

^b^Isolated yield.

### Synthesis of thiadiazolo[2,3-b]quinazolin-6-one derivatives

**Figure 7:**
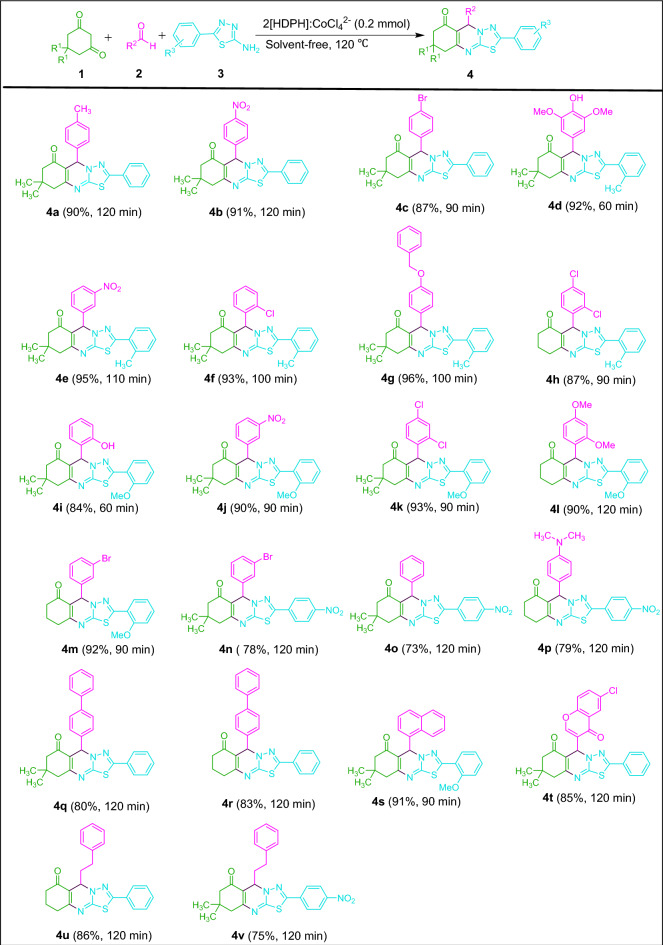
Synthesis of thiadiazolo[2,3-b]quinazolin-6-ones catalyzed by 2[HDPH]:CoCl_4_^2−^.

The applicability and scope of this protocol to the synthesis of thiadiazolo[2,3-b]quinazolin-6-one derivatives were then checked under optimal conditions. As shown in Fig. 7, the three-component reaction between dimedone/1,3-cyclohexanedione, 5-aryl-1,3,4-thiadiazol-2-amines and aromatic aldehydes with electron-donating and electron-withdrawing groups at various positions of aromatic ring proceeded smoothly in the presence of 2[HDPH]:CoCl_4_^2−^ at 120 ℃ under solvent-free conditions which afforded the corresponding thiadiazolo[2,3-b]quinazolin-6-ones **4a**–**4s** in 73–96% yields. Under the same reaction conditions, 6-chloro-4-oxo-4*H*-chromene-3-carbaldehyde as a heterocyclic aldehyde and 3-phenyl propanal, as an aliphatic aldehyde gave the desired products **4t**, **4u**, and **4v** in 85%, 86%, and 75% yields, respectively.

It is also interesting to note that a scale-up synthesis of **4a** was carried out under these conditions. On a 5 mmol scale, dimedone reacted with 4-methylbenzaldehyde and 5-phenyl-1,3,4-thiadiazol-2-amine in the presence of 2[HDPH]:CoCl_4_^2−^ at 120 ℃ under solvent-free conditions to afford the corresponding thiadiazolo[2,3-b]quinazolin-6-ones **4a** in 88% yields.

**Figure 8:**
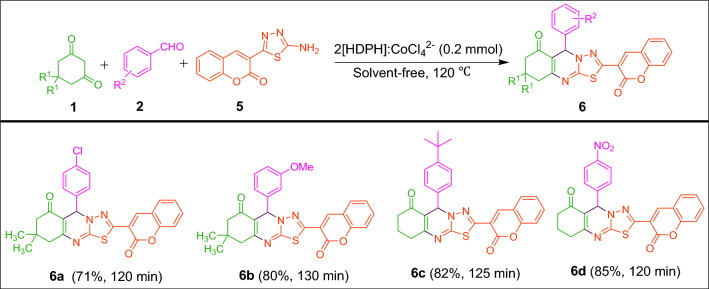
Synthesis of thiadiazolo[2,3-b]quinazolin-6-ones from 3-(5-amino-1,3,4-thiadiazol-2-yl)-2*H*-chromen-2-one catalyzed by 2[HDPH]:CoCl_4_^2−^.

To further demonstrate the efficacy of the present method, the synthesis of thiadiazolo[2,3-b]quinazolin-6-one derivatives was performed under optimal conditions using 3-(5-amino-1,3,4-thiadiazol-2-yl)-2*H*-chromen-2-one in place of 5-aryl-1,3,4-thiadiazol-2-amine and the corresponding products **6a–6d** were obtained in 71–85% yields (Fig. 8).

**Figure 9:**
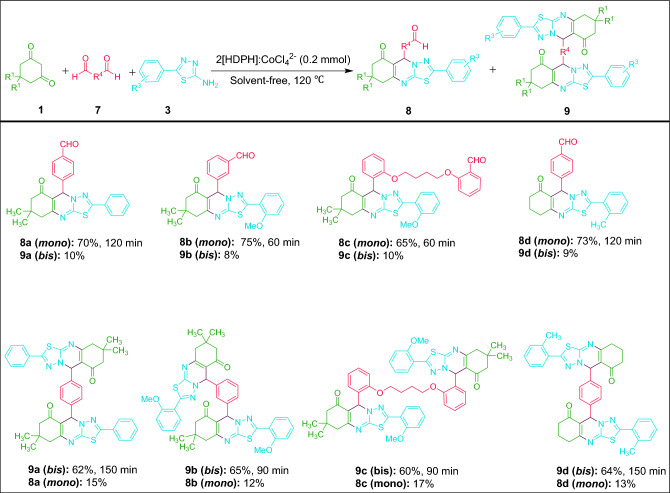
Synthesis of *mono*- and *bis*-thiadiazolo[2,3-b]quinazolin-6-ones from dialdehydes catalyzed by 2[HDPH]:CoCl_4_^2−^.

To spotlight the attractive performance of this catalytic system, *mono*- and *bis-*thiadiazolo[2,3-b]quinazolin-6-one scaffolds were prepared from different dialdehydes. As it is evident from Fig. 9, when dimedone/1,3-cyclohexanedione (1 mmol) reacted with 5-aryl-1,3,4-thiadiazol-2-amines (1 mmol) and dialdehydes (1 mmol) under the optimized conditions, the desired *mono*-thiadiazolo[2,3-b]quinazolin-6-one scaffolds were selectively produced in 65–75% yields. Also, the corresponding *bis*-thiadiazolo[2,3-b]quinazolin-6-ones were achieved in 60–65% yields by using 2 mmol dimedone/1,3-cyclohexanedione, 2 mmol 5-aryl-1,3,4-thiadiazol-2-amines, and 1 mmol dialdehydes under the same conditions.

**Figure 10:**
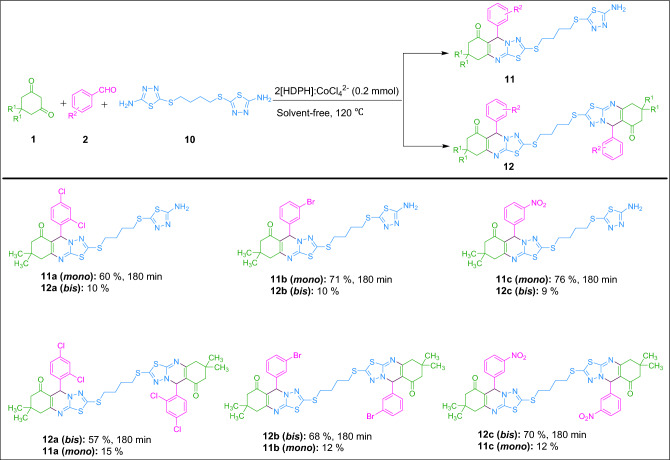
Synthesis of *mono*- and *bis*-thiadiazolo[2,3-b]quinazolin-6-ones from *bis*-1,3,4-thiadiazol-2-amine catalyzed by 2[HDPH]:CoCl_4_^2−^.

To reveal further utility of the current method, *mono*- and *bis*-thiadiazolo[2,3-b]quinazolin-6-ones were synthesized under optimal conditions using *bis*-1,3,4-thiadiazol-2-amine in place of dialdehyde**.** As observed in Fig. 10, the reaction of dimedone with aryl aldehyde and 5,5′-(butane-1,4-diylbis(sulfanediyl))bis(1,3,4-thiadiazol-2-amine) in 1:1:1 and 2:2:1 molar ratios in the presence of 2[HDPH]:CoCl_4_^2−^ at 120 ℃ under solvent-free conditions proceeded efficiently to generate the corresponding *mono*- and *bis*-thiadiazolo[2,3-b]quinazolin-6-ones in 60–76% and 57–70% yields, respectively. Finally, it should be stressed that all the above-mentioned findings reveal the brilliant eligibility and applicability of this catalytic system in the synthesis of these vital fused heterocyclic compounds.

**Figure 11:**
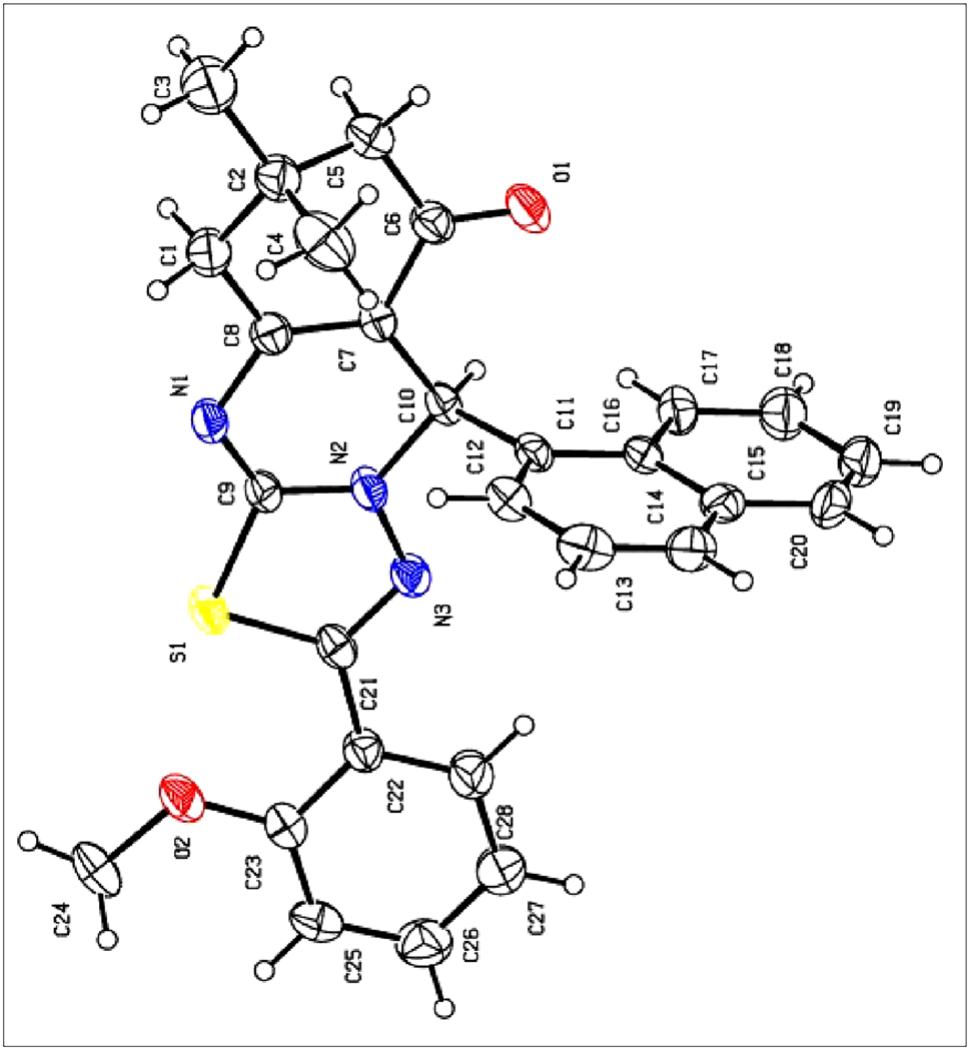
X-ray crystallographic structure of **4s**.

The structures of all the synthesized compounds were elucidated by FT-IR, ^1^H NMR, and ^13^C NMR spectra as well as elemental analysis. Furthermore, the structure of **4s** was confirmed by single crystal X-ray analysis (Fig. 11; CCDC 2297765, Tables S1 and S2).

**Figure 12:**
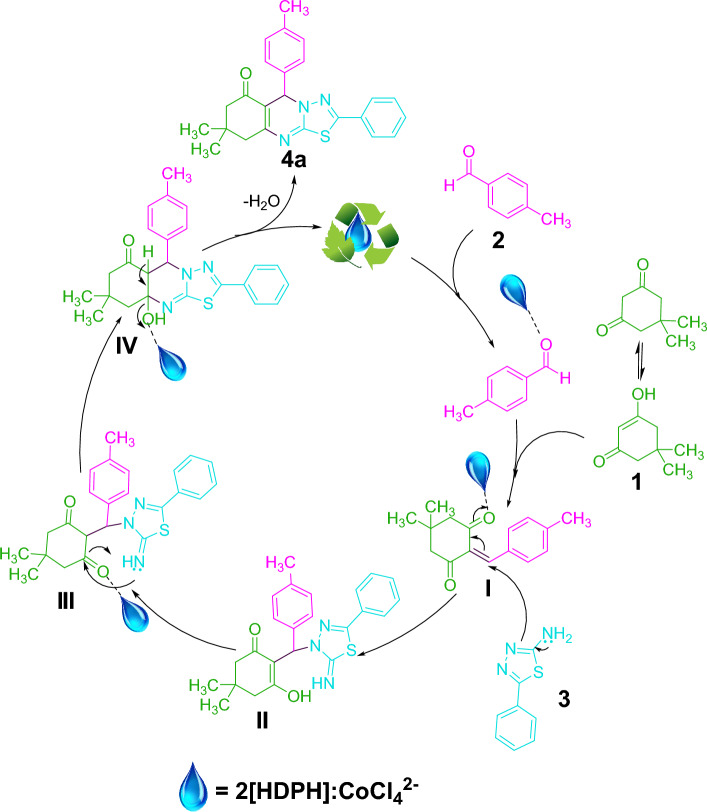
Proposed mechanism for the synthesis of **4a.**

A plausible reaction mechanism for the synthesis of **4a** in the presence of 2[HDPH]:CoCl_4_^2−^ is illustrated in Fig. 12. Initially, Knoevenagel condensation between the enol form of dimedone **1** and aldehyde **2** activated by the catalyst gives intermediate **Ӏ**. Then, LA-DES catalyzed Michael addition of 5-aryl-1,3,4-thiadiazol-2-amine **3** to intermediate **Ӏ** resulting in the formation of intermediate **II**, which is transformed to intermediate **IIӀ** after keto-enol tautomerism. Next, intermediate **ӀV** is formed by the cyclization of intermediate** IIӀ** in the presence of 2[HDPH]:CoCl_4_^2−^. Finally, the elimination of water from intermediate** ӀV** affords the final product **4a**, regenerating LA-DES, which can be used for the next catalytic cycle.

### Catalyst recycling and reuse

**Figure 13:**
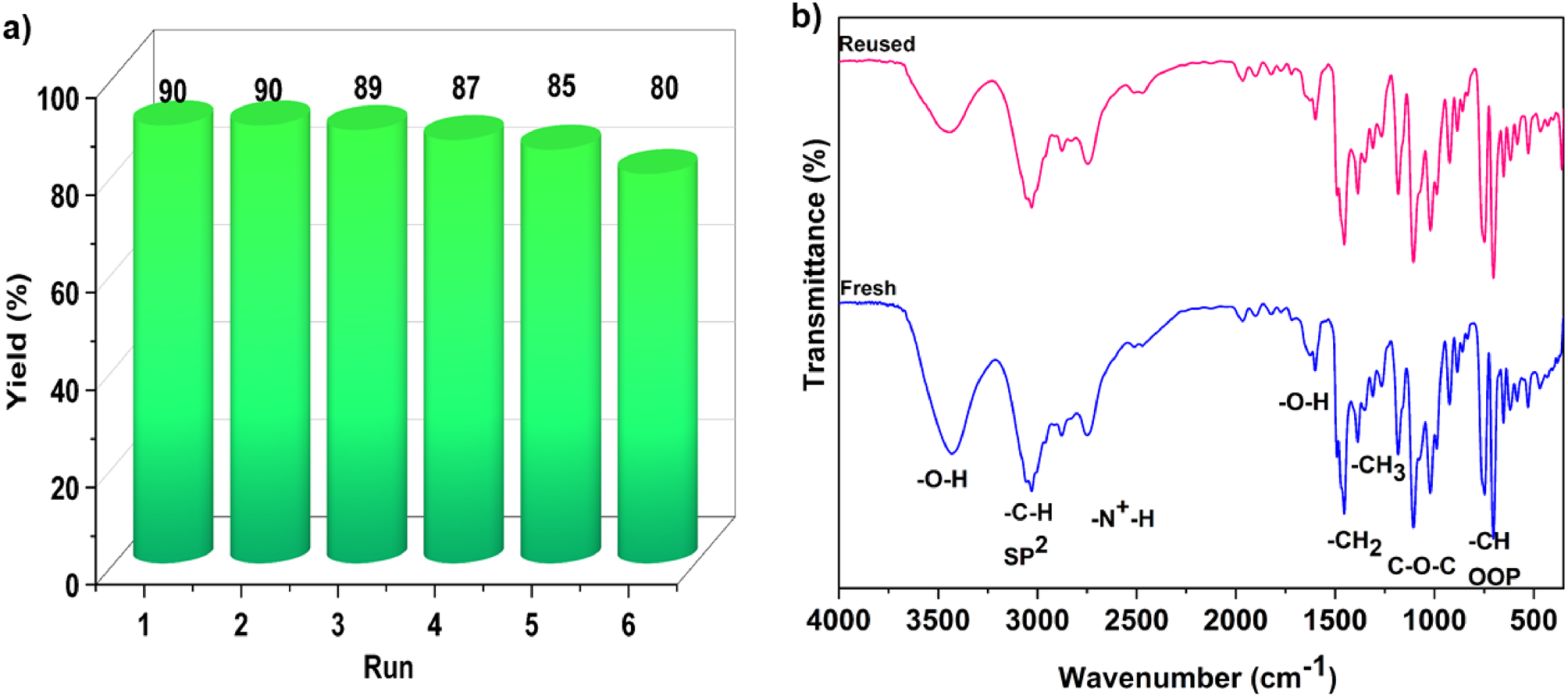
(**a**) Recovery and reuse of the catalyst for the synthesis of **4a**. (**b**) FT-IR spectra of fresh (blue) and reused (pink) of 2[HDPH]:CoCl_4_^2−^.

**Figure 14:**
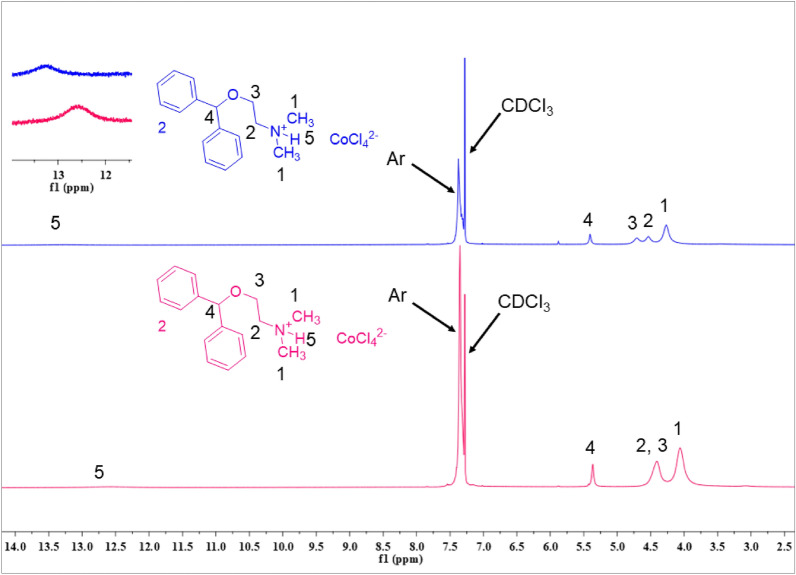
^1^H-NMR spectra of fresh (blue) and reused (pink) of 2[HDPH]:CoCl_4_^2−^.

Due to the atom economy, easy preparation, recovery, and reuse of DESs, they are considered green and sustainable solvents and catalysts in organic reactions. In this regard, the recyclability of the 2[HDPH]:CoCl_4_^2−^ was checked in the model reaction. After the consumption of precursors, the mixture was cooled to room temperature, water was added, and stirred to dissolve LA-DES. The resulting precipitate was collected by simple filtration followed by washing with water. The filtrate was evaporated at 80 °C under vacuum and the recovered LA-DES was reused for the subsequent cycle. As can be seen in Fig. 13a, the catalyst is reusable for up to six cycles without noticeable loss in its activity. Comparison of the FT-IR (Fig. 13b) and ^1^H-NMR spectra (Fig. 14) of the fresh and recovered LA-DES shows that the catalyst is stable during the reaction which is very important from the practical point of view.

### Green chemistry metric evaluation

**Figure 15:**
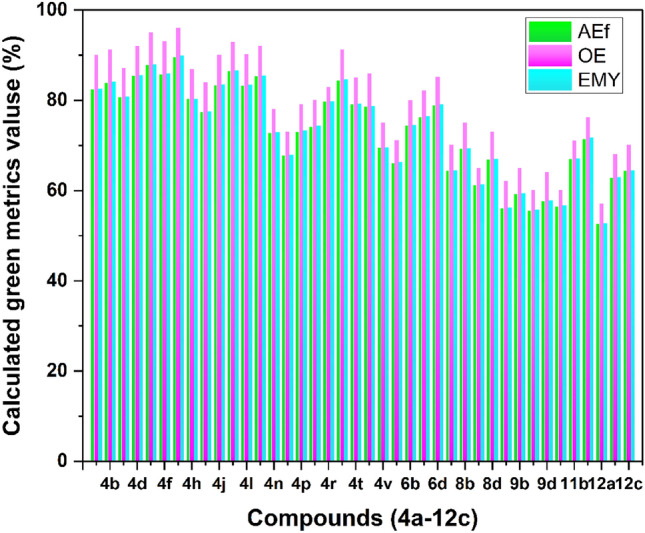
Schematic diagram of calculated green metrics values, AEf, OE, and EMY for the synthesized compounds (**4a**-**12c**).

**Figure 16:**
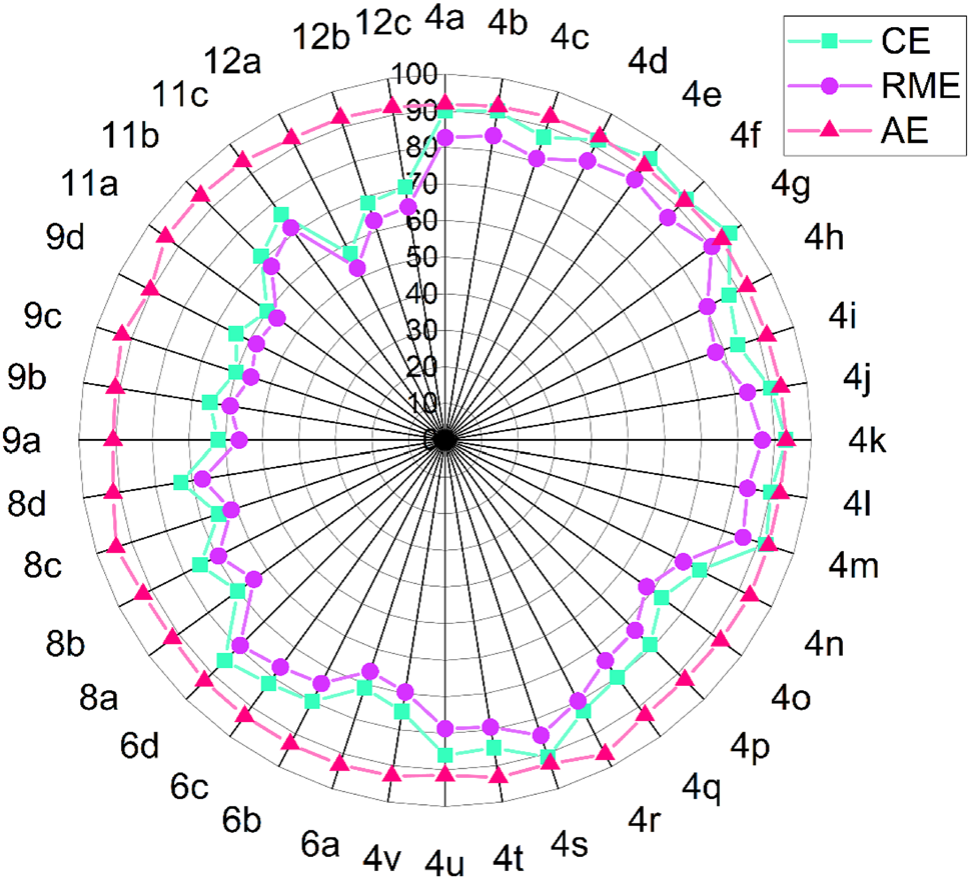
Radar plot of evaluated green chemistry metrics for compounds (**4a**-**12c**).

To introduce the existing method as an eco-friendly and green synthetic path for the preparation of thiadiazolo[2,3-b]quinazolin-6-one scaffolds, a number of green metric factors^50,53–56^ were calculated for the synthesized compounds (Figs. 15 and 16). In this respect, effective mass yield (EMY), and reaction mass efficiency (RME) for all the synthesized compounds were calculated and found to be in the range of 52.74–89.69%. To show the eco-compatibility and the atom economy of the present protocol, the E-factor, and atom economy (AE) were also determined which were found to be in ranges of 0.11–0.90 g/g and 90.27–96.18%, respectively. Due to the elimination of only water molecules in the present method, excellent results were obtained for the E-factor and atom economy. Also, the calculated atom efficiency (AEf), optimum efficiency (OE), and carbon efficiency (CE) for this procedure were up to 89.64%, 96.06%, and 96%, respectively. The obtained data together with the recoverability of the catalyst and solvent-free conditions introduce this protocol as a green and environmentally benign pathway for the preparation of thiadiazolo[2,3-b]quinazolin-6-ones. The detailed calculations are presented in the Supporting Information.

## Experimental

### General information

The chemicals were purchased from Fluka and Merck chemical companies. 5-aryl-1,3,4-thiadiazol-2-amines, 3-(5-amino-1,3,4-thiadiazol-2-yl)-2*H*-chromen-2-one, 5,5′-(butane-1,4-diylbis(sulfanediyl))bis(1,3,4-thiadiazol-2-amine) and 2,2′-(butane-1,4-diylbis(oxy))dibenzaldehyde were prepared similar to the reported methods^57–60^. Melting points were determined using a Stuart Scientific SMP2 apparatus. FT-IR spectra were recorded on a Nicolet-Impact 400D spectrophotometer. ^1^H and ^13^C NMR (400 and 100 MHz) spectra were recorded on a Bruker Avance 400 MHz spectrometer using CDCl_3_ solvent. Elemental analysis was performed on a LECO, CHNS-932 analyzer. Thermogravimetric and derivative thermogravimetric analysis (TGA/DTG) was carried out on a Perkin-Elmer STA 6000 instrument under nitrogen flow at a uniform heating rate of 10 ℃ min^−1^ in the range of 30–600 ℃. Differential scanning calorimetry (DSC) was carried out with a TA Instrument Model DSC13-setaram under a nitrogen atmosphere with a scan rate of 10 ℃ min^−1^ in the range − 60 to 20 ℃.

### General procedure for the synthesis of Lewis acid-based deep eutectic solvents (LA-DESs)

Diphenhydramine hydrochloride was mixed with the metal chloride hydrate at the specified molar ratio (Table 2) and heated up to 90 ℃ with mild stirring until it turned into a transparent liquid. The synthesized LA-DES, after cooling, was utilized without any further purification.

**Table 2:** The states of different catalysts.

Entry	Catalyst	Ratio	State at Room Temperature
1	[HDPH]Cl:CoCl_2_·6H_2_O	1:2	No clear melt
2	[HDPH]Cl:CoCl_2_·6H_2_O	1:1	No clear melt
3	[HDPH]Cl:CoCl_2_·6H_2_O	2:1	Liquid
4	[HDPH]Cl:CoCl_2_·6H_2_O	3:1	Liquid
5	[HDPH]Cl:CoCl_2_·6H_2_O	4:1	Liquid
6	[HDPH]Cl:CoCl_2_·6H_2_O	5:1	No clear melt
7	[HDPH]Cl:NiCl_2_·6H_2_O	2:1	No clear melt
8	[HDPH]Cl:CuCl_2_·2H_2_O	2:1	Liquid
9	[HDPH]Cl:MnCl_2_·2H_2_O	2:1	Liquid
10	[HDPH]Cl:MgCl_2_·6H_2_O	2:1	White solid

### Synthesis of thiadiazolo[2,3-b]quinazolin-6-ones catalyzed by 2[HDPH]:CoCl_4_^2−^

In a screw-cap glass tube with a magnetic stirrer, a mixture of dimedone/1,3-cyclohexanedione **1** (1 mmol), aldehyde **2** (1 mmol), 5-substituted-1,3,4-thiadiazol-2-amine **3** or **5** (1 mmol) and 2[HDPH]:CoCl_4_^2−^ (0.2 mmol) was heated at 120 ℃ under solvent-free conditions for the appropriate time mentioned in Figs. 7 and 8. Upon consumption of the precursors as depicted by TLC (eluent: *n*-hexane/EtOAc, 2:1), the reaction mixture was allowed to cool to room temperature. To dissolve 2[HDPH]:CoCl_4_^2−^, water (5 mL) was added, and the resulting solid crude product was separated by simple filtration, washed with water (5 mL), dried, and crystallized from ethyl acetate to afford the pure product. In some cases, chromatography on silica gel (eluent: *n*-hexane/EtOAc, 2:1) is required to obtain the pure product.

### Synthesis of ***mono*** and ***bis***-thiadiazolo[2,3-b]quinazolin-6-ones from dialdehyde catalyzed by 2[HDPH]:CoCl_4_^2−^

In a screw-cap glass tube with a magnetic stirrer, a mixture of dimedone/1,3-cyclohexanedione **1** (1 mmol), dialdehyde **7** (1 mmol), 5-aryl-1,3,4-thiadiazol-2-amine **3** (1 mmol) and 2[HDPH]:CoCl_4_^2−^ (0.2 mmol) was heated at 120 ℃ under solvent-free conditions for the specified time demonstrated in Fig. 9. After completion of the reaction (monitored by TLC; eluent: *n*-hexane/EtOAc, 2:1), the workup was carried out according to the procedure used for thiadiazolo[2,3-b]quinazolin-6-ones to afford the pure mono-derivatives **8a**–**8d** in 70–75% yields. For the synthesis of *bis*-thiadiazolo[2,3-b]quinazolin-6-ones, dimedone/1,3-cyclohexanedione **1** (1 mmol) reacted with dialdehyde **7** (0.5 mmol) and 5-aryl-1,3,4-thiadiazol-2-amine **3** (1 mmol) in the presence of LA-DES (0.2 mmol) and the mixture was heated at 120 ℃ under solvent-free conditions. The reaction progress was checked by TLC (eluent: *n*-hexane/EtOAc, 2:1). The workup and purification were performed according to the above-mentioned procedure to furnish the desired products** 9a-9d** in 60–65% yields (Fig. 9).

### Synthesis of *mono* and *bis*-thiadiazolo[2,3-b]quinazolin-6-ones from* bis*-1,3,4-thiadiazol-2-amine catalyzed by 2[HDPH]:CoCl_4_^2−^

In a screw-cap glass tube with a magnetic stirrer, dimedone **1** (1 mmol), aldehyde **2** (1 mmol), and 5,5′-(butane-1,4-diylbis(sulfanediyl))bis(1,3,4-thiadiazol-2-amine)** 10** (1 mmol) were added to 2[HDPH]:CoCl_4_^2−^ (0.2 mmol) and the mixture was heated at 120 ℃ under solvent-free conditions for the specified time indicated in Fig. 10. On completion of the reaction (monitored by TLC analysis; eluent: *n*-hexane/EtOAc, 2:1), the workup was carried out according to the procedure for the preparation of thiadiazolo[2,3-b]quinazolin-6-one and the desired pure product **11a**–**11c** were obtained in 60–76% yields. All the steps for the synthesis of *bis*-thiadiazolo[2,3-b]quinazolin-6-ones (**12a**–**12c**, 57–70%) from *bis*-1,3,4-thiadiazol-2-amine and their purification were the same as those of *mono*-thiadiazolo[2,3-b]quinazolin-6-ones except that 0.5 mmol of 5,5′-(butane-1,4-diylbis(sulfanediyl))bis(1,3,4-thiadiazol-2-amine) reacted with 1 mmol of each dimedone **1** and aldehyde **2** (Fig. 10).

## Conclusions

In summary, an efficient, convenient, green, and straightforward protocol has been developed for the preparation of a series of thiadiazolo[2,3-b]quinazolin-6-ones via a one-pot, single-step, multicomponent reaction of dimedone/1,3-cyclohexanone, aldehydes, and 5-aryl-1,3,4-thiadiazol-2-amines/3-(5-amino-1,3,4-thiadiazol-2-yl)-2*H*-chromen-2-one) in the presence of 2[HDPH]:CoCl_4_^2−^ as an LA-DES under solvent-free conditions. This effective process is also applicable to the synthesis of *mono*- and *bis*-thiadiazolo[2,3-b]quinazolin-6-ones from dialdehydes or *bis*-1,3,4-thiadiazol-2-amine. High to excellent yields, high reaction rates, avoiding toxic organic solvents, operational simplicity, easy separation and recyclability of the catalyst, large-scale synthetic applicability, formation of water as green waste, excellent atom economy, high reaction mass efficiency, and low E-factor are outstanding features of this protocol.

### Supplementary Information

Supplementary Information.

## Data Availability

All data generated or analyzed during this study are included in this published article and its supplementary information files.
