# Supplementary material for: Efficient synthesis of novel thiadiazolo[2,3-b]quinazolin-6-ones catalyzed by diphenhydramine hydrochloride-CoCl2⋅6H2O deep eutectic solvent
Source: Sci Rep. 2024 Jan 16;14:1451. doi: 10.1038/s41598-024-52017-3 (PMC10791603; doi:10.1038/s41598-024-52017-3)
Supplement: Supplementary file 1 — Supplementary Information. [file 41598_2024_52017_MOESM1_ESM.docx]

**Supporting Information**

**Efficient synthesis of novel thiadiazolo[2,3-b]quinazolin-6-ones catalyzed by diphenhydramine hydrochloride-CoCl.6H_2_O deep eutectic solvent**

Mehri Moeini Korbekandi^1^, Iraj Mohammadpoor-Baltork^1^*, Majid Moghadam^1^, Shahram Tangestaninejad^1^, Valiollah Mirkhani^1^, Behrouz Notash^2^

**^1^**Department of Chemistry, Catalysis Division, University of Isfahan, Isfahan 81746-73441, Iran.

^2^Department of Inorganic Chemistry, Shahid Beheshti University, Tehran, Iran.

**^*^Corresponding author:**

Iraj Mohammadpoor-Baltork

E-mail address: imbaltork@sci.ui.ac.ir

Fax: +98-031-36689732

Tel: +98-031-37932749

**Table of contents**

Spectroscopic data of the products……………………………………………………...S3-S19

^1^H and ^13^C NMR Spectra of the products………………………………………….…... S20-S102

X-ray crystallography structure of **4s** ……………….…….…………………………...S103

Crystal data and structure refinement for **4s**………………………………………….....S104

Bond lengths [Å] and angles [deg] for **4s**……………………………………….............S105-S108

Validation of green chemistry metrics for all synthesized compounds………….…..…S108-S111

Reference……………………………………………………………………………..…S112

# **Spectroscopic data of the products**

**8,8-Dimethyl-2-phenyl-5-(p-tolyl)-5,7,8,9-tetrahydro-6H-[1,3,4]thiadiazolo[2,3-b]quinazolin-6-one: (4a)**

Yield: (361.38 mg, 90 %); Yellow solid; Mp: 198-200 ˚C (Lit.^1^ 203-205 ˚C). FT-IR (KBr): *ν*_max_ = 2938, 1644, 1586, 1498, 1374, 1245, 1126, 761 cm^−1^. ^1^H NMR (400 MHz, CDCl_3_): *δ* = 7.67 (d, *J* = 6.8 Hz, 2H, Ar-H), 7.50-7.42 (m, 3H, Ar-H), 7.34 (d, *J* = 8.0 Hz, 2H, Ar-H), 7.14 (d, *J* = 8.0 Hz, 2H, Ar-H), 6.49 (s, 1H, -CH), 2.53 (ABq, *J* = 17.6 Hz, 2H, -CH_2_), 2.31 (s, 3H, -CH_3_), 2.28 (s, 1H), 2.23 (d, *J* = 16.0 Hz, 1H), 1.13 (s, 3H, -CH_3_), 1.07 (s, 3H, -CH_3_). ^13^C NMR (100 MHz, CDCl_3_): *δ* = 195.49, 165.00, 158.70, 153.43, 138.88, 138.15, 131.52, 129.33, 129.09, 129.01, 127.25, 126.50, 110.81, 60.35, 50.73, 45.35, 32.65, 29.31, 27.66, 21.20.

**8,8-Dimethyl-5-(4-nitrophenyl)-2-phenyl-5,7,8,9-tetrahydro-6H-[1,3,4] thiadiazolo[2,3-b]quinazolin-6-one: (4b)**

Yield: (393.58 mg, 91 %); Yellow solid; Mp: 156-158 ˚C. FT-IR (KBr): *ν*_max_ = 3105, 2938, 1626, 1596, 1508, 1343, 1271, 1135, 854 cm^−1^. ^1^H NMR (400 MHz, CDCl_3_): *δ* = 8.22-8.20 (m, 2H, Ar-H), 7.67-7.65 (m, 2H, Ar-H), 7.63-7.60 (m, 2H, Ar-H), 7.53-7.46 (m, 1H, Ar-H), 7.47-7.43 (m, 2H, Ar-H), 6.62 (s, 1H, -CH), 2.43 (q, *J* = 18.0 Hz, 2H, -CH_2_), 2.27 (ABq, *J* = 16.4 Hz, 2H, -CH_2_), 1.14 (s, 3H, -CH_3_), 1.04 (s, 3H, -CH_3_). ^13^C NMR (100 MHz, CDCl_3_): *δ* = 195.42, 165.29, 159.45, 154.32, 148.08, 147.73, 131.95, 129.25, 128.57, 128.29, 126.53, 124.03, 109.90, 60.15, 50.59, 45.35, 32.67, 29.19, 27.60. Anal. Calcd for C_23_H_20_N_4_O_3_S: C, 63.87; H, 4.66; N, 12.95; S, 7.41. Found: C, 63.96; H, 4.69; N, 12.90; S, 7.34.

**5-(4-Bromophenyl)-8,8-dimethyl-2-phenyl-5,7,8,9-tetrahydro-6H-[1,3,4]thiadiazolo[2,3-b] quinazolin-6-one: (4c)**

Yield: (405.77 mg, 87%); Yellow solid; Mp: 210-212 ˚C (Lit.^1^ 205-207 ˚C). FT-IR (KBr): *ν*_max_ = 2940, 1641, 1585, 1503, 1374, 1240, 1128, 760 cm^−1^. ^1^H NMR (400 MHz, CDCl_3_): *δ* = 7.67-7.65 (m, 2H, Ar-H), 7.53-7.42 (m, 5H, Ar-H), 7.33-7.30 (m, 2H, Ar-H), 6.48 (s, 1H, -CH), 2.52 (ABq, *J* = 17.6 Hz, 2H, -CH_2_), 2.26 (ABq, *J* = 16.4 Hz, 2H, -CH_2_), 1.13 (s, 3H, -CH_3_), 1.05 (s, 3H, -CH_3_). ^13^C NMR (100 MHz, CDCl_3_): *δ* = 195.44, 165.10, 158.98, 153.80, 140.66, 131.81, 131.72, 129.17, 129.04, 128.82, 126.52, 122.47, 110.34, 60.12, 50.67, 45.34, 32.65, 29.26, 27.63.

**5-(4-Hydroxy-3,5-dimethoxyphenyl)-8,8-dimethyl-2-(o-tolyl)-5,7,8,9-tetrahydro-6H-[1,3,4]thiadiazolo[2,3-b]quinazolin-6-one: (4d)**

Yield: (439.37 mg, 92 %); Yellow solid; Mp: 202-204 ˚C. FT-IR (KBr): *ν*_max_ = 2927, 1617, 1580, 1516, 1472, 1378, 1231, 1116, 758 cm^−1^. ^1^H NMR (400 MHz, CDCl_3_): *δ* = 7.43-7.39 (m, 1H, Ar-H), 7.37 (d, *J* = 7.6 Hz, 1H, Ar-H), 7.30 (s, 1H, Ar-H), 7.26 (s, 1H, Ar-H), 6.68 (s, 2H, Ar-H), 6.45 (s, 1H, -CH), 5.62 (s, 1H, -OH), 3.88 (s, 6H, OCH_3_), 2.57 (s, 2H, -CH_2_), 2.45 (s, 3H, -CH_3_), 2.31 (ABq, *J* = 16.4 Hz, 2H, -CH_2_), 1.16 (d, *J* = 3.6 Hz, 6H, -CH_3_). ^13^C NMR (100 MHz, CDCl_3_): *δ* = 195.56, 164.77, 159.23, 153.67, 147.05, 137.33, 134.91, 132.82, 131.99, 130.89, 129.55, 127.78, 126.44, 110.17, 104.23, 60.68, 56.24, 50.69, 45.41, 32.59, 29.69, 27.34, 21.78. Anal. Calcd for C_26_H_27_N_3_O_4_S: C, 65.39; H, 5.70; N, 8.80, S, 6.71. Found: C, 65.30; H, 5.73; N, 8.74, S, 6.79.

**8,8-Dimethyl-5-(3-nitrophenyl)-2-(o-tolyl)-5,7,8,9-tetrahydro-6H-[1,3,4] thiadiazol[2,3-b]quinazolin-6-one: (4e)**

Yield: (424.20 mg, 95 %); Yellow solid; Mp: 166-168 ˚C. FT-IR (KBr): *ν*_max_ = 2956, 1634, 1582, 1528, 1489, 1376, 1237, 1132, 758 cm^−1^. ^1^H NMR (400 MHz, CDCl_3_): *δ* = 8.29-8.28 (m, 1H, Ar-H), 8.17-8.14 (m, 1H, Ar-H), 7.81 (d, *J* = 8.0 Hz, 1H, Ar-H), 7.54 (t, *J* = 8.0 Hz, 1H, Ar-H), 7.43-7.36 (m, 2H, Ar-H), 7.30 (s, 1H, Ar-H), 7.26 (s, 1H, Ar-H), 6.64 (s, 1H, -CH), 2.58 (ABq, *J* = 17.6 Hz, 2H, -CH_2_), 2.40 (s, 3H, -CH_3_), 2.29 (ABq, *J* = 16.4 Hz, 2H, -CH_2_), 1.15 (s, 3H, -CH_3_), 1.11 (s, 3H, -CH_3_). ^13^C NMR (100 MHz, CDCl_3_): *δ* = 195.50, 165.15, 159.96, 154.42, 148.53, 143.30, 137.37, 133.64, 132.08, 131.16, 129.61, 129.54, 127.47, 126.52, 123.43, 122.41, 109.39, 60.12, 50.60, 45.39, 32.69, 29.19, 27.78, 21.62. Anal. Calcd for C_24_H_22_N_4_O_3_S: C, 64.56; H, 4.97; N, 12.55; S, 7.18. Found: C, 64.66; H, 4.95; N, 12.61; S, 7.12.

**5-(2-Chlorophenyl)-8,8-dimethyl-2-(o-tolyl)-5,7,8,9-tetrahydro-6H-[1,3,4]thiadiazolo[2,3-b]quinazolin-6-one: (4f)**

Yield: (405.45 mg, 93 %); Yellow solid; Mp: 172-174 ˚C. FT-IR (KBr): *ν*_max_ = 2949, 1637, 1587, 1490, 1375, 1239, 1135, 759 cm^−1^. ^1^H NMR (400 MHz, CDCl_3_): *δ* = 7.51 (dd, ^1^*J* = 7.6 Hz, ^2^*J* = 1.6 Hz, 1H, Ar-H), 7.41-7.33 (m, 3H, Ar-H), 7.27 (d, *J* = 7.6 Hz, 2H, Ar-H), 7.24-7.19 (m, 2H, Ar-H), 6.96 (s, 1H, -CH), 2.55 (ABq, *J* = 17.6 Hz, 2H, -CH_2_), 2.37 (s, 3H, -CH_3_), 2.26 (ABq, *J* = 16.4 Hz, 2H, -CH_2_), 1.14 (s, 3H, -CH_3_), 1.10 (s, 3H, -CH_3_). ^13^C NMR (100 MHz, CDCl_3_): *δ* = 195.33, 165.32, 159.59, 153.48, 138.76, 137.68, 133.24, 132.03, 130.83, 130.48, 129.94, 129.52, 129.43, 127.68, 127.11, 126.33, 109.83, 58.75, 50.63, 45.44, 32.59, 29.23, 27.72, 21.62. Anal. Calcd for C_24_H_22_ClN_3_OS: C, 66.12; H, 5.09; N, 9.64; S, 7.35. Found: C, 66.20; H, 5.12; N, 9.58; S, 7.42.

**5-(4-(Benzyloxy)phenyl)-8,8-dimethyl-2-(o-tolyl)-5,7,8,9-tetrahydro-6H-[1,3,4]thiadiazolo [2,3-b]quinazolin-6-one: (4g)**

Yield: (487.34 mg, 96 %); Yellow solid; Mp: 176-178 ˚C. FT-IR (KBr): *ν*_max_ = 2959, 1636, 1583, 1480, 1370, 1235, 1132, 746 cm^−1^. ^1^H NMR (400 MHz, CDCl_3_): *δ* = 7.43-7.41 (m, 3H, Ar-H), 7.39-7.33 (m, 5H, Ar-H), 7.30-7.23 (m, 3H, Ar-H), 6.93 (d, *J* = 8.8 Hz, 2H, Ar-H), 6.49 (s, 1H, -CH), 5.03 (s, 2H, -CH_2_), 2.55 (ABq, *J* = 17.6 Hz, 2H, -CH_2_), 2.42 (s, 3H, -CH_3_), 2.28 (ABq, *J* = 16.4 Hz, 2H, -CH_2_), 1.14 (s, 3H, -CH_3_), 1.10 (s, 3H, -CH_3_). ^13^C NMR (100 MHz, CDCl_3_): *δ* = 195.54, 164.98, 158.98, 158.82, 153.49, 137.45, 136.88, 134.37, 131.95, 130.80, 129.49, 128.61, 128.58, 127.98, 127.87, 127.51, 126.36, 114.81, 110.56, 69.99, 60.00, 50.76, 45.40, 32.65, 29.28, 27.80, 21.66. Anal. Calcd for C_31_H_29_N_3_O_2_S: C, 73.35; H, 5.76; N, 8.28; S, 6.32. Found: C, 73.25; H, 5.74; N, 8.23; S, 6.40.

**5-(2,4-Dichlorophenyl)-2-(o-tolyl)-5,7,8,9-tetrahydro-6H-[1,3,4]thiadiazolo [2,3-b]quinazolin-6-one:(4h)**

Yield: (384.85mg, 87 %); Yellow solid; Mp: 164-166 ˚C. FT-IR (KBr): *ν*_max_ = 2945, 1639, 1579, 1481, 1366, 1236, 1150, 1121, 841, 762 cm^−1^. ^1^H NMR (400 MHz, CDCl_3_): *δ* = 7.44-7.34 (m, 4H, Ar-H), 7.29 (s, 1H, Ar-H), 7.27 (s, 1H, Ar-H), 7.23 (dd, ^1^*J* = 8.4 Hz, ^2^*J* = 2.0 Hz, 1H, Ar-H), 6.91 (s, 1H, -CH), 2.75-2.58 (m, 2H, -CH_2_), 2.41-2.38 (m, 5H, -CH_2_ and -CH_3_), 2.11-2.00 (m, 2H, -CH_2_). ^13^C NMR (100 MHz, CDCl_3_): *δ* = 195.56, 165.01, 161.66, 153.82, 137.62, 137.38, 134.69, 134.09, 132.10, 131.34, 130.98, 129.69, 129.47, 127.54, 126.41, 110.61, 58.26, 36.97, 31.62, 21.73, 21.52. Anal. Calcd for C_22_H_17_Cl_2_N_3_OS: C, 59.73; H, 3.87; N, 9.50; S, 7.25. Found: C, 59.81; H, 3.84; N, 9.44; S, 7.32.

**5-(2-Hydroxyphenyl)-2-(2-methoxyphenyl)-8,8-dimethyl-5,7,8,9-tetrahydro-6H-[1,3,4]thiadiazolo[2,3-b]quinazolin-6-one: (4i)**

Yield: (364.17 mg, 84 %); Yellow solid; Mp: 244-246 ˚C. FT-IR (KBr): *ν*_max_ = 2953, 1636, 1587, 1486, 1460, 1376, 1241, 1122, 755 cm^−1^. ^1^H NMR (400 MHz, CDCl_3_): *δ* = 9.81 (s, 1H, -OH), 8.06 (dd, ^1^*J* = 8.0 Hz, ^2^*J* =1.6 Hz, 1H, Ar-H), 7.57-7.53 (m, 1H, Ar-H), 7.27-7.22 (m, 1H, Ar-H), 7.13-7.09 (m, 3H, Ar-H), 6.92 (s, 1H, -CH), 6.89 (dd, ^1^*J* = 7.2 Hz, ^2^*J* = 0.8 Hz, 1H, Ar-H), 6.87-6.84 (m, 1H, Ar-H), 4.10 (s, 3H, -OCH_3_), 2.61 (d, *J* = 17.2 Hz, 1H, -CH_2_), 2.55 (d, *J* = 17.6 Hz, 1H, -CH_2_), 2.43 (ABq, *J* = 16.4 Hz, 2H, -CH_2_), 1.20 (s, 3H, -CH_3_), 1.05 (s, 3H, -CH_3_). ^13^C NMR (100 MHz, CDCl_3_): *δ* = 197.71, 167.60, 162.80, 156.35, 152.33, 150.56, 132.93, 130.39, 129.31, 127.83, 126.04, 121.42, 121.14, 119.51, 117.48, 111.48, 109.86, 55.86, 54.86, 50.11, 45.76, 32.70, 28.91, 27.49. Anal. Calcd for C_24_H_23_N_3_O_3_S: C, 66.49; H, 5.35; N, 9.69; S, 7.40. Found: C, 66.59; H, 5.37; N, 9.64; S, 7.33.

**2-(2-Methoxyphenyl)-8,8-dimethyl-5-(3-nitrophenyl)-5,7,8,9-tetrahydro-6H-[1,3,4]thiadiazolo[2,3-b]quinazolin-6-one: (4j)**

Yield: (416.27 mg, 90 %); Yellow solid; Mp: 194-196 ˚C. FT-IR (KBr): *ν*_max_ = 2945, 1636, 1587, 1532, 1484, 1378, 1246, 1118, 752 cm^−1^. ^1^H NMR (400 MHz, CDCl_3_): *δ* = 8.28 (t, *J* = 1.6 Hz, 1H, Ar-H), 8.13 (dd, ^1^*J* = 8.0 Hz, ^2^*J* = 1.2 Hz, 1H, Ar-H), 7.99-7.97 (m, 1H, Ar-H), 7.81 (d, *J* = 7.6 Hz, 1H, Ar-H), 7.53-7.44 (m, 2H, Ar-H), 7.07-7.00 (m, 2H, Ar-H), 6.65 (s, 1H, -CH), 3.98 (s, 3H, -OCH_3_), 2.57 (ABq, *J* = 17.6 Hz, 2H, -CH_2_), 2.27 (ABq, *J* = 16.4 Hz, 2H, -CH_2_), 1.14 (s, 3H, -CH_3_), 1.08 (s, 3H, -CH_3_). ^13^C NMR (100 MHz, CDCl_3_): *δ* = 195.27, 165.88, 160.34, 156.42, 150.23, 148.49, 143.78, 133.65, 132.93, 129.51, 127.51, 123.25, 122.41, 121.40, 117.54, 111.61, 108.85, 60.05, 55.86, 50.62, 45.50, 32.66, 29.28, 27.67. Anal. Calcd for C_24_H_22_N_4_O_4_S: C, 62.32; H, 4.79; N, 12.11; S, 6.93. Found: C, 62.23; H, 4.81; N, 12.05; S, 7.00.

**5-(2,4-Dichlorophenyl)-2-(2-methoxyphenyl)-8,8-dimethyl-5,7,8,9-tetrahydro-6H-[1,3,4]thiadiazolo[2,3-b]quinazolin-6-one: (4k)**

Yield: (452.41 mg, 93 %); Yellow solid; Mp: 227-229 ˚C. FT-IR (KBr): *ν*_max_ = 2953, 1636, 1582, 1464, 1376, 1244, 1118, 751 cm^−1^. ^1^H NMR (400 MHz, CDCl_3_): *δ* = 7.97 (dd, ^1^*J* = 7.6 Hz, ^2^*J* = 1.6 Hz, 1H, Ar-H), 7.47-7.43 (m, 2H, Ar-H), 7.38 (d, *J* = 2.0 Hz, 1H, Ar-H), 7.22 (dd, ^1^*J* = 8.4 Hz, ^2^*J* = 2.0 Hz, 1H, Ar-H), 7.05 (t, *J* = 7.6 Hz, 1H, Ar-H), 6.99 (d, *J* = 8.4 Hz, 1H, Ar-H), 6.90 (s, 1H, -CH), 3.97 (s, 3H, -OCH_3_), 2.53 (s, 2H, -CH_2_), 2.24 (ABq, *J* = 16.4 Hz, 2H, -CH_2_), 1.13 (s, 3H, -CH_3_), 1.08 (s, 3H, -CH_3_). ^13^C NMR (100 MHz, CDCl_3_): *δ* = 195.15, 166.00, 160.34, 156.40, 149.56, 137.63, 134.51, 133.97, 132.72, 131.58, 129.81, 127.59, 127.34, 121.38, 117.68, 111.55, 108.69, 58.45, 55.83, 50.61, 45.52, 32.55, 29.30, 27.77. Anal. Calcd for C_24_H_21_Cl_2_N_3_O_2_S: C, 59.26; H, 4.35; N, 8.64; S, 6.59. Found: C, 59.18; H, 4.32; N, 8.69; S, 6.66.

**5-(2,4-Dimethoxyphenyl)-2-(2-methoxyphenyl)-5,7,8,9-tetrahydro-6H-[1,3,4]thiadiazolo[2,3-b]quinazolin-6-one: (4l)**

Yield: (404.58mg, 90 %); Yellow solid; Mp: 223-225 ˚C. FT-IR (KBr): *ν*_max_ = 2930, 1623, 1577, 1466, 1375, 1303, 1242, 1158, 1115, 754 cm^−1^. ^1^H NMR (400 MHz, CDCl_3_): *δ* = 7.96 (d, *J* = 8.0 Hz, 1H, Ar-H), 7.43-7.38 (m, 2H, Ar-H), 7.02-6.96 (m, 2H, Ar-H), 6.72 (s, 1H, -CH), 6.45 (dd, ^1^*J* = 8.4 Hz, ^2^*J* = 2.4 Hz, 1H, Ar-H), 6.42 (d, *J* = 2.4 Hz, 1H, Ar-H), 3.95 (s, 3H, -OCH_3_), 3.82 (s, 3H, -OCH_3_), 3.77 (s, 3H, -OCH_3_), 2.71-2.55 (m, 2H, -CH_2_), 2.37-2.33 (m, 2H, -CH_2_), 2.04-1.94 (m, 2H, -CH_2_). ^13^C NMR (100 MHz, CDCl_3_): *δ* = 195.43, 165.63, 160.80, 158.80, 156.25, 132.37, 130.87, 130.86, 127.63, 122.97, 122.95, 121.20, 118.00, 111.49, 110.34, 104.25, 99.07, 57.23, 55.80, 55.77, 55.28, 37.18, 31.48, 21.62. Anal. Calcd for C_24_H_23_N_3_O_4_S: C, 64.13; H, 5.16; N, 9.35; S, 7.13. Found: C, 64.22; H, 5.14; N, 9.42; S, 7.05.

**5-(3-Bromophenyl)-2-(2-methoxyphenyl)-5,7,8,9-tetrahydro-6H-[1,3,4]thiadiazolo[2,3-b]quinazolin-6-one: (4m)**

Yield: (430.90 mg, 92 %); Yellow solid; Mp: 243-245 ˚C. FT-IR (KBr): *ν*_max_ = 2935, 1628, 1575, 1484, 1375, 1241, 1157, 1113, 759 cm^−1^. ^1^H NMR (400 MHz, CDCl_3_): *δ* = 8.00 (dd, ^1^*J* = 8.0 Hz, ^2^*J* = 1.6 Hz, 1H, Ar-H), 7.54 (t, *J* = 1.6 Hz, 1H, Ar-H), 7.48-7.43 (m, 1H, Ar-H), 7.39-7.36 (m, 2H, Ar-H), 7.21-7.17 (m, 1H, Ar-H), 7.08-7.04 (m, 1H, Ar-H), 7.00 (d, *J* = 8.4 Hz, 1H, Ar-H), 6.54 (s, 1H, -CH), 3.97 (s, 3H, -OCH_3_), 2.77-2.57 (m, 2H, -CH_2_), 2.47-2.34 (m, 2H, -CH_2_), 2.10-2.00 (m, 2H, -CH_2_). ^13^C NMR (100 MHz, CDCl_3_): *δ* = 194.93, 155.89, 143.40, 132.26, 132.26, 130.86, 130.85, 129.84, 129.59, 129.13, 127.12, 125.65, 122.26, 120.88, 117.21, 111.08, 109.89, 59.26, 55.34, 36.55, 31.13, 20.99. Anal. Calcd for C_22_H_18_BrN_3_O_2_S: C, 56.42; H, 3.87; N, 8.97; S, 6.85. Found: C, 56.52; H, 3.90; N, 8.91; S, 6.92.

**5-(3-Bromophenyl)-8,8-dimethyl-2-(4-nitrophenyl)-5,7,8,9-tetrahydro-6H-[1,3,4]thiadiazolo[2,3-b]quinazolin-6-one: (4n)**

Yield: (398.88 mg, 78 %); Orange solid; Mp: 206-208 ˚C. FT-IR (KBr): *ν*_max_ = 2952, 1644, 1590, 1524, 1488, 1373, 1241, 1131, 853 cm^−1^. ^1^H NMR (400 MHz, CDCl_3_): *δ* = 8.31 (d, *J* = 8.8 Hz, 2H, Ar-H), 7.85 (d, *J* = 8.8 Hz, 2H, Ar-H), 7.57-7.56 (m, 1H, Ar-H), 7.43-7.37 (m, 2H, Ar-H), 7.22 (t, *J* = 8.0 Hz, 1H, Ar-H), 6.49 (s, 1H, -CH), 2.55 (ABq, *J* = 17.6 Hz, 2H, -CH_2_), 2.28 (ABq, *J* = 16.4 Hz, 2H, -CH_2_), 1.14 (s, 3H, -CH_3_), 1.08 (s, 3H, -CH_3_). ^13^C NMR (100 MHz, CDCl_3_): *δ* = 195.48, 164.52, 158.59, 150.90, 149.27, 143.31, 134.38, 131.80, 130.42, 130.33, 127.33, 126.11, 124.43, 122.95, 110.69, 60.35, 50.63, 45.27, 32.71, 29.14, 27.71. Anal. Calcd for C_23_H_19_BrN_4_O_3_S: C, 54.02; H, 3.75; N, 10.96; S, 6.27. Found: 54.10; H, 3.72; N, 10.90; S, 6.34.

**8,8-Dimethyl-2-(4-nitrophenyl)-5-phenyl-5,7,8,9-tetrahydro-6H-[1,3,4]thiadiazolo[2,3-b]quinazolin-6-one: (4o)**

Yield: (317.92mg, 73 %); Orange solid; Mp: 235-237 ˚C. FT-IR (KBr): *ν*_max_ = 2937, 1636, 1594, 1522, 1495, 1375, 1241, 1133, 851 cm^−1^. ^1^H NMR (400 MHz, CDCl_3_): *δ* = 8.30 (d, *J* = 9.2 Hz, 2H, Ar-H), 7.84 (d, *J* = 9.2 Hz, 2H, Ar-H), 7.45-7.43 (m, 2H, Ar-H), 7.37-7.31 (m, 2H, Ar-H), 7.31-7.30 (m, 1H, Ar-H), 6.54 (s, 1H, -CH), 2.58 (d, *J* = 17.6 Hz, 1H, -CH_2_), 2.52 (d, *J* = 18.0 Hz, 1H, -CH_2_), 2.28 (ABq, *J* = 16.4 Hz, 2H, -CH_2_), 1.13 (s, 3H, -CH_3_), 1.07 (s, 3H, -CH_3_). ^13^C NMR (100 MHz, CDCl_3_): *δ* = 195.55, 164.58, 158.25, 150.51, 149.18, 141.30, 134.56, 128.76, 128.62, 127.28, 127.26, 124.37, 111.27, 60.79, 50.70, 45.28, 32.67, 29.19, 27.66. Anal. Calcd for C_23_H_20_N_4_O_3_S: C, 63.87; H, 4.66; N, 12.95; S, 7.41. Found: C, 63.97; H, 4.69; N, 13.01; S, 7.34.

**5-(4-(Dimethylamino)phenyl)-2-(4-nitrophenyl)-5,7,8,9-tetrahydro-6H-[1,3,4]thiadiazolo [2,3-b]quinazolin-6-one: (4p)**

Yield: (353.53 mg, 79 %); Orange solid; Mp: 263-265 ˚C. FT-IR (KBr): *ν*_max_ = 2940, 1644, 1609, 1523, 1487, 1372, 1342, 1242, 1150, 1129, 854 cm^−1^. ^1^H NMR (400 MHz, CDCl_3_): *δ* = 8.29 (d, *J* = 8.8 Hz, 2H, Ar-H), 7.85 (d, *J* = 9.2 Hz, 2H, Ar-H), 7.31 (s, 2H, Ar-H), 6.66 (d, *J* = 8.8 Hz, 2H, Ar-H), 6.49 (s, 1H, -CH), 2.93 (s, 6H, -CH_3_), 2.76-2.59 (m, 2H, -CH_2_), 2.47-2.39 (m, 2H, -CH_2_), 2.14-2.04 (m, 2H, -CH_2_). ^13^C NMR (100 MHz, CDCl_3_): *δ* = 195.88, 164.03, 159.73, 150.51, 150.26, 149.06, 134.77, 129.28, 128.28, 127.22, 124.33, 112.55, 112.06, 60.08, 40.35, 37.13, 31.48, 21.52. Anal. Calcd for C_23_H_21_N_5_O_3_S: C, 61.73; H, 4.73; N, 15.65; S, 7.16. Found: C, 61.64; H, 4.71; N, 15.60; S, 7.23

**5-([1,1'-Biphenyl]-4-yl)-8,8-dimethyl-2-phenyl-5,7,8,9-tetrahydro-6H-[1,3,4]thiadiazolo[2,3-b]quinazolin-6-one: (4q)**

Yield: (370.88 mg, 80 %); Yellow solid; Mp: 243-245 ˚C. FT-IR (KBr): *ν*_max_ = 2956, 1638, 1590, 1509, 1374, 1242, 1131, 757, 690 cm^−1^. ^1^H NMR (400 MHz, CDCl_3_): *δ* = 7.71-7.68 (m, 2H, Ar-H), 7.57-7.51 (m, 6H, Ar-H), 7.50-7.46 (m, 2H, Ar-H), 7.44-7.40 (m, 3H, Ar-H), 7.36-7.31 (m, 1H, Ar-H), 6.58 (s, 1H, -CH), 2.59 (d, *J* = 17.2 Hz, 1H), 2.53 (d, *J* = 17.6 Hz, 1H), 2.29 (ABq, *J* = 16.4 Hz, 2H, -CH_2_), 1.15 (s, 3H, -CH_3_), 1.09 (s, 3H, -CH_3_). ^13^C NMR (100 MHz, CDCl_3_): *δ* = 195.05, 164.62, 158.42, 153.07, 140.71, 140.20, 140.12, 131.08, 128.63, 128.49, 128.20, 127.23, 126.93, 126.82, 126.63, 126.03, 110.17, 59.85, 50.26, 44.90, 32.18, 28.79, 27.19. Anal. Calcd for C_29_H_25_N_3_OS: C, 75.13; H, 5.44; N, 9.06; S, 6.92. Found: C, 75.23; H, 5.41; N, 9.12; S, 6.84.

**5-([1,1'-Biphenyl]-4-yl)-2-phenyl-5,7,8,9-tetrahydro-6H-[1,3,4]thiadiazolo [2,3-b]quinazolin-6-one: (4r)**

Yield: (376.45 mg, 83 %); Yellow solid; Mp: 228-230 ˚C. FT-IR (KBr): *ν*_max_ = 2938, 1636, 1584, 1499, 1375, 1240, 1148, 1110, 760 cm^−1^. ^1^H NMR (400 MHz, CDCl_3_): *δ* = 7.70-7.68 (m, 2H, Ar-H), 7.56-7.49 (m, 7H, Ar-H), 7.48-7.40 (m, 4H, Ar-H), 7.35-7.35 (m, 1H, Ar-H), 6.62 (s, 1H, -CH), 2.77-2.60 (m, 2H, -CH_2_), 2.51-2.37 (m, 2H, -CH_2_), 2.12-2.02 (m, 2H, -CH_2_). ^13^C NMR (100 MHz, CDCl_3_): *δ* = 195.85, 164.92, 160.80, 153.64, 141.23, 140.71, 140.52, 131.62, 129.15, 128.96, 128.72, 127.74, 127.45, 127.33, 127.15, 126.54, 111.66, 60.05, 37.13, 31.60, 21.51. Anal. Calcd for C_27_H_21_N_3_OS: C, 74.46; H, 4.86; N, 9.65; S, 7.36. Found: C, 74.34; H, 4.89; N, 9.60; S, 7.43.

**2-(2-Methoxyphenyl)-8,8-dimethyl-5-(naphthalen-1-yl)-5,7,8,9-tetrahydro-6H-[1,3,4]thiadiazolo[2,3-b]quinazolin-6-one: (4s)**

Yield: (425.51 mg, 91 %); Yellow solid; Mp: 238-240 ˚C. FT-IR (KBr): *ν*_max_ = 2955, 1628, 1580, 1476, 1374, 1240, 1118, 782 cm^−1^. ^1^H NMR (400 MHz, CDCl_3_): *δ* = 8.68 (d, *J* = 7.6 Hz, 1H, Ar-H), 7.84 (d, *J* = 8.0 Hz, 1H, Ar-H), 7.79-7.76 (m, 2H, Ar-H), 7.68-7.61 (m, 2H, Ar-H), 7.53-7.50 (m, 1H, Ar-H), 7.43 (t, *J* = 7.6 Hz, 1H, Ar-H), 7.36-7.32 (m, 2H, Ar-H), 6.93-6.89 (m, 1H, Ar-H), 6.87 (s, 1H, -CH), 3.89 (s, 3H, -OCH_3_), 2.62 (ABq, *J* = 17.6 Hz, 2H, -CH_2_), 2.25 (ABq, *J* = 16.4 Hz, 2H, -CH_2_), 1.15 (s, 3H, -CH_3_), 1.10 (s, 3H, -CH_3_). ^13^C NMR (100 MHz, CDCl_3_): *δ* = 195.28, 165.52, 159.68, 156.22, 149.09, 138.59, 133.76, 132.41, 130.92, 129.18, 128.49, 127.48, 126.84, 126.42, 125.67, 125.35, 123.89, 121.20, 117.77, 111.43, 110.38, 55.73, 55.72, 50.68, 45.58, 32.60, 29.36, 27.83. Anal. Calcd for C_28_H_25_N_3_O_2_S: C, 71.92; H, 5.39; N, 8.99; S, 6.86. Found: C, 72.01; H, 5.42; N, 8.94; S, 6.81.

**5-(6-Chloro-4-oxo-4H-chromen-3-yl)-8,8-dimethyl-2-phenyl-5,7,8,9-tetrahydro-6H-[1,3,4]thiadiazolo[2,3-b]quinazolin-6-one: (4t)**

Yield: (416.48 mg, 85 %); Yellow solid; Mp: 260-262 ˚C. FT-IR (KBr): *ν*_max_ = 2951, 1641, 1581, 1502, 1377, 1242, 1132, 763 cm^−1^. ^1^H NMR (400 MHz, CDCl_3_): *δ* = 8.32 (s, 1H, C=CH), 8.12 (d, *J* = 2.4 Hz, 1H, Ar-H), 7.69-7.66 (m, 2H, Ar-H), 7.60-7.57 (m, 1H, Ar-H), 7.49-7.40 (m, 4H, Ar-H), 6.36 (s, 1H, -CH), 2.53 (ABq, *J* = 17.6 Hz, 2H, CH_2_), 2.24 (ABq, *J* = 16.4 Hz, 2H, CH_2_), 1.11 (s, 3H, CH_3_), 1.00 (s, 3H, CH_3_). ^13^C NMR (100 MHz, CDCl_3_): *δ* = 195.33, 174.57, 166.36, 160.87, 154.79, 153.95, 153.11, 133.32, 131.01, 130.69, 128.58, 128.48, 126.03, 125.22, 124.81, 120.92, 119.36, 106.23, 55.99, 50.35, 44.87, 32.11, 28.50, 27.10. Anal. Calcd for C_26_H_20_ClN_3_O_3_S: C, 63.74; H, 4.11; N, 8.58; S, 6.54. Found: 63.84; H, 4.07; N, 8.51; S, 6.62.

**5-Phenethyl-2-phenyl-5,7,8,9-tetrahydro-6H-[1,3,4]thiadiazolo[2,3-b]quinazolin-6-one: (4u)**

Yield: (333.25 mg, 86 %); Yellow solid; Mp: 128-130 ˚C. FT-IR (KBr): *ν*_max_ = 2936, 1629, 1574, 1465, 1380, 1260, 1153, 1128, 760 cm^−1^. ^1^H NMR (400 MHz, CDCl_3_): *δ* = 7.73-7.71 (m, 2H, Ar-H), 7.56-7.48 (m, 3H, Ar-H), 7.23-7.19 (m, 2H, Ar-H), 7.10-7.15 (m, 3H, Ar-H), 5.78 (t, *J* = 4.0 Hz, 1H, -CH), 2.73-2.81 (m, 1H, -CH_2_), 2.64-2.36 (m, 5H, -CH_2_), 2.34-2.27 (m, 2H, -CH_2_), 2.07-1.90 (m, 2H, -CH_2_). ^13^C NMR (100 MHz, CDCl_3_): *δ* = 196.19, 165.85, 162.36, 153.28, 141.09, 131.56, 129.23, 129.04, 128.26, 126.43, 125.80, 109.94, 57.23, 37.18, 35.60, 31.59, 30.24, 21.51. Anal. Calcd for C_23_H_21_N_3_OS: C, 71.29; H, 5.46; N, 10.84; S, 8.27. Found: C, 71.38; H, 5.44; N, 10.90; S, 8.20.

**8,8-Dimethyl-2-(4-nitrophenyl)-5-phenethyl-5,7,8,9-tetrahydro-6H-[1,3,4]thiadiazolo[2,3-b]quinazolin-6-one: (4v)**

Yield: (345.41 mg, 75 %); Orange solid; Mp: 166-168 ˚C. FT-IR (KBr): *ν*_max_ = 2958, 2922, 1636, 1590, 1522, 1484, 1386, 1342, 1252, 1132, 853 cm^−1^. ^1^H NMR (400 MHz, CDCl_3_): *δ* = 8.37 (d, *J* = 8.8 Hz, 2H, Ar-H), 7.89 (d, *J* = 8.8 Hz, 2H, Ar-H), 7.23-7.14 (m, 2H, Ar-H), 7.11 (d, *J* = 6.8 Hz, 3H, Ar-H), 5.81 (s, 1H, -CH), 2.84-2.78 (m, 1H, -CH_2_), 2.62-2.55 (m, 1H, -CH_2_), 2.49 (d, *J* = 4.8 Hz, 2H, -CH_2_), 2.38 (s, 2H, -CH_2_), 2.31 (s, 2H, -CH_2_), 1.18 (s, 3H, -CH_3_), 1.13 (s, 3H, -CH_3_). ^13^C NMR (100 MHz, CDCl_3_): *δ* = 195.95, 149.26, 140.65, 134.46, 128.35, 128.21, 127.21, 125.94, 124.53, 109.41, 57.77, 50.83, 45.12, 35.45, 32.49, 30.33, 29.11, 27.79. Anal. Calcd for C_25_H_24_N_4_O_3_S: C, 65.20; H, 5.25; N, 12.17; S, 6.96. Found: C, 65.10; H, 5.28; N, 12.23; S, 7.04.

**5-(4-Chlorophenyl)-8,8-dimethyl-2-(2-oxo-2H-chromen-3-yl)-5,7,8,9-tetrahydro-6H-[1,3,4]thiadiazolo[2,3-b]quinazolin-6-one: (6a)**

Yield: (347.88 mg, 71 %); Orange solid; Mp: 211-213 ˚C. FT-IR (KBr): *ν*_max_ = 2953, 1711, 1633, 1608, 1585, 1476, 1374, 1249, 1122, 759 cm^−1^. ^1^H NMR (400 MHz, CDCl_3_): *δ* = 8.55 (s, 1H, -C=C-H), 7.71-7.67 (m, 2H, Ar-H), 7.45-7.39 (m, 2H, Ar-H), 7.37 (d, *J* = 8.4 Hz, 2H, Ar-H), 7.31 (d, *J* = 8.4 Hz, 2H, Ar-H), 6.53 (s, 1H, -CH), 2.58 (s, 2H, -CH_2_), 2.26 (ABq, *J* = 16.4 Hz, 2H, -CH_2_), 1.13 (s, 3H, -CH_3_), 1.04 (s, 3H, -CH_3_). ^13^C NMR (100 MHz, CDCl_3_): *δ* = 195.29, 159.41, 153.86, 153.85, 141.12, 139.94, 134.44, 134.26, 130.07, 129.56, 129.55, 128.99, 128.66, 125.69, 118.22, 117.06, 116.14, 110.11, 60.40, 50.65, 44.75, 32.70, 29.25, 27.52. Anal. Calcd for C_26_H_20_ClN_3_O_3_S: C, 63.74; H, 4.11; N, 8.58; S, 6.54. Found: C, 63.65; H, 4.09; N, 8.64; S, 6.60.

**5-(3-Methoxyphenyl)-8,8-dimethyl-2-(2-oxo-2H-chromen-3-yl)-5,7,8,9-tetrahydro-6H-[1,3,4]thiadiazolo[2,3-b]quinazolin-6-one: (6b)**

Yield: (388.45 mg, 80 %); Orange solid; Mp: 187-189 ˚C. FT-IR (KBr): *ν*_max_ = 2956, 1720, 1633, 1607, 1482, 1375, 1247, 1128, 759 cm^−1^. ^1^H NMR (400 MHz, CDCl_3_): *δ* = 8.56 (s, 1H, -C=C-H ), 7.70-7.66 (m, 2H, Ar-H), 7.45-7.39 (m, 2H, Ar-H), 7.26 (t, *J* = 8.0 Hz, 1H, Ar-H), 7.03-6.99 (m, 2H, , Ar-H), 6.82 (dd, *J* = 8.4 Hz, 2.4 Hz, 1H), 6.53 (s, 1H, -CH), 3.81 (s, 3H, -OCH_3_), 2.54 (ABq, *J* = 17.2 Hz, 2H, -CH_2_), 2.27 (ABq, *J* = 16.4 Hz, 2H, -CH_2_), 1.13 (s, 3H, -CH_3_), 1.07 (s, 3H, -CH_3_).

^13^C NMR (100 MHz, CDCl_3_): *δ* = 195.51, 165.84, 159.76, 159.43, 159.09, 153.77, 146.88, 143.26, 140.75, 133.96, 129.71, 129.47, 125.59, 119.53, 118.30, 116.97, 116.46, 113.72, 113.08, 110.30, 60.63, 55.26, 50.69, 45.30, 32.62, 29.33, 27.53. Anal. Calcd for C_27_H_23_N_3_O_4_S: C, 66.79; H, 4.77; N, 8.65; S, 6.60. Found: C, 66.70; H, 4.75; N, 8.60; S, 6.67.

**5-(4-(Tert-butyl)phenyl)-2-(2-oxo-2H-chromen-3-yl)-5,7,8,9-tetrahydro-6H-[1,3,4]thiadiazolo[2,3-b]quinazolin-6-one (6c)**

Yield: (396.54 mg, 82 %); Orange solid; Mp: 196-198 ˚C. FT-IR (KBr): *ν*_max_ = 2956, 1721, 1638, 1580, 1606, 1488, 1375, 1251, 1122, 754. ^1^H NMR (400 MHz, CDCl_3_): *δ* = 8.57 (s, 1H, -C=C-H), 7.69-7.64 (m, 2H, Ar-H), 7.43-7.38 (m, 2H, Ar-H), 7.33 (s, 4H, Ar-H), 6.58 (s, 1H, -CH), 2.76-2.57 (m, 2H, -CH_2_), 2.49-2.34 (m, 2H, -CH_2_), 2.09-1.99 (m, 2H, -CH_2_), 1.28 (s, 9H, -CH_3_). ^13^C NMR (100 MHz, CDCl_3_): *δ* = 195.79, 159.43, 153.79, 153.78, 151.11, 140.66, 140.65, 138.76, 133.89, 133.88, 129.44, 126.73, 125.60, 125.55, 118.37, 116.97, 116.62, 111.57, 59.94, 37.13, 34.55, 31.57, 31.27, 21.46. Anal. Calcd for C_28_H_25_N_3_O_3_S: C, 69.54; H, 5.21; N, 8.69; S, 6.63. Found: 69.65; H, 5.24; N, 8.65; S, 6.57.

**5-(4-Nitrophenyl)-2-(2-oxo-2H-chromen-3-yl)-5,7,8,9-tetrahydro-6H-[1,3,4]thiadiazolo[2,3-b]quinazolin-6-one (6d)**

Yield: (401.61 mg, 85 %); Orange solid; Mp: 157-159 ˚C. FT-IR (KBr): *ν*_max_ = 2926, 1721, 1635, 1607, 1566, 1479, 1376, 1249, 1122, 760 cm^-1^. ^1^H NMR (400 MHz, CDCl_3_): *δ* = 8.56 (s, 1H, -C=C-H), 8.22 (d, *J* = 8.4 Hz, 2H, Ar-H), 7.73-7.68 (m, 2H, Ar-H), 7.61 (d, *J* = 8.8 Hz, 2H, Ar-H), 7.46-7.40 (m, 2H, Ar-H), 6.70 (s, 1H, -CH), 2.84-2.69 (m, 2H, -CH_2_), 2.45-2.42 (m, 2H, -CH_2_), 2.14-1.98 (m, 2H, -CH_2_). ^13^C NMR (100 MHz, CDCl_3_): *δ* = 195.19, 159.37, 154.01, 152.98, 150.34, 149.60, 148.01, 141.88, 141.85, 134.79, 129.74, 128.44, 125.86, 124.22, 118.05, 117.19, 115.54, 110.63, 60.62, 36.80, 29.70, 21.13. Anal. Calcd for C_24_H_16_N_4_O_5_S: C, 61.01; H, 3.41; N, 11.86; S, 6.79. Found: C, 61.09; H, 3.43; N, 11.91; S, 6.72.

**4-(8,8-Dimethyl-6-oxo-2-phenyl-6,7,8,9-tetrahydro-5H-[1,3,4]thiadiazolo[2,3-b]quinazolin-5-yl)benzaldehyde: (8a)**

Yield: (290.86 mg, 70 %); Yellow solid; Mp: 190-192 ˚C. FT-IR (KBr): *ν*_max_ = 2958, 1695, 1634, 1583, 1482, 1375, 1240, 1134, 764 cm^−1^. ^1^H NMR (400 MHz, CDCl_3_): *δ* = 9.99 (s, 1H, -CHO), 7.87 (d, *J* = 8.4 Hz, 2H, Ar-H), 7.67 (d, *J* = 6.8 Hz, 2H, Ar-H), 7.62 (d, *J* = 8.0 Hz, 2H, Ar-H), 7.55-7.43 (m, 3H, Ar-H), 6.60 (s, 1H, -CH), 2.59-2.50 (m, 2H, -CH_2_), 2.31 (d, *J* = 16.4 Hz, 1H, -CH_2_), 2.23 (d, *J* = 16.0 Hz, 1H, -CH_2_), 1.14 (s, 3H, -CH_3_), 1.04 (s, 3H, -CH_3_). ^13^C NMR (100 MHz, CDCl_3_): *δ* = 195.44, 191.77, 165.28, 159.24, 154.03, 147.71, 136.17, 131.81, 130.20, 129.20, 128.72, 127.99, 126.53, 110.19, 60.48, 50.64, 45.36, 32.66, 29.24, 27.56. Anal. Calcd for C_24_H_21_N_3_O_2_S: C, 69.38; H, 5.09; N, 10.11; S, 7.72. Found: C, 69.47; H, 5.06; N, 10.17; S, 7.64.

**3-(2-(2-Methoxyphenyl)-8,8-dimethyl-6-oxo-6,7,8,9-tetrahydro-5H-[1,3,4]thiadiazolo[2,3-b]quinazolin-5-yl)benzaldehyde: (8b)**

Yield: (334.16 mg, 75 %); Yellow solid; Mp: 184-186 ˚C. FT-IR (KBr): *ν*_max_ = 2958, 1693, 1632, 1580, 1468, 1376, 1245, 1118, 764 cm^−1^. ^1^H NMR (400 MHz, CDCl_3_): *δ* = 10.00 (s, 1H, -CHO), 7.97 (dd,^1^ *J* = 8.0 Hz, ^2^ *J* = 1.6 Hz, 1H, Ar-H), 7.91 (s, 1H, Ar-H ), 7.79 (d, *J* = 7.6 Hz, 1H, Ar-H), 7.73 (d, *J* = 8.0 Hz, 1H, Ar-H), 7.50 (t, *J* = 7.6 Hz, 1H, Ar-H), 7.47-7.42 (m, 1H, Ar-H), 7.05-6.98 (m, 2H, Ar-H), 6.63 (s, 1H, -CH), 3.97 (s, 3H, -OCH_3_), 2.55 (ABq, *J* = 17.6 Hz, 2H, -CH_2_), 2.26 (ABq, *J* = 16.4 Hz, 2H, -CH_2_), 1.13 (s, 3H, -CH_3_), 1.05 (s, 3H, -CH_3_). ^13^C NMR (100 MHz, CDCl_3_): *δ* = 195.29, 192.15, 165.93, 160.00, 156.37, 149.93, 143.14, 136.66, 133.47, 132.78, 129.32, 129.14, 128.92, 127.54, 121.35, 117.67, 111.57, 109.36, 60.21, 55.84, 50.67, 45.49, 32.66, 29.28, 27.65. Anal. Calcd for C_25_H_23_N_3_O_3_S: C, 67.40; H, 5.20; N, 9.43; S, 7.20. Found: C, 67.31; H, 5.23; N, 9.38; S, 7.28.

**2-(4-(2-(2-(2-Methoxyphenyl)-8,8-dimethyl-6-oxo-6,7,8,9-tetrahydro-5H-[1,3,4]thiadiazolo[2,3-b]quinazolin-5-yl)phenoxy)butoxy)benzaldehyde: (8c)**

Yield: (396.33 mg, 65 %); Yellow solid; Mp: 178-180 ˚C. FT-IR (KBr): *ν*_max_ = 2949, 2888, 1686, 1632, 1594, 1483, 1375, 1241, 1118, 754 cm^−1^. ^1^H NMR (400 MHz, CDCl_3_): *δ* = 10.57 (s, 1H, -CHO), 7.97 (dd, ^1^*J* = 8.0 Hz, ^2^*J* = 1.6 Hz, 1H, Ar-H), 7.88 (dd, ^1^*J* = 7.8 Hz, ^2^*J* = 1.6 Hz, 1H, Ar-H), 7.60-7.53 (m, 2H, Ar-H), 7.48-7.43 (m, 1H, Ar-H), 7.29-7.25 (m, 1H, Ar-H), 7.08-6.97 (m, 5H, Ar-H), 6.91 (d, *J* = 8.4 Hz, 1H, Ar-H), 6.83 (s, 1H, -CH), 4.32-4.27 (m, 2H, -CH_2_), 4.19-4.11 (m, 2H, -CH_2_), 3.99 (s, 3H, -OCH_3_), 2.59-2.49 (m, 2H, -CH_2_), 2.33-2.24 (m, 2H, -CH_2_), 2.21-2.13 (m, 4H, -CH_2_), 1.16 (s, 3H, -CH_3_), 1.07 (s, 3H, -CH_3_). ^13^C NMR (100 MHz, CDCl_3_): *δ* = 195.02, 189.71, 165.98, 161.42, 159.62, 156.76, 156.25, 148.48, 135.90, 132.36, 130.63, 129.81, 129.54, 128.19, 127.50, 124.82, 121.13, 120.51, 120.45, 117.98, 112.52, 111.79, 111.54, 109.24, 68.20, 67.88, 57.72, 55.78, 50.76, 45.64, 32.49, 29.55, 27.29, 26.34, 26.02. Anal. Calcd for C_35_H_35_N_3_O_5_S: C, 68.94; H, 5.79; N, 6.89; S, 5.26. Found: C, 68.83; H, 5.75; N, 6.96; S, 5.34.

**(6-Oxo-2-phenyl-6,7,8,9-tetrahydro-5H-[1,3,4]thiadiazolo[2,3-b]quinazolin-5-yl)benzaldehyde (8d)**

Yield: (293.08 mg, 73 %); Yellow solid; Mp: 147-149 ˚C. FT-IR (KBr): *ν*_max_ = 2937, 1698, 1636, 1572, 1480, 1375, 1238, 1121, 761 cm^−1^. ^1^H NMR (400 MHz, CDCl_3_): *δ* = 9.99 (s, 1H, -CHO), 7.87 (d, *J* = 8.4 Hz, 2H, Ar-H), 7.60 (d, *J* = 8.0 Hz, 2H, Ar-H), 7.42 (d, *J* = 8.0 Hz, 1H, Ar-H), 7.39 -7.35 (m, 1H, Ar-H), 7.32-7.29 (m, 1H, Ar-H), 7.26 (d, *J* = 7.6 Hz, 1H, Ar-H), 6.64 (s, 1H, -CH), 2.77-2.59 (m, 2H, -CH_2_), 2.44-2.40 (m, 2H, -CH_2_), 2.39 (s, 3H, -CH_3_), 2.12-1.99 (m, 2H, -CH_2_). ^13^C NMR (100 MHz, CDCl_3_): *δ* = 195.69, 191.78, 165.11, 161.37, 147.59, 137.38, 136.17, 132.02, 131.07, 130.16, 130.15, 129.52, 128.07, 128.02, 126.47, 111.01, 60.21, 36.99, 31.60, 21.57, 21.45. Anal. Calcd for C_23_H_19_N_3_O_2_S: C, 68.81; H, 4.77; N, 10.47; S, 7.99. Found: C, 68.90; H, 4.75; N, 10.54; S, 7.91.

**5,5'-(1,4-Phenylene)bis(8,8-dimethyl-2-phenyl-5,7,8,9-tetrahydro-6H-[1,3,4]thiadiazolo[2,3-b]quinazolin-6-one): (9a)**

Yield: (432.07 mg, 62 %); Yellow solid; Mp: 290-292 ˚C. FT-IR (KBr): *ν*_max_ = 2951, 1640, 1584, 1490, 1375, 1241, 1133, 761 cm^−1^. ^1^H NMR (400 MHz, CDCl_3_): *δ* = 7.65-7.63 (m, 4H, Ar-H), 7.47-7.39 (m, 6H, Ar-H), 7.34 (d, *J* = 1.2 Hz, 4H, Ar-H), 6.50 (d, *J* = 9.6 Hz, 2H, -CH), 2.57-2.43 (m, 4H, -CH_2_), 2.28-2.16 (m, 4H, -CH_2_), 1.09 (d, *J* = 2.8 Hz, 6H, -CH_3_), 1.04 (s, 3H, -CH_3_), 1.00 (s, 3H, -CH_3_). ^13^C NMR (100 MHz, CDCl_3_): *δ* = 195.50, 195.49, 165.19, 165.02, 159.26, 159.06, 153.70, 153.62, 141.50, 141.49, 141.15, 131.53, 131.52, 129.09, 128.97, 128.94, 127.60, 127.51, 126.57, 126.54, 110.47, 110.16, 59.92, 59.75, 50.73, 50.69, 45.35, 45.34, 32.66, 29.13, 29.03, 28.03, 27.68. Anal. Calcd for C_40_H_36_N_6_O_2_S_2_: C, 68.94; H, 5.21; N, 12.06; S, 9.20. Found: C, 68.84; H, 5.24; N, 12.01; S, 9.27.

**5,5'-(1,3-Phenylene)bis(2-(2-methoxyphenyl)-8,8-dimethyl-5,7,8,9-tetrahydro-6H-[1,3,4]thiadiazolo[2,3-b]quinazolin-6-one): (9b)**

Yield: (492.01 mg, 65 %); Yellow solid; Mp: 173-175 ˚C. FT-IR (KBr): *ν*_max_ = 2953, 1637, 1576, 1469, 1374, 1249, 1118, 755 cm^−1^. ^1^H NMR (400 MHz, CDCl_3_): *δ* = 7.97 (dd, ^1^*J* = 8.0 Hz, ^2^*J* = 1.6 Hz, 1H, Ar-H), 7.88 (dd, ^1^*J* = 7.8 Hz, ^2^*J* = 1.6 Hz, 1H, Ar-H), 7.42-7.38 (m, 3H, Ar-H), 7.32 (d, *J* = 7.6 Hz, 2H, Ar-H), 7.27-7.22 (m, 1H, Ar-H), 7.00-6.91 (m, 4H, Ar-H), 6.53 (d, *J* = 10.0 Hz, 2H, -CH), 3.94 (d, *J* = 2.0 Hz, 6H, -OCH_3_), 2.55-2.42 (m, 4H, -CH_2_), 2.25-2.10 (m, 4H, -CH_2_), 1.07 (d, *J* = 2.8 Hz, 6H, -CH_3_), 0.99 (s, 3H, -CH_3_), 0.93 (s, 3H, -CH_3_). ^13^C NMR (100 MHz, CDCl_3_): *δ* = 194.59, 194.49, 155.80, 155.74, 141.77, 141.54, 131.98, 131.92, 128.46, 128.16, 128.10, 127.34, 127.28, 127.21, 126.81, 126.68, 126.52, 125.79, 125.28, 124.58, 120.86, 120.74, 117.40, 117.30, 110.94, 110.89, 109.10, 109.04, 59.60, 59.37, 55.31, 55.29, 50.23, 50.14, 44.75, 44.73, 32.01, 31.96, 28.73, 28.66, 27.30, 27.20. Anal. Calcd for C_42_H_40_N_6_O_4_S_2_: C, 66.64; H, 5.33; N, 11.10; S, 8.47. Found: C, 66.74; H, 5.31; N, 11.16; S, 8.40.

**5,5'-((Butane-1,4-diylbis(oxy))bis(2,1-phenylene))bis(2-(2-methoxyphenyl)-8,8-dimethyl-5,7,8,9-tetrahydro-6H-[1,3,4]thiadiazolo[2,3-b]quinazolin-6-one): (9c)**

Yield: (552.68 mg, 60 %); Yellow solid; Mp: 195-197 ˚C. FT-IR (KBr): *ν*_max_ = 2951, 1629, 1583, 1469, 1376, 1244, 1119, 754 cm^−1^. ^1^H NMR (400 MHz, CDCl_3_): *δ* = 7.85 (dd, ^1^*J* = 8.0 Hz, ^2^*J* = 1.6 Hz, 2H, Ar-H), 7.38 (dd, , ^1^*J* = 7.6 Hz, ^2^*J* = 1.6 Hz, 2H, Ar-H), 7.33-7.30 (m, 2H, Ar-H), 7.20-7.16 (m, 2H, Ar-H), 6.90-6.86 (m, 8H, Ar-H), 6.78 (s, 2H, -CH), 4.20-4.15 (m, 2H, -CH_2_), 4.12-4.08 (m, 2H, -CH_2_), 3.89 (s, 6H, -OCH_3_), 2.48 (s, 4H, -CH_2_), 2.28-2.14 (m, 8H, -CH_2_), 1.13 (s, 6H, -CH_3_), 1.05 (s, 6H, -CH_3_). ^13^C NMR (100 MHz, CDCl_3_): *δ* = 194.83, 165.64, 159.59, 156.60, 156.02, 148.29, 132.06, 130.18, 129.90, 129.50, 127.37, 120.98, 120.30, 117.83, 111.72, 111.27, 109.31, 68.19, 56.21, 55.67, 50.76, 45.63, 32.46, 29.70, 27.35, 26.51. Anal. Calcd for C_52_H_52_N_6_O_6_S_2_: C, 67.80; H, 5.69; N, 9.12; S, 6.96. Found: C, 67.89; H, 5.65; N, 9.06; S, 7.03.

**5,5'-(1,4-Phenylene)bis(2-phenyl-5,7,8,9-tetrahydro-6H-[1,3,4]thiadiazolo [2,3-b]quinazolin-6-one) (9d)**

Yield: (428.05 mg, 64 %); Yellow solid; Mp: 264-266 ˚C. FT-IR (KBr): *ν*_max_ = 2942, 1634, 1576, 1485, 1376, 1238, 1123, 758 cm^−1^. ^1^H NMR (400 MHz, CDCl_3_): *δ* = 7.42-7.38 (m, 2H, Ar-H), 7.37-7.31 (m, 6H, Ar-H), 7.27-7.23 (m, 3H, Ar-H), 7.19 (d, *J* = 7.6 Hz, 1H, Ar-H), 6.53 (s, 2H, -CH), 2.74-2.55 (m, 4H, -CH_2_), 2.46-2.39 (m, 4H, -CH_2_), 2.35 (s, 3H, -CH_3_), 2.30 (s, 3H, -CH_3_), 2.09-2.01 (m, 4H, -CH_2_). ^13^C NMR (100 MHz, CDCl_3_): *δ* = 195.76, 195.72, 164.99, 164.97, 161.16, 161.13, 153.64, 153.56, 141.32, 141.28, 137.42, 137.40, 131.92, 130.82, 130.81, 129.51, 129.45, 127.79, 127.76, 127.58, 127.58, 126.37, 126.37, 126.35, 111.20, 111.18, 59.72, 59.67, 37.08, 37.07, 31.59, 31.58, 21.51, 21.50, 21.47, 21.44. Anal. Calcd for C_38_H_32_N_6_O_2_S_2_: C, 68.24; H, 4.82; N, 12.57; S, 9.59. Found: C, 68.13; H, 4.80; N, 12.53; S, 9.67.

**5,5'-((Butane-1,4-diylbis(sulfanediyl))bis(4,1-phenylene))bis(1,3,4-thiadiazol-2-amine) (10)**

Yield: (256.38 mg, 80 %); Pale yellow solid; Mp: 262-264 ˚C. ^1^H NMR (400 MHz, DMSO-*d_6_*): *δ* = 7.31 (s, 4H, -NH_2_), 3.07 (s, 4H, -CH_2_), 1.74 (s, 4H, -CH_2_). ^13^C NMR (100 MHz, DMSO-*d_6_*): *δ* = 169.48, 149.98, 33.64, 27.87.

**2-((4-((5-Amino-1,3,4-thiadiazol-2-yl)thio)butyl)thio)-5-(2,4-dichlorophenyl)-8,8-dimethyl-5,7,8,9-tetrahydro-6H-[1,3,4]thiadiazolo[2,3-b]quinazolin-6-one (11a)**

Yield: (359.78 mg, 60 %); Yellow solid; Mp: 135-137 ˚C. FT-IR (KBr): *ν*_max_ = 3339, 1618, 1583, 1466, 1376, 1235, 1142, 857 cm^−1^. ^1^H NMR (400 MHz, CDCl_3_): 7.38 (d, *J* = 2.4 Hz, 1H, Ar-H), 7.36 (s, 1H, Ar-H), 7.23 (dd, *^1^J* = 8.4 Hz, *^2^J* = 2.0 Hz, 1H, Ar-H), 6.78 (s, 1H, -CH), 5.25 (s, 2H, -NH_2_), 3.19-3.00 (m, 4H, -CH_2_), 2.49 (q, *J* = 18.0 Hz, 2H, -CH_2_), 2.23 (ABq, *J* = 16.4 Hz, 2H, -CH_2_), 1.86-1.71 (m, 4H, -CH_2_), 1.11 (s, 3H, -CH_3_), 1.06 (s, 3H, -CH_3_). ^13^C NMR (100 MHz, CDCl_3_): *δ* = 195.46, 168.44, 159.96, 152.53, 152.47, 137.09, 134.82, 133.91, 131.51, 131.44, 129.78, 127.57, 109.36, 58.14, 50.56, 45.32, 33.89, 32.58, 32.55, 29.18, 28.26, 27.98, 27.68. Anal. Calcd for C_23_H_24_Cl_2_N_6_OS_4_: C, 46.07; H, 4.03; N, 14.02; S, 21.39. Found: C, 46.17; H, 4.06; N, 13.96; S, 21.47.

**2-((4-((5-Amino-1,3,4-thiadiazol-2-yl)thio)butyl)thio)-5-(3-bromophenyl)-8,8-dimethyl-5,7,8,9-tetrahydro-6H-[1,3,4]thiadiazolo[2,3-b]quinazolin-6-one (11b)**

Yield: (432.84 mg, 71 %); Yellow solid; Mp: 183-185 ˚C. FT-IR (KBr): *ν*_max_ = 3340, 2929, 1628, 1579, 1467, 1378, 1231, 1116, 762 cm^−1^. ^1^H NMR (400 MHz, CDCl_3_): *δ* = 7.48 (d, *J* = 1.6 Hz, 1H, Ar-H), 7.41 (d, *J* = 8.0 Hz, 1H, Ar-H), 7.30 (d, *J* = 8.0 Hz, 1H, Ar-H), 7.23-7.19 (m, 1H, Ar-H), 6.39 (s, 1H, -CH), 5.66 (s, 2H, -NH_2_), 3.21-3.02 (m, 4H, -CH_2_), 2.51 (ABq, *J* = 17.6 Hz, 2H, -CH_2_), 2.26 (ABq, *J* = 16.4 Hz, 2H, -CH_2_), 1.84-1.75 (m, 4H, -CH_2_), 1.12 (s, 3H, -CH_3_), 1.08 (s, 3H, -CH_3_). ^13^C NMR (100 MHz, CDCl_3_): *δ* = 195.66, 165.40, 159.62, 152.73, 152.68, 143.26, 131.64, 131.63, 130.42, 130.29, 126.11, 122.83, 109.84, 59.97, 50.61, 45.23, 33.86, 33.85, 32.68, 29.17, 28.23, 27.94, 27.72. Anal. Calcd for C_23_H_25_BrN_6_OS_4_: C, 45.31; H, 4.13; N, 13.79; S, 21.04. Found: C, 45.22; H, 4.11; N, 13.74; S, 21.11.

**2-((4-((5-Amino-1,3,4-thiadiazol-2-yl)thio)butyl)thio)-8,8-dimethyl-5-(3-nitrophenyl)-5,7,8,9-tetrahydro-6H-[1,3,4]thiadiazolo[2,3-b]quinazolin-6-one (11c)**

Yield: (437.56 mg, 76 %); Yellow solid; Mp: 186-188 ˚C. FT-IR (KBr): *ν*_max_ = 3354, 2933, 1616, 1572, 1485, 1378, 1241, 1118, 764 cm^−1^. ^1^H NMR (400 MHz, CDCl_3_): *δ* = 8.22-8.21 (m, 1H, Ar-H), 8.16-8.14 (m, 1H, Ar-H), 7.75 (d, *J* = 7.6 Hz, 1H, Ar-H), 7.54 (t, *J* = 8.0 Hz, 1H, Ar-H), 6.55 (s, 1H, -CH), 5.77 (s, 2H, -NH_2_), 3.16-3.03 (m, 4H, -CH_2_), 2.58 (d, *J* = 17.6 Hz, 1H, -CH_2_), 2.50 (d, *J* = 18.0 Hz, 1H, -CH_2_), 2.30 (d, *J* = 16.8 Hz, 1H, -CH_2_), 2.23 (d, *J* = 16.4 Hz, 1H, -CH_2_), 1.85-1.77 (m, 4H, -CH_2_), 1.13 (s, 3H, -CH_3_), 1.08 (s, 3H, -CH_3_). ^13^C NMR (100 MHz, CDCl_3_): *δ* = 195.63, 165.36, 159.90, 153.43, 148.52, 148.51, 142.99, 133.70, 133.70, 129.77, 123.52, 122.38, 109.40, 60.00, 50.57, 45.20, 33.67, 32.70, 32.67, 29.17, 28.25, 27.88, 27.71. Anal. Calcd for C_23_H_25_N_7_O_3_S_4_: C, 47.98; H, 4.38; N, 17.03; S, 22.27. Found: C, 47.88; H, 4.42; N, 17.09; S, 22.20.

**2,2'-(Butane-1,4-diylbis(sulfanediyl))bis(5-(2,4-dichlorophenyl)-8,8-dimethyl-5,7,8,9-tetrahydro-6H-[1,3,4]thiadiazolo[2,3-b]quinazolin-6-one) (12a)**

Yield: (500.91 mg, 57 %); Yellow solid; Mp: 203-205 ˚C. FT-IR (KBr): *ν*_max_ = 2953, 1636, 1585, 1469, 1374, 1240, 1138, 854 cm^−1^. ^1^H NMR (400 MHz, CDCl_3_): *δ* = 7.44-7.38 (m, 4H, Ar-H), 7.29-7.26 (m, 2H, Ar-H), 6.80 (d, *J* = 4.8 Hz, 2H, -CH), 3.11-3.01 (m, 2H, -CH_2_), 2.96-2.90 (m, 2H, -CH_2_), 2.56 (s, 4H, -CH_2_), 2.27 (ABq, *J* = 16.4 Hz, 4H, -CH_2_), 1.72-1.63 (m, 4H, -CH_2_), 1.16 (s, 6H, -CH_3_), 1.11 (s, 6H, -CH_3_). ^13^C NMR (100 MHz, CDCl_3_): *δ* = 194.82, 165.00, 159.11, 151.72, 136.71, 134.25, 133.39, 130.99, 129.18, 127.03, 108.93, 57.84, 50.03, 44.76, 32.07, 31.87, 28.65, 27.29, 27.17. Anal. Calcd for C_38_H_36_Cl_4_N_6_O_2_S_4_: C, 51.94; H, 4.13; N, 9.56; S, 14.59. Found: C, 51.85; H, 4.11; N, 9.61; S, 14.66.

**2,2'-(Butane-1,4-diylbis(sulfanediyl))bis(5-(3-bromophenyl)-8,8-dimethyl-5,7,8,9-tetrahydro-6H-[1,3,4]thiadiazolo[2,3-b]quinazolin-6-one) (12b)**

Yield: (611.19 mg, 68 %); Yellow solid; Mp: 140-142 ˚C. FT-IR (KBr): *ν*_max_ = 2953, 1636, 1586, 1470, 1374, 1239, 1115, 765 cm^−1^. ^1^H NMR (400 MHz, CDCl_3_): *δ* = 7.51-7.49 (m, 2H, Ar-H), 7.40-7.36 (m, 2H, Ar-H), 7.32-7.29 (m, 2H, Ar-H), 7.21-7.17 (m, 2H, Ar-H), 6.35 (d, *J* = 5.6 Hz, 2H, -CH), 3.12-3.04 (m, 2H, -CH_2_), 2.94-2.86 (m, 2H, -CH_2_), 2.52 (ABq, *J* = 17.6 Hz, 4H, -CH_2_), 2.26 (ABq, *J* = 16.4 Hz, 4H, -CH_2_), 1.69-1.62 (m, 4H, -CH_2_), 1.12 (s, 6H, -CH_3_), 1.07 (s, 6H, -CH_3_). ^13^C NMR (100 MHz, CDCl_3_): *δ* = 195.31, 165.02, 158.51, 143.33, 131.61, 130.50, 130.29, 125.99, 125.98, 122.79, 110.02, 60.18, 50.60, 44.93, 32.68, 32.51, 29.11, 27.72, 27.57. Anal. Calcd for C_38_H_38_Br_2_N_6_O_2_S_4_: C, 50.78; H, 4.26; N, 9.35; S, 14.27. Found: C, 50.89; H, 4.29; N, 9.30; S, 14.20.

**2,2'-(Butane-1,4-diylbis(sulfanediyl))bis(8,8-dimethyl-5-(3-nitrophenyl)-5,7,8,9-tetrahydro-6H-[1,3,4]thiadiazolo[2,3-b]quinazolin-6-one) (12c)**

Yield: (581.71 mg, 70 %); Yellow solid; Mp: 161-163 ˚C. FT-IR (KBr): *ν*_max_ = 2952, 1632, 1579, 1469, 1377, 1243, 1119, 718 cm^−1^. ^1^H NMR (400 MHz, CDCl_3_): *δ* = 8.23-8.21 (m, 2H, Ar-H), 8.15-8.12 (m, 2H, Ar-H), 7.72 (dd, ^1^*J* = 7.6 Hz, ^2^*J* = 0.8 Hz, 2H, Ar-H), 7.54-7.50 (m, 2H, Ar-H), 6.51 (d, *J* = 6.4 Hz, 2H, -CH), 3.06-2.94 (m, 4H, -CH_2_), 2.54 (ABq, *J* = 17.7 Hz, 4H, -CH_2_), 2.26 (ABq, *J* = 16.4 Hz, 4H, -CH_2_), 1.69 (s, 4H, -CH_2_), 1.13 (s, 6H, -CH_3_), 1.07 (s, 6H, -CH_3_). ^13^C NMR (100 MHz, CDCl_3_): *δ* = 195.34, 165.01, 148.76, 148.44, 142.99, 133.44, 129.74, 129.73, 123.46, 122.47, 109.55, 60.12, 50.55, 44.99, 32.70, 32.53, 29.09, 27.73, 27.52. Anal. Calcd for C_38_H_38_N_8_O_6_S_4_: C, 54.92; H, 4.61; N, 13.48; S, 15.43. Found: C, 54.83; H, 4.64; N, 13.42; S, 15.51.

# **^1^H and ^13^C NMR spectra of the products**

**
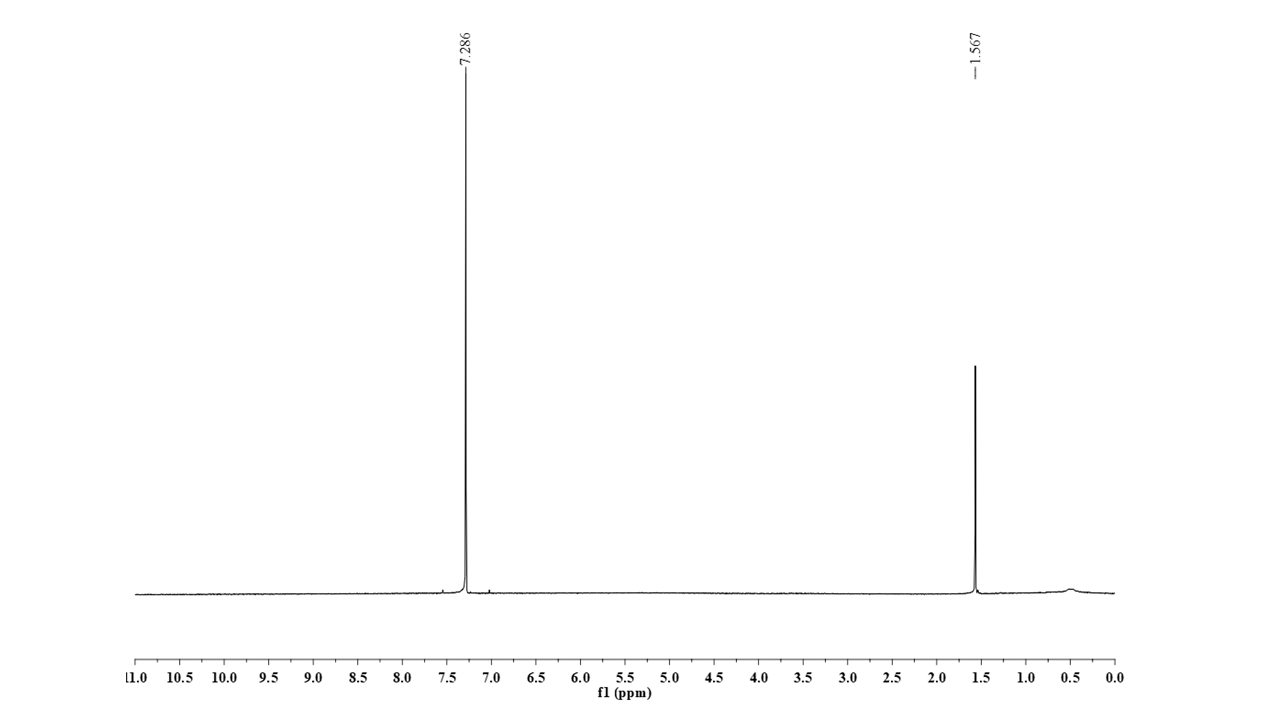
**

**Figure S1. ^1^H NMR of CDCl_3_ at 400 MHz**

**
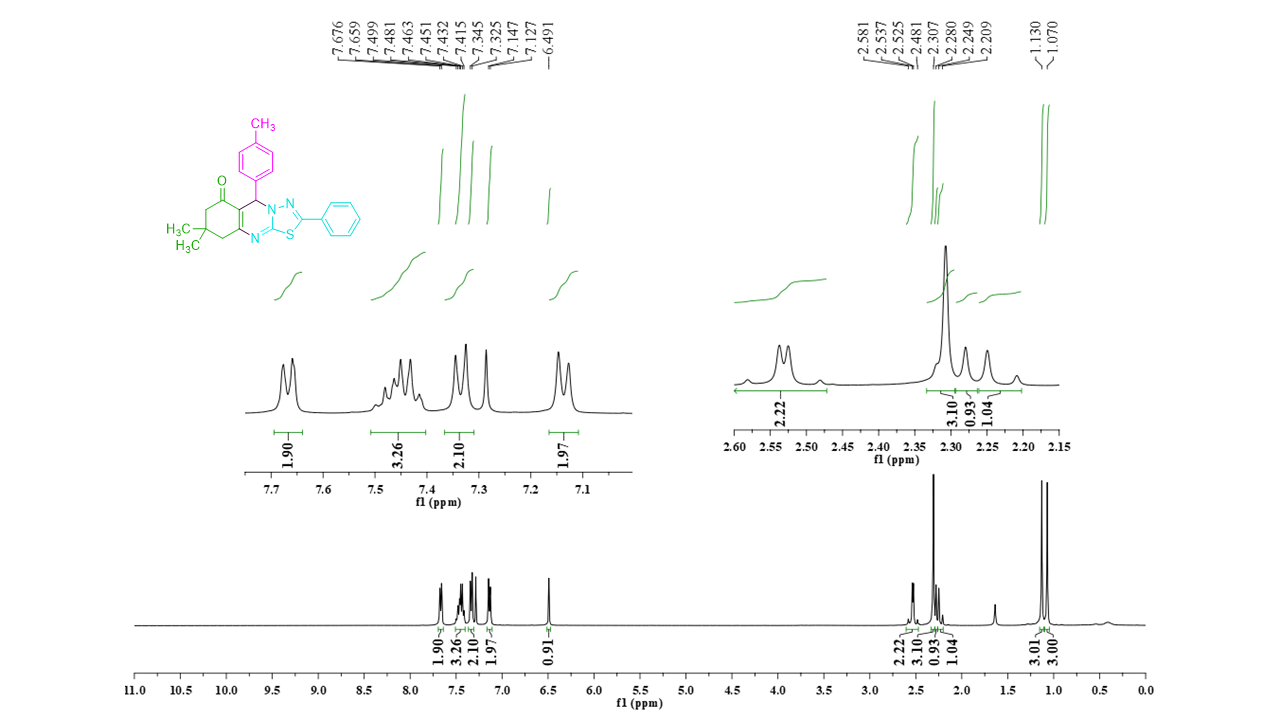
**

**Figure S2. ^1^H NMR (400 MHz, CDCl_3_) of 4a**

**
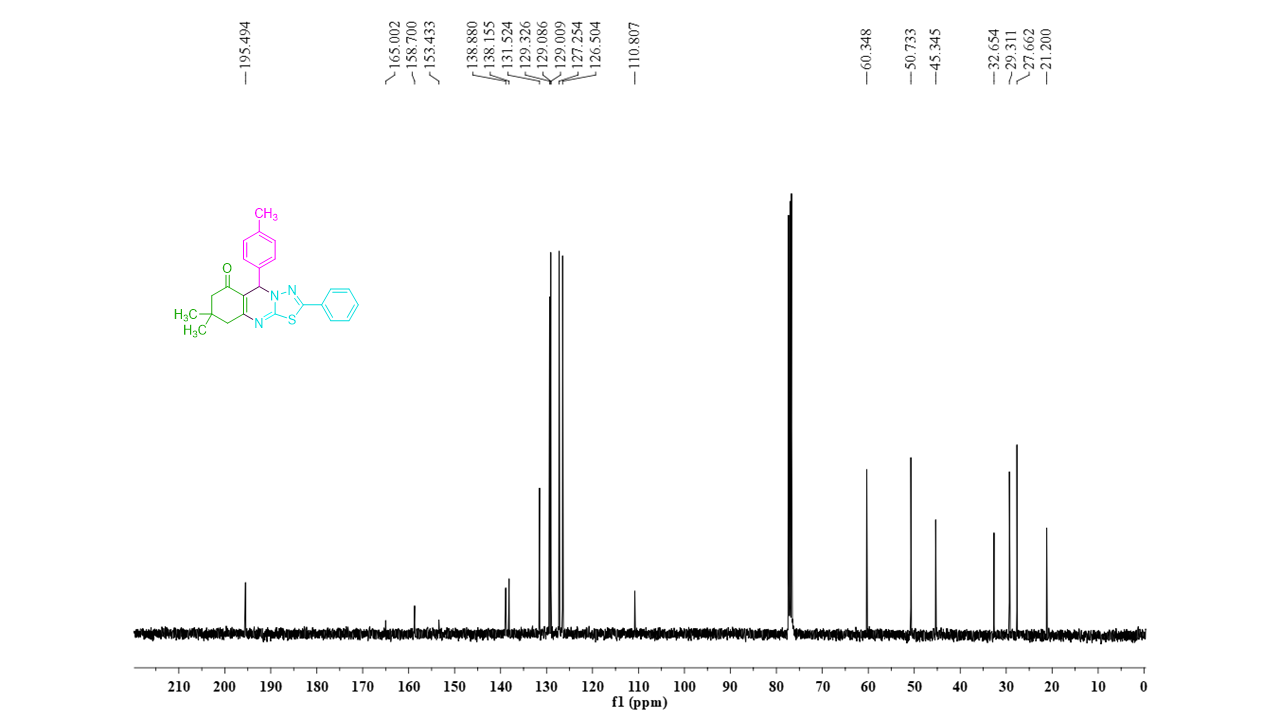
**

**Figure S3. ^13^C NMR (100 MHz, CDCl_3_) of 4a**

**
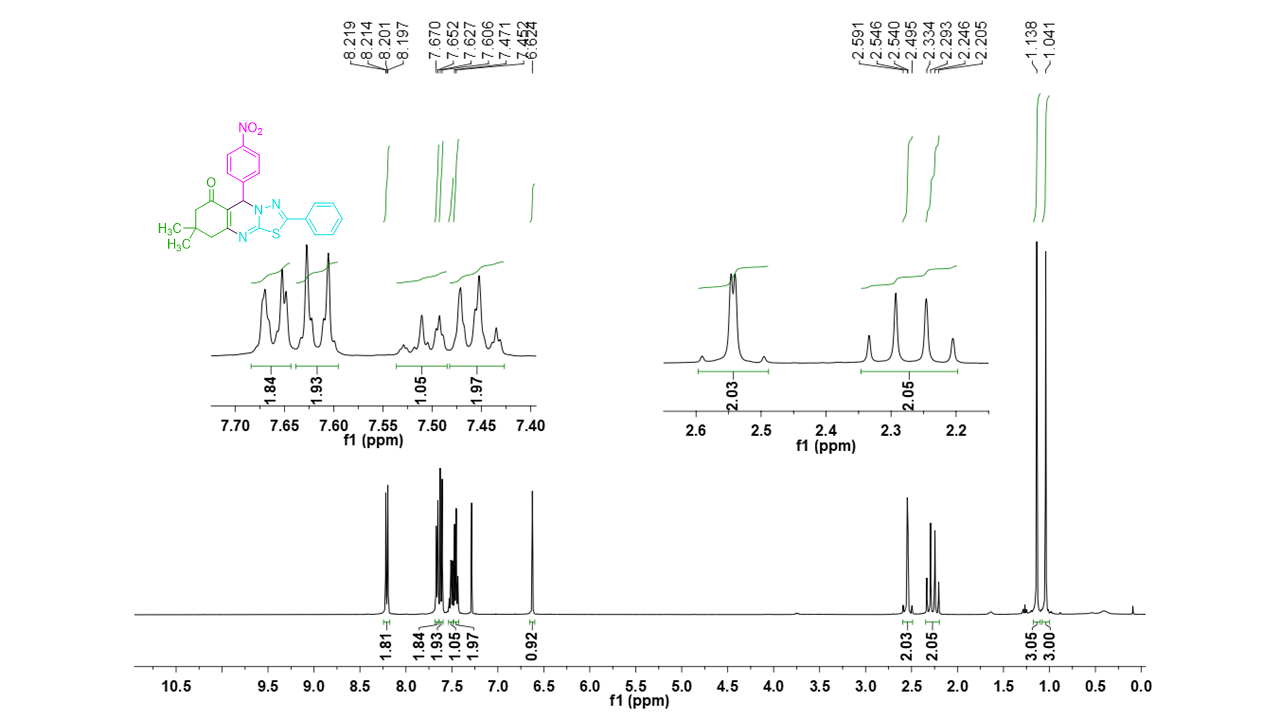
**

**Figure S4. ^1^H NMR (400 MHz, CDCl_3_) of 4b**

**
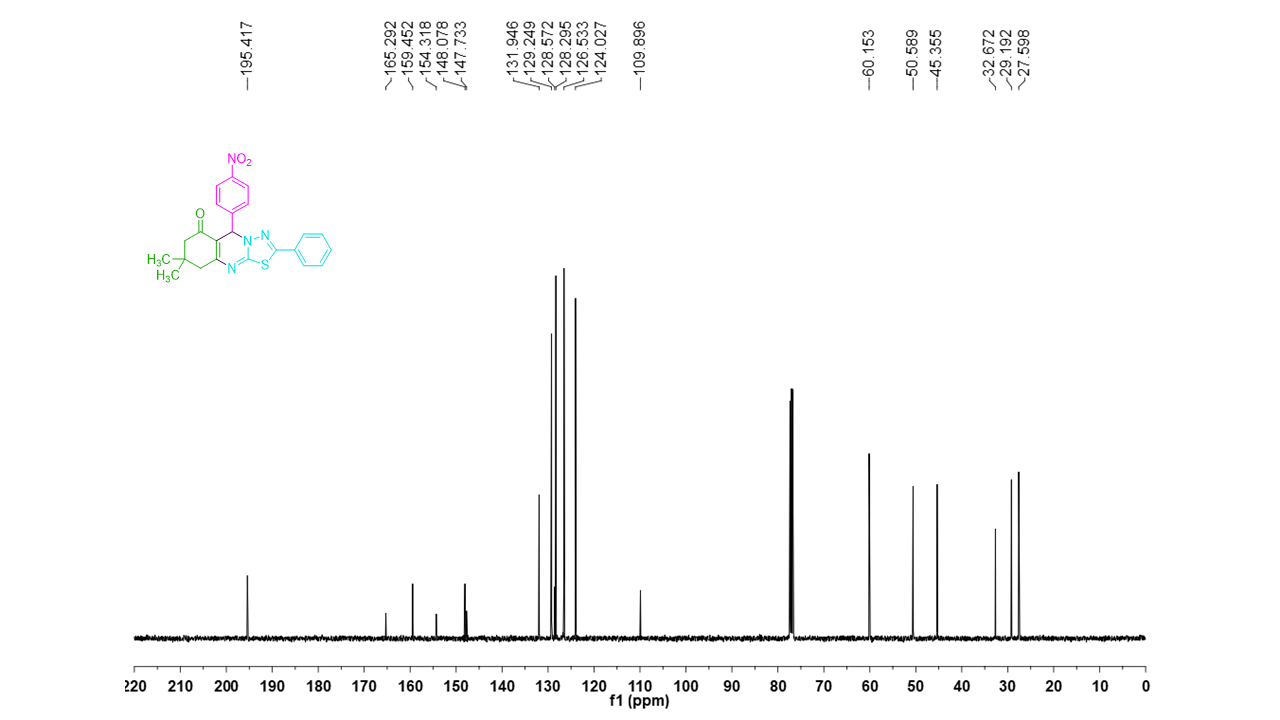
**

**Figure S5. ^13^C NMR (100 MHz, CDCl_3_) of 4b**

**
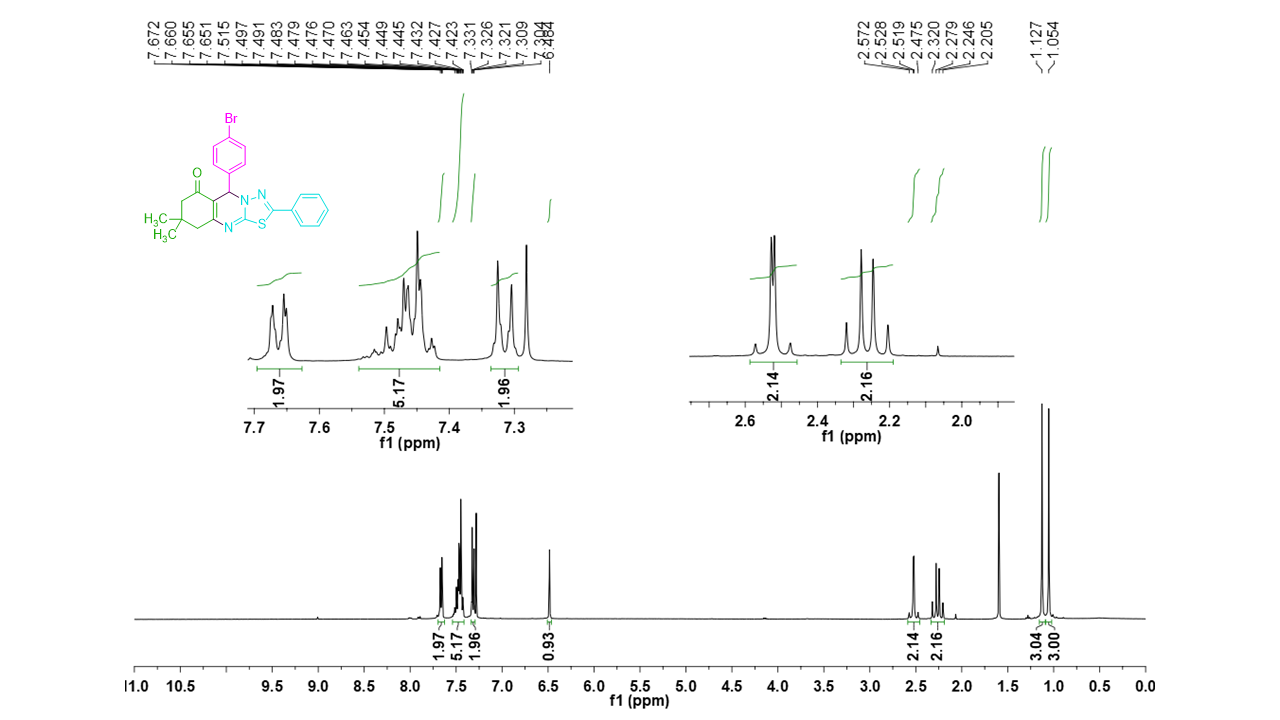
**

**Figure S6. ^1^H NMR (400 MHz, CDCl_3_) of 4c**

**
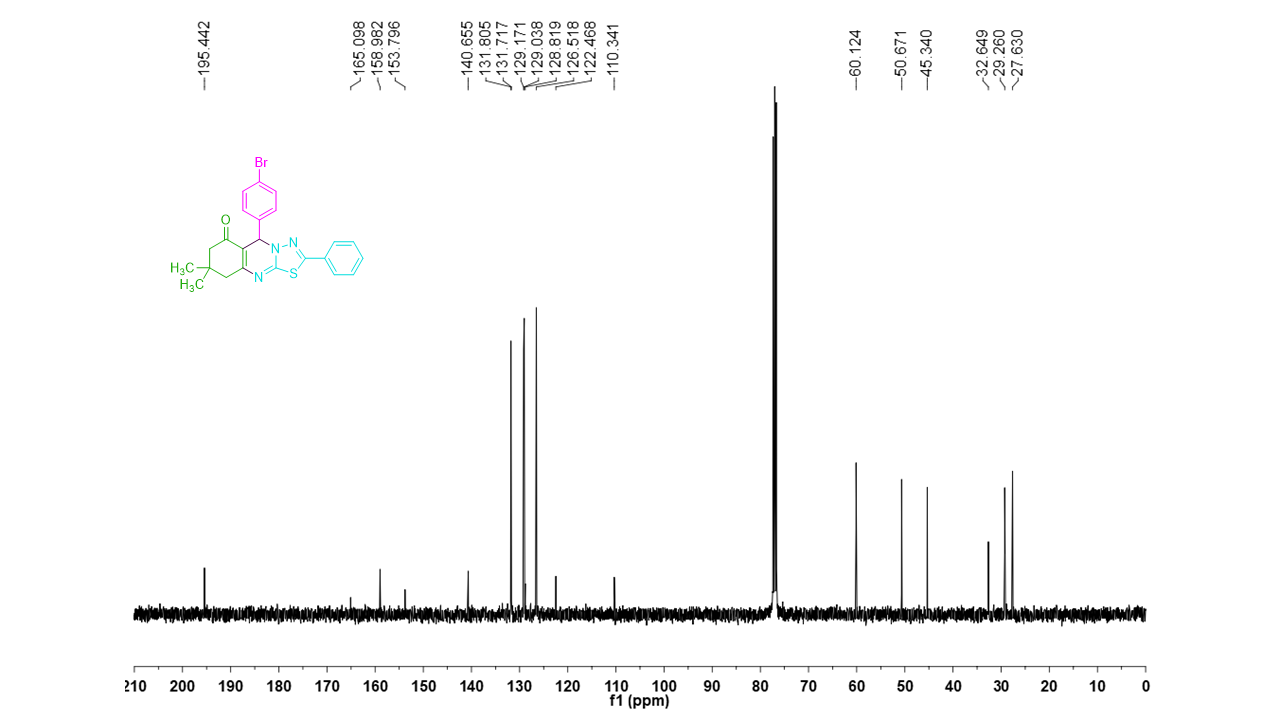
**

**Figure S7. ^13^C NMR (100 MHz, CDCl_3_) of 4c**

**
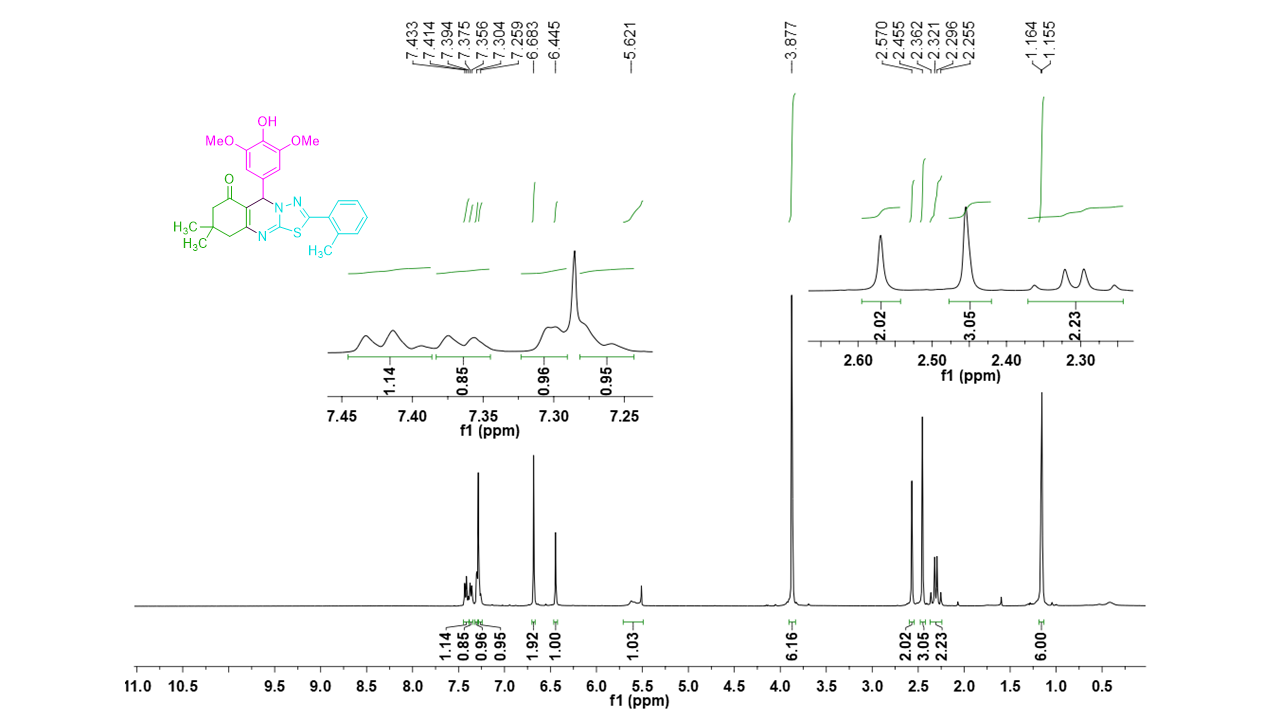
**

**Figure S8. ^1^H NMR (400 MHz, CDCl_3_) of 4d**

**
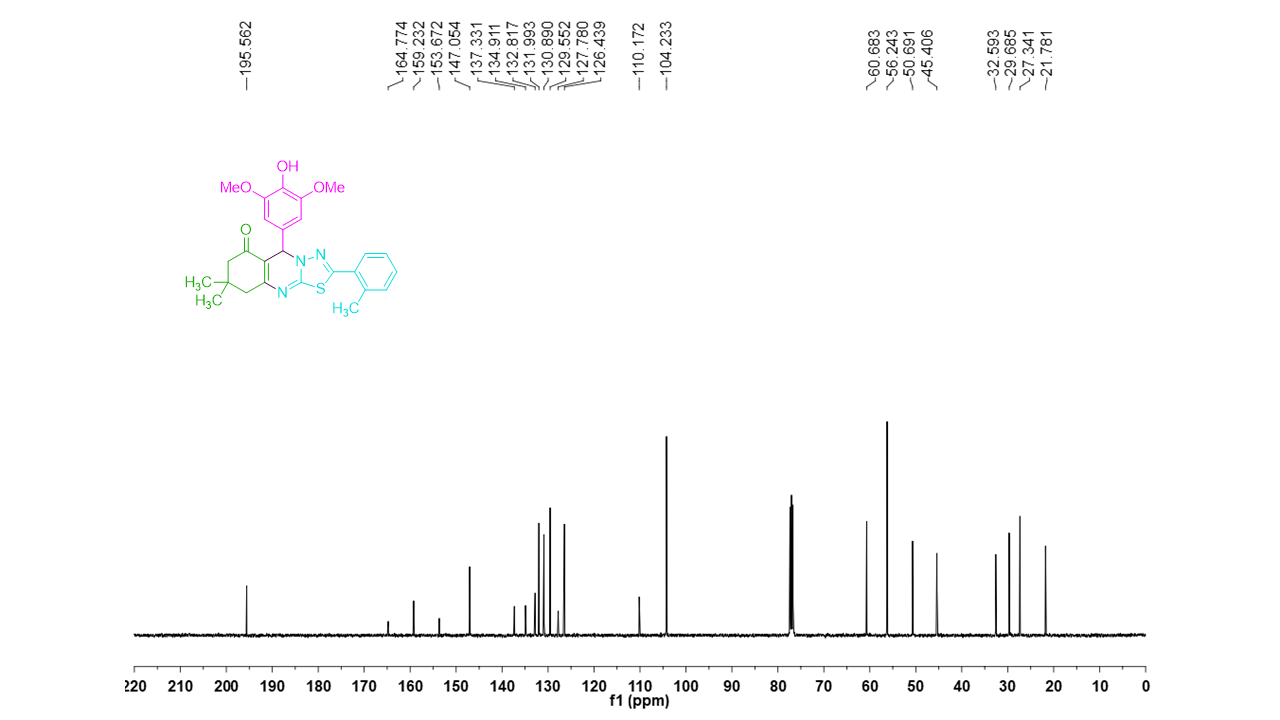
**

**Figure S9. ^13^C NMR (100 MHz, CDCl_3_) of 4d**

**
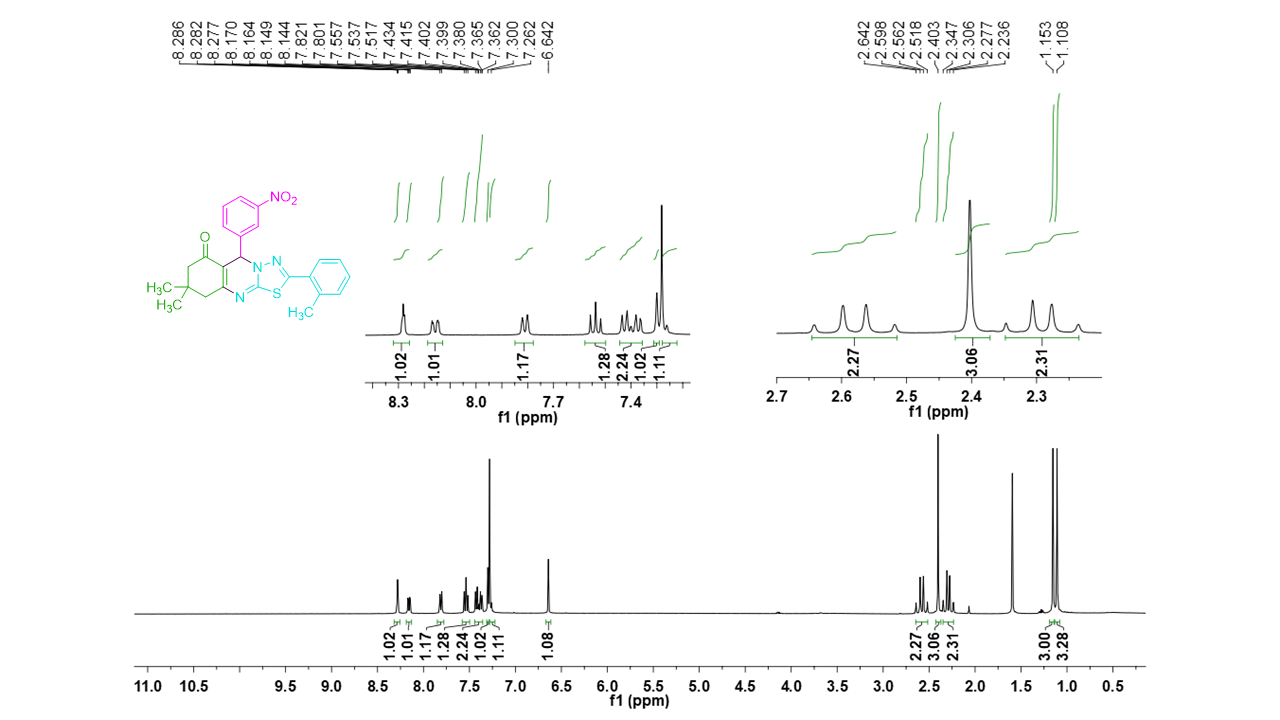
**

**Figure S10. ^1^H NMR (400 MHz, CDCl_3_) of 4e**

**
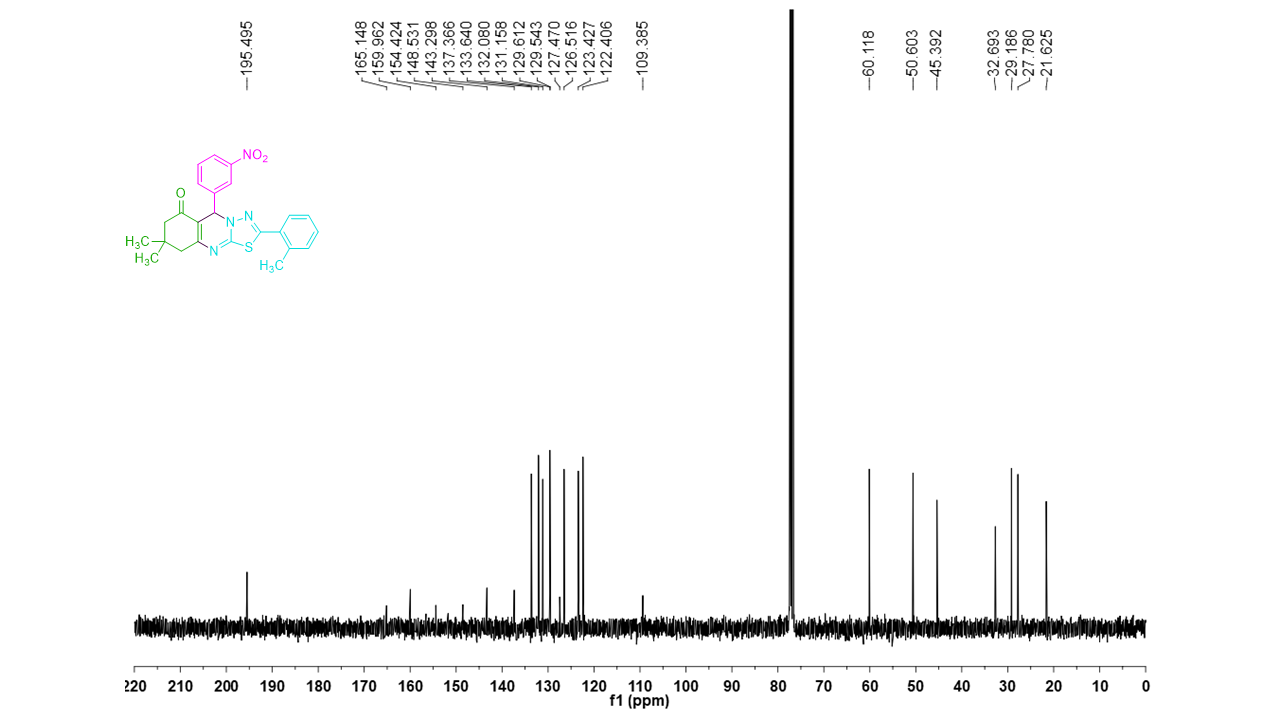
**

**Figure S11. ^13^C NMR (100 MHz, CDCl_3_) of 4e**

**
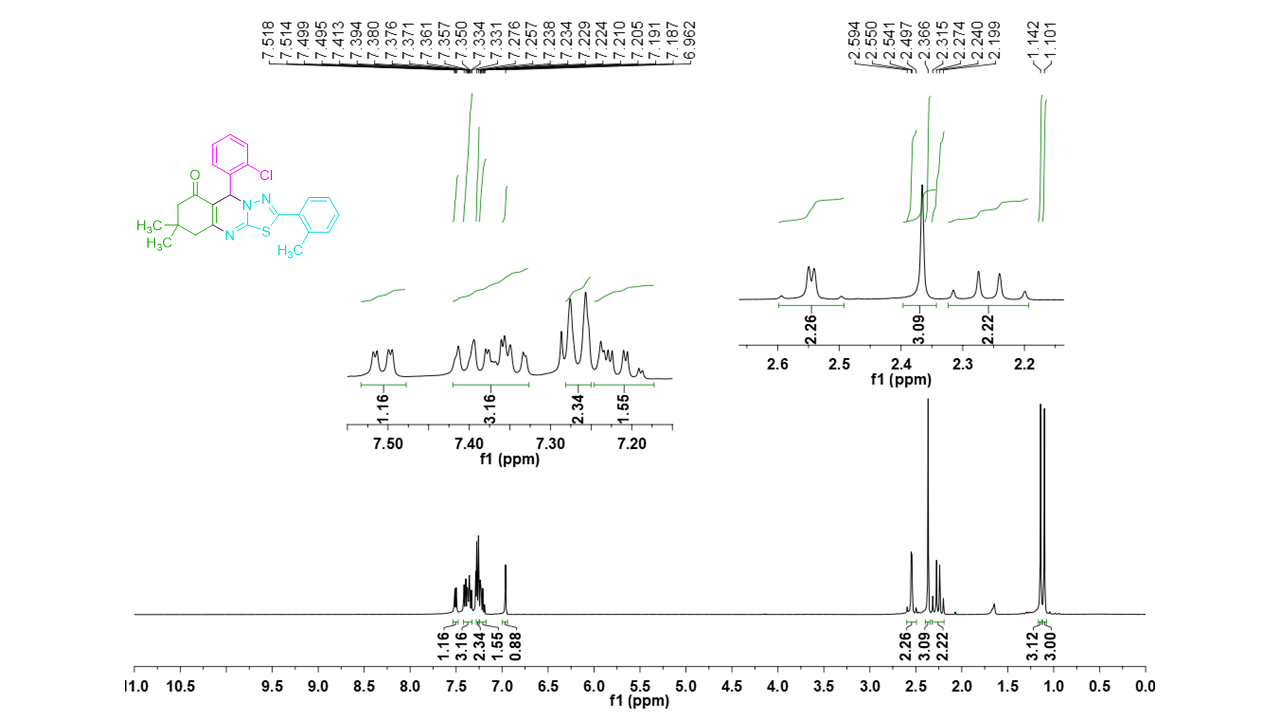
**

**Figure S12. ^1^H NMR (400 MHz, CDCl_3_) of 4f**

**
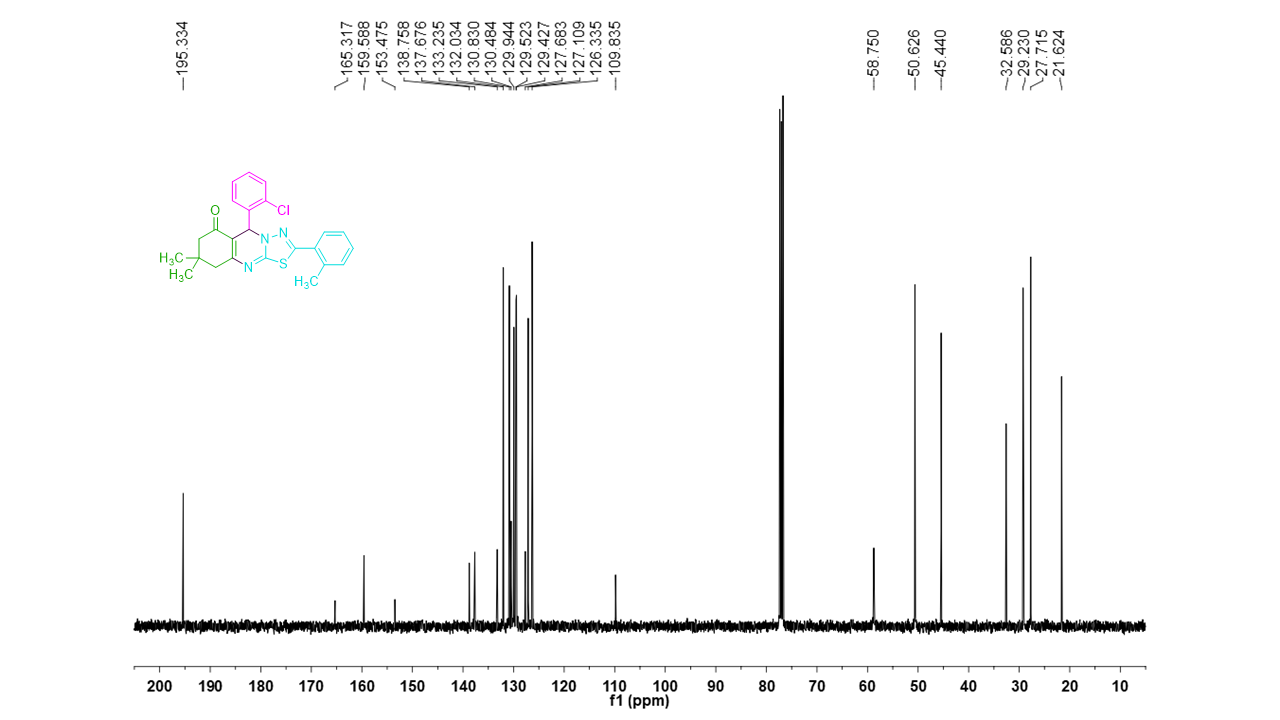
**

**Figure S13. ^13^C NMR (100 MHz, CDCl_3_) of 4f**

**
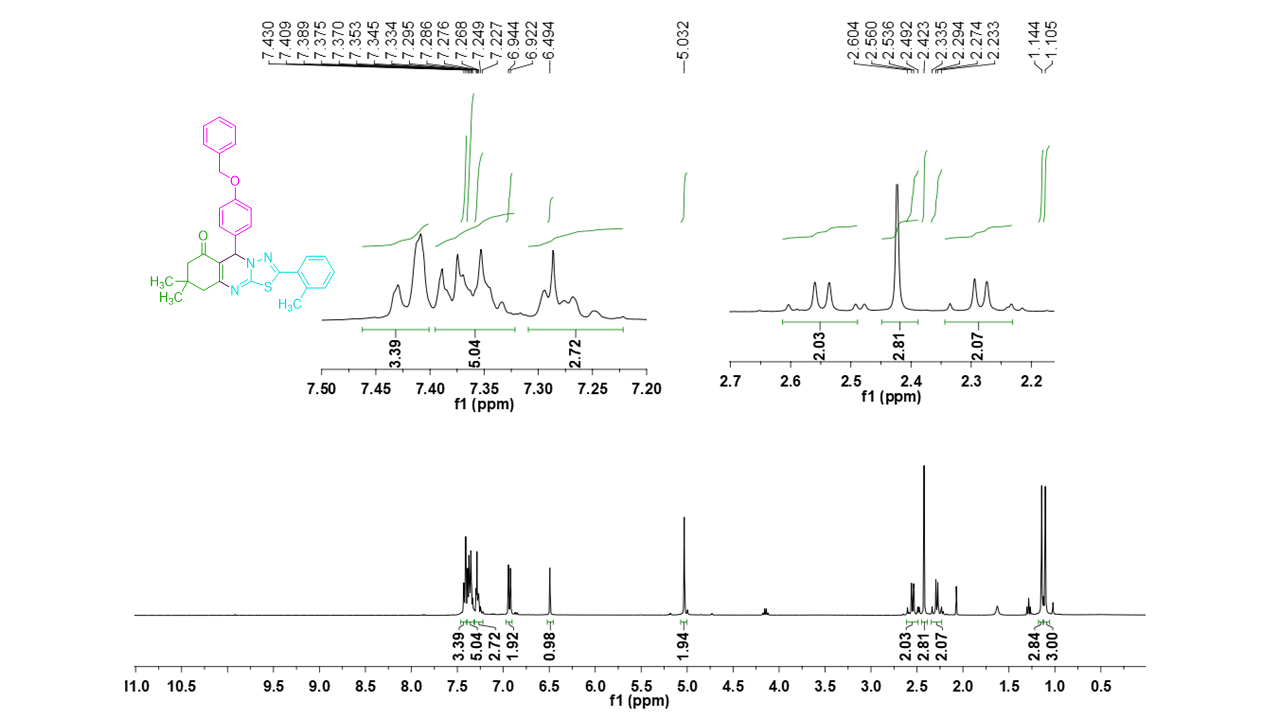
**

**Figure S14. ^1^H NMR (400 MHz, CDCl_3_) of 4g**

**
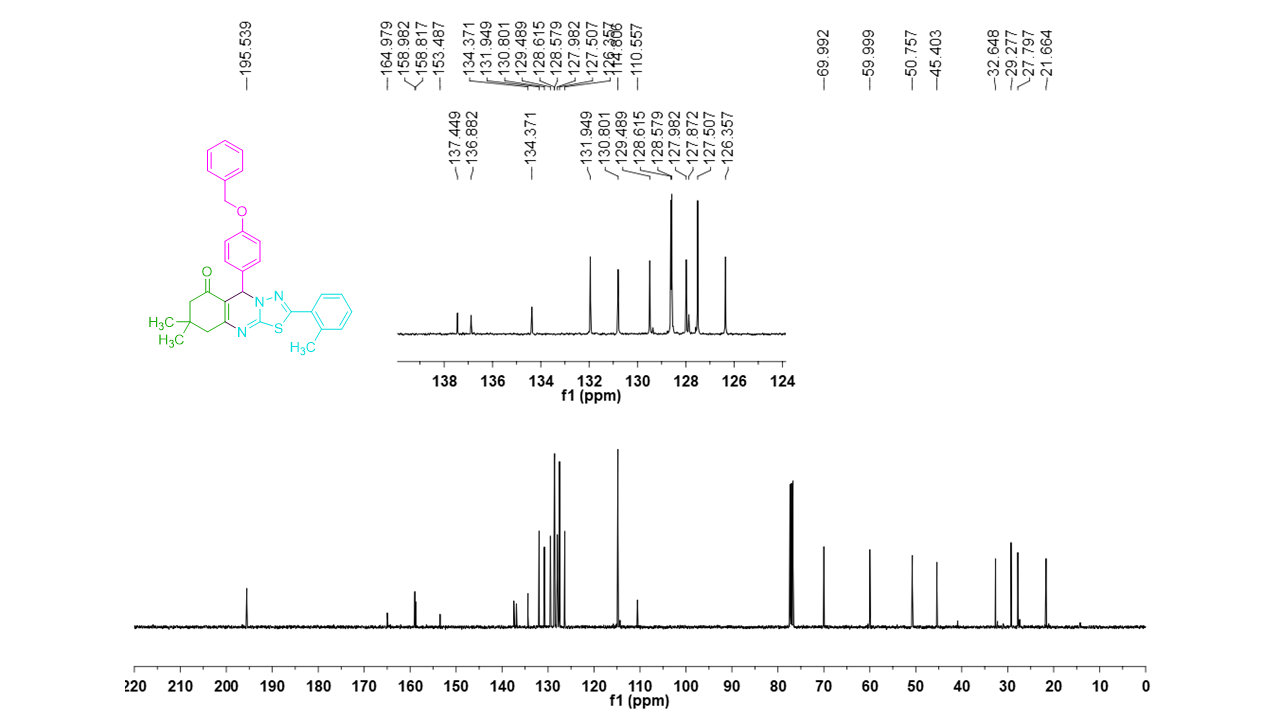
**

**Figure S15. ^13^C NMR (100 MHz, CDCl_3_) of 4g**

**
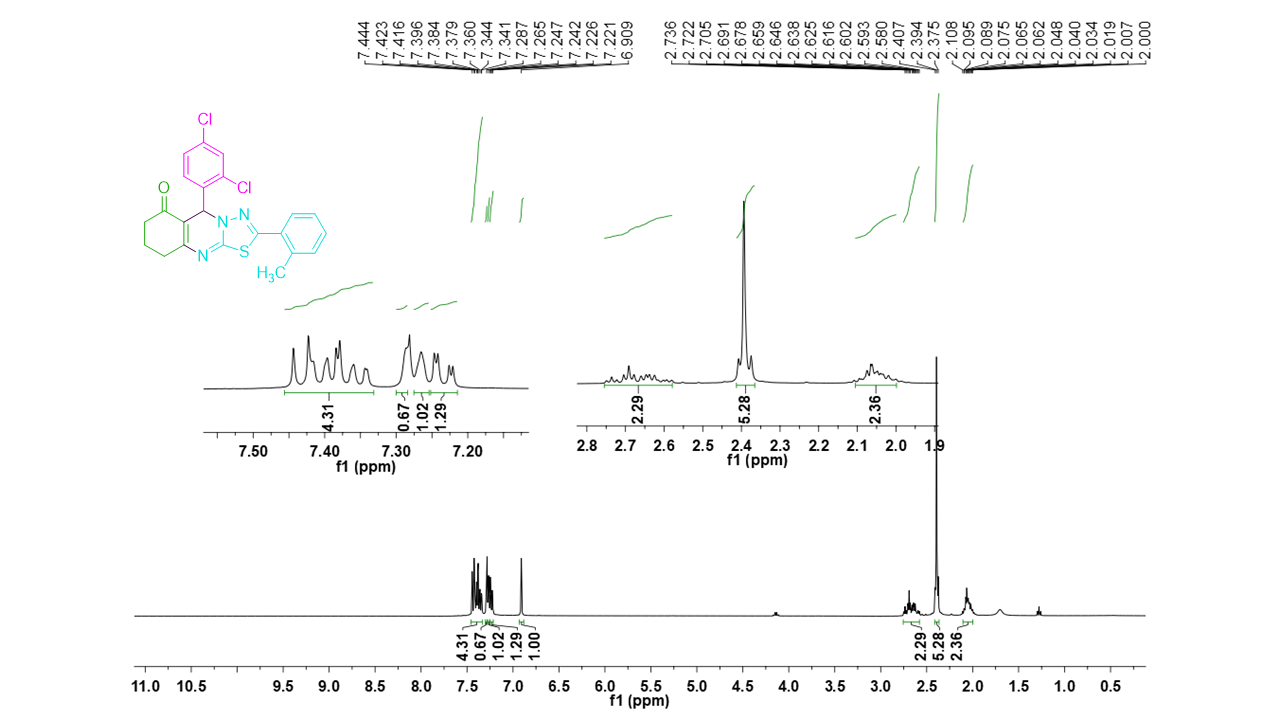
**

**Figure S16. ^1^H NMR (400 MHz, CDCl_3_) of 4h**

**
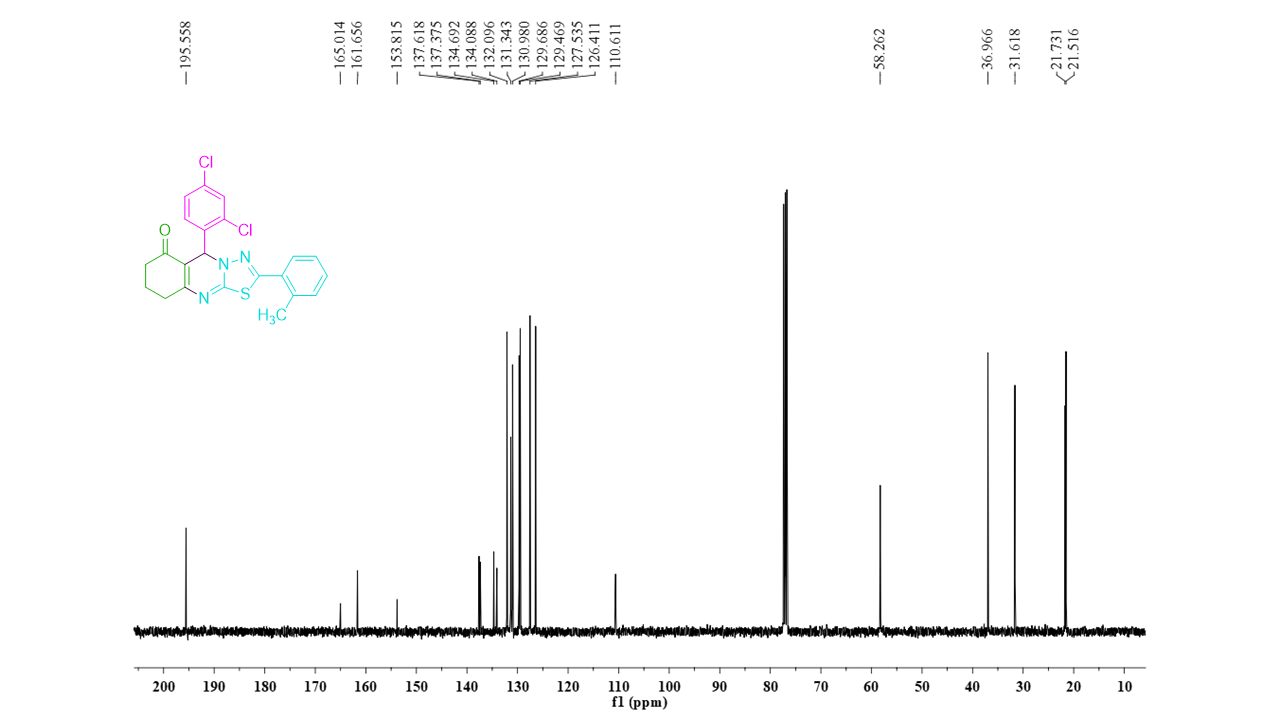
**

**Figure S17. ^13^C NMR (100 MHz, CDCl_3_) of 4h**

**
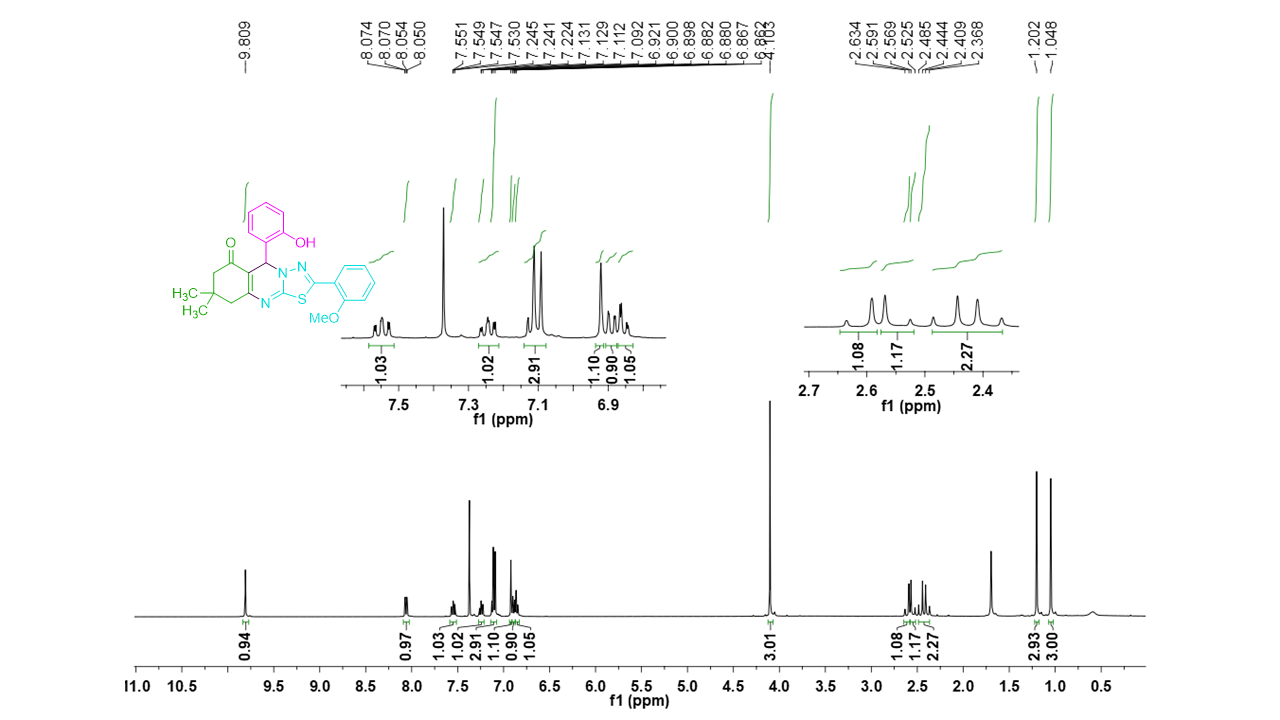
**

**Figure S18. ^1^H NMR (400 MHz, CDCl_3_) of 4i**

**
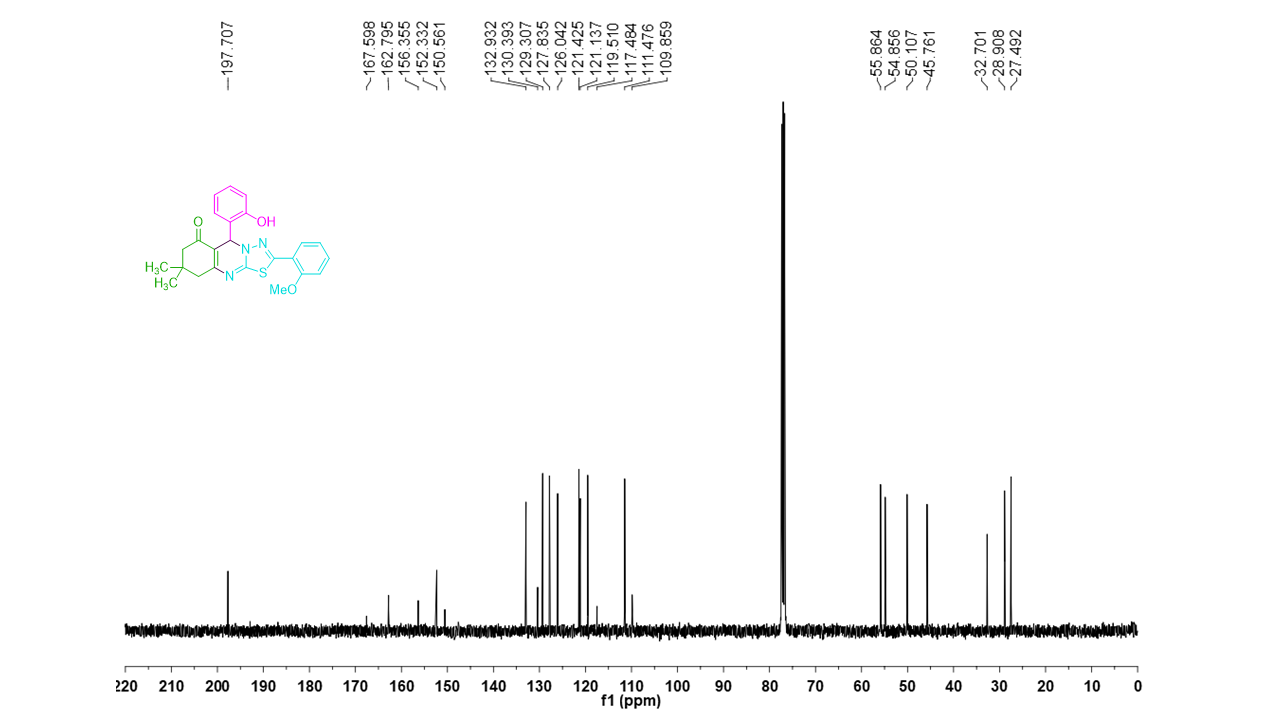
**

**Figure S19. ^13^C NMR (100 MHz, CDCl_3_) of 4i**

**
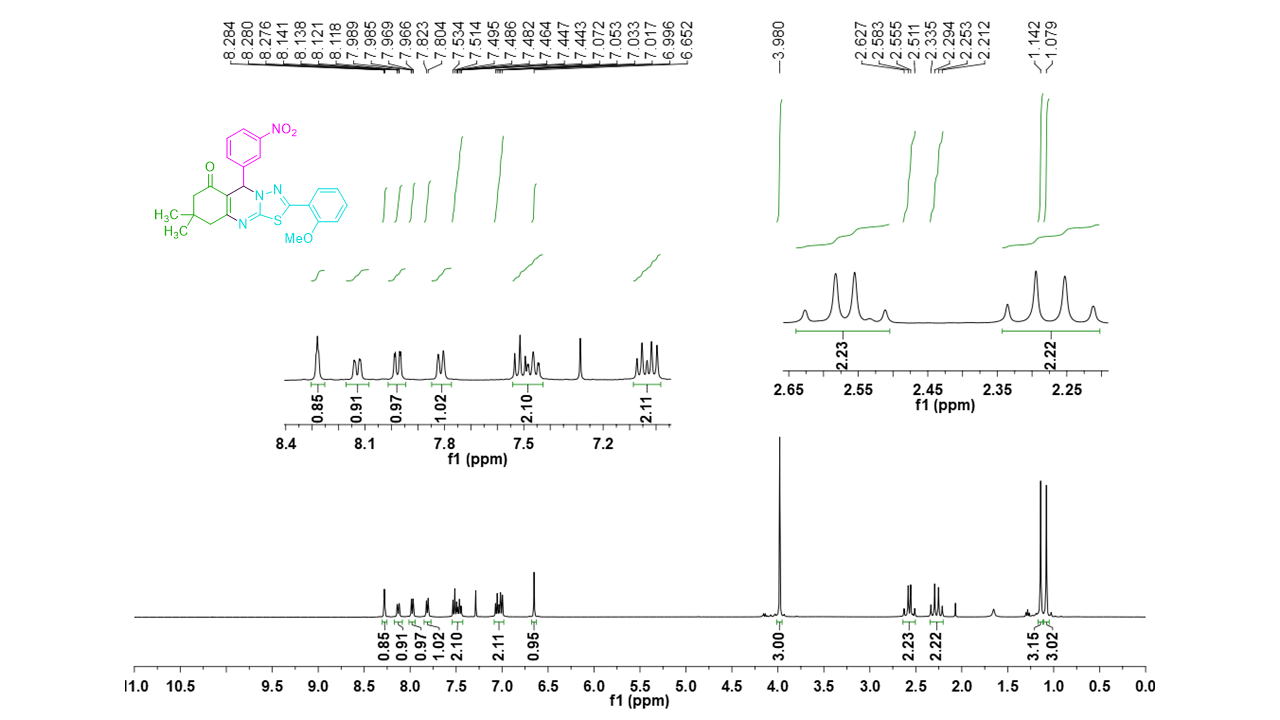
**

**Figure S20. ^1^H NMR (400 MHz, CDCl_3_) of 4j**

**
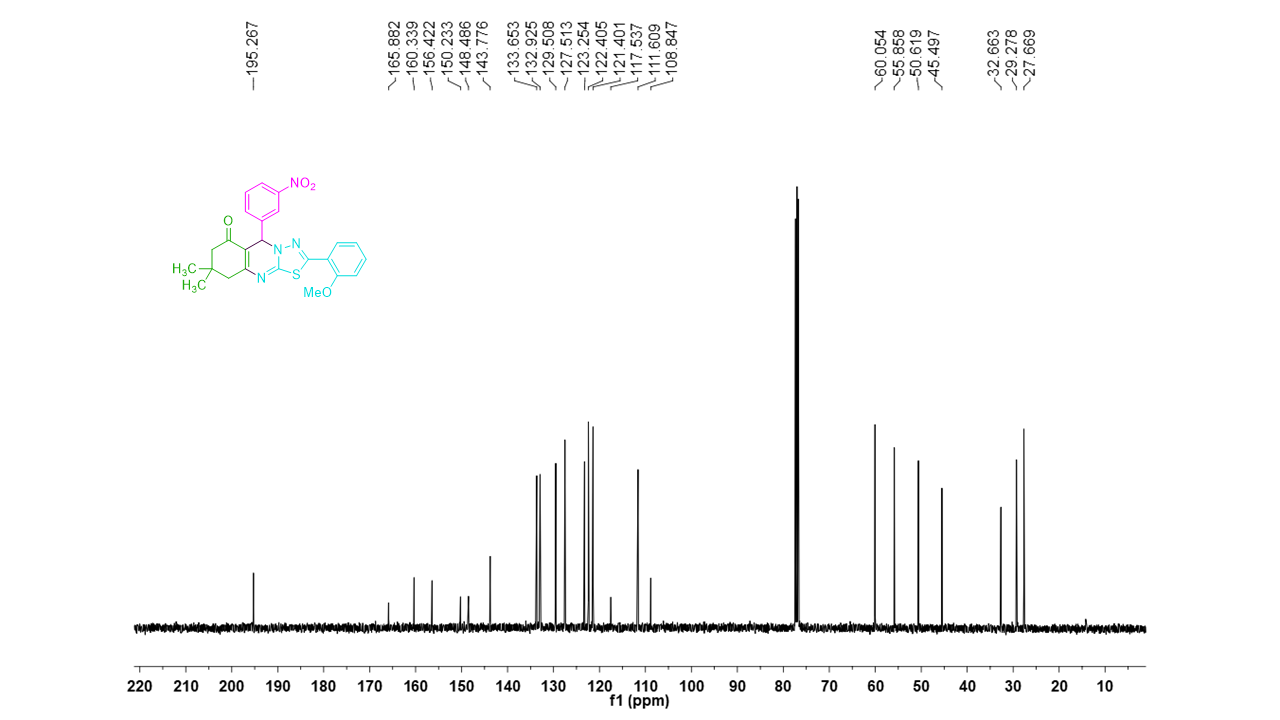
**

**Figure S21. ^13^C NMR (100 MHz, CDCl_3_) of 4j**

**
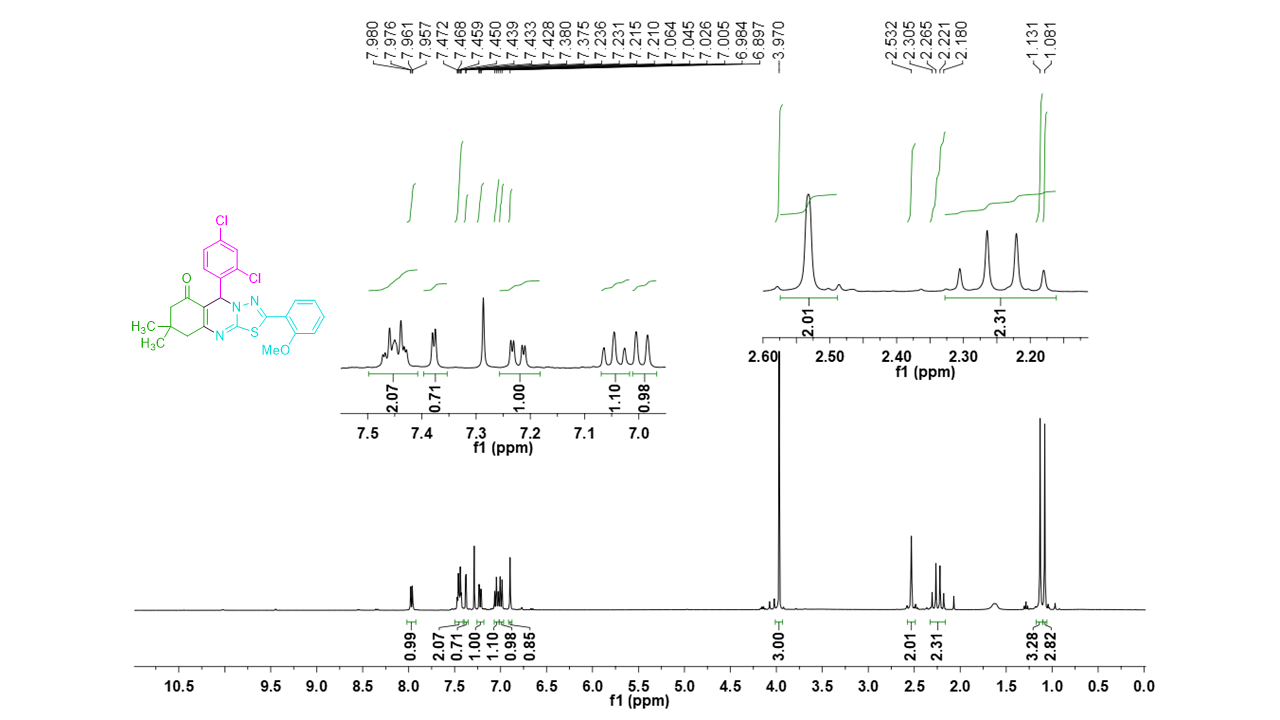
**

**Figure S22. ^1^H NMR (400 MHz, CDCl_3_) of 4k**

**
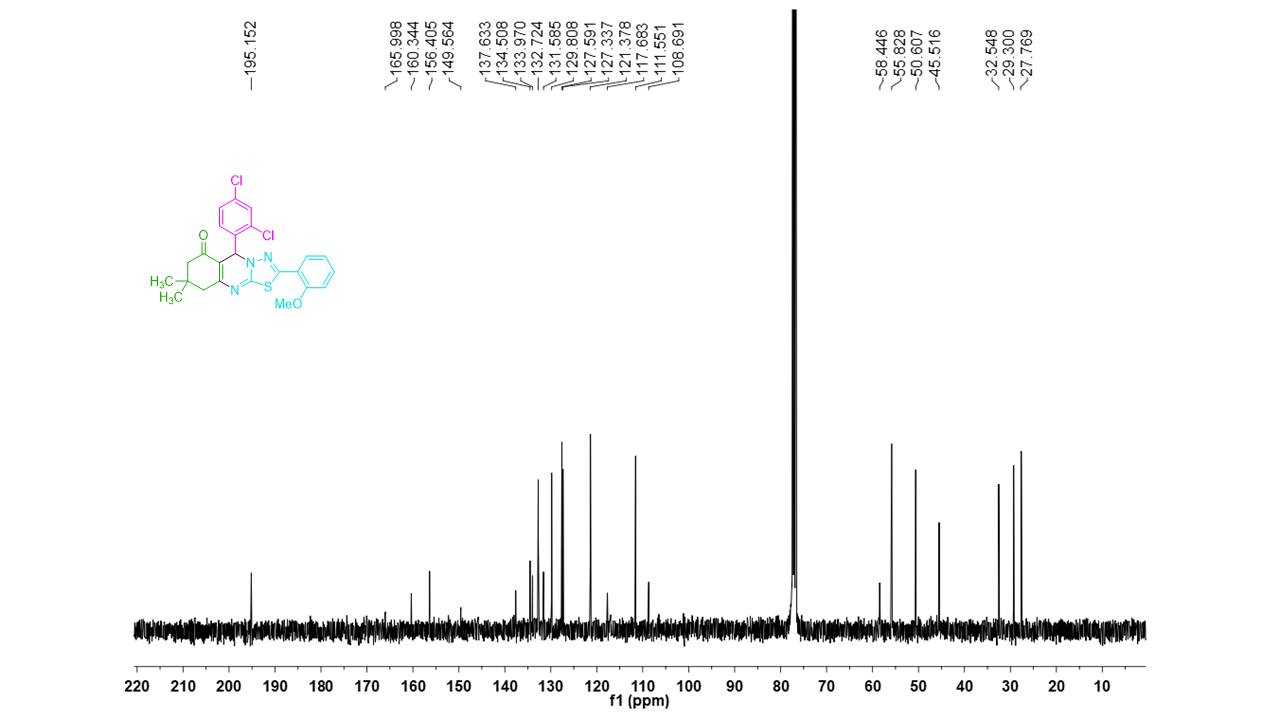
**

**Figure S23. ^13^C NMR (100 MHz, CDCl_3_) of 4k**

**
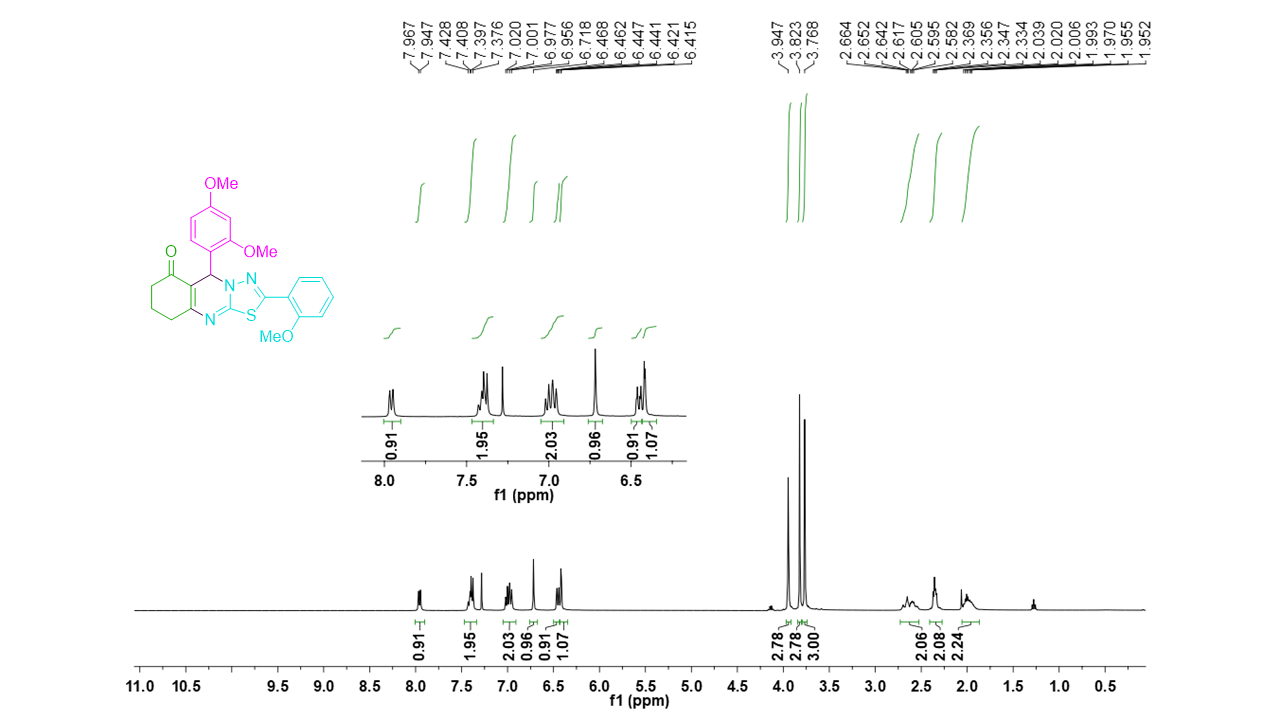
**

**Figure S24. ^1^H NMR (400 MHz, CDCl_3_) of 4l**

**
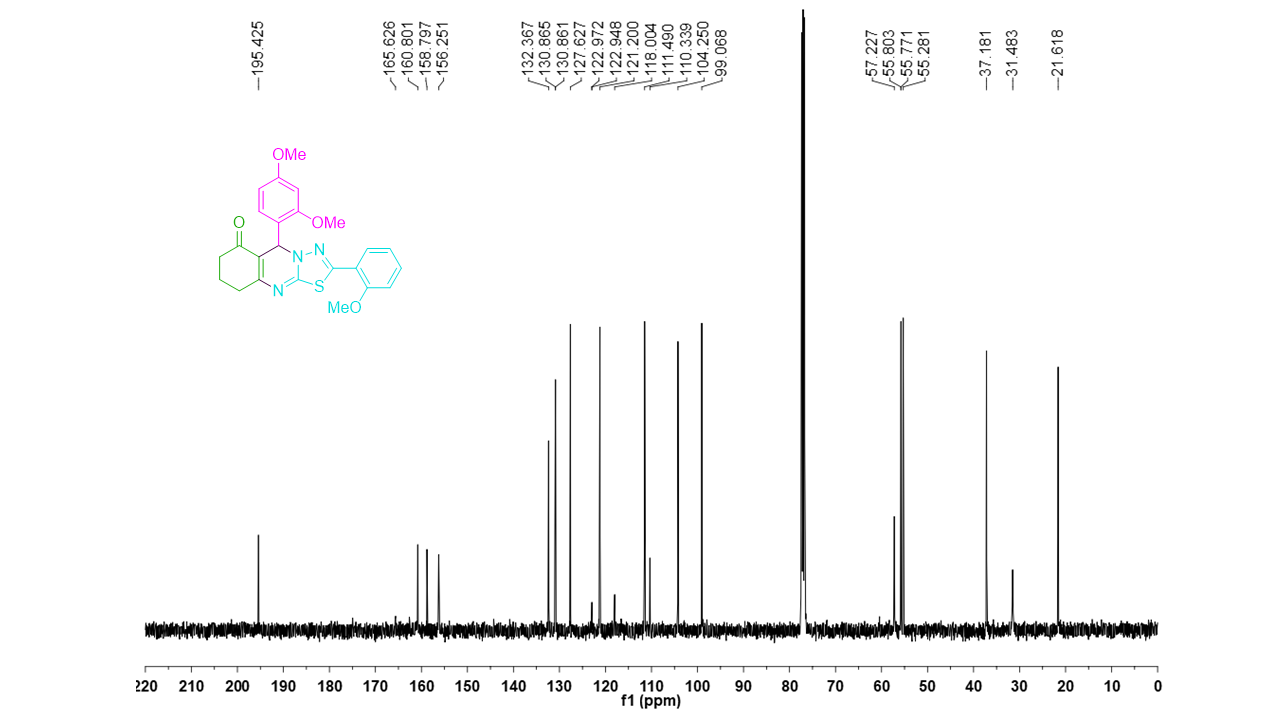
**

**Figure S25. ^13^C NMR (100 MHz, CDCl_3_) of 4l**

**
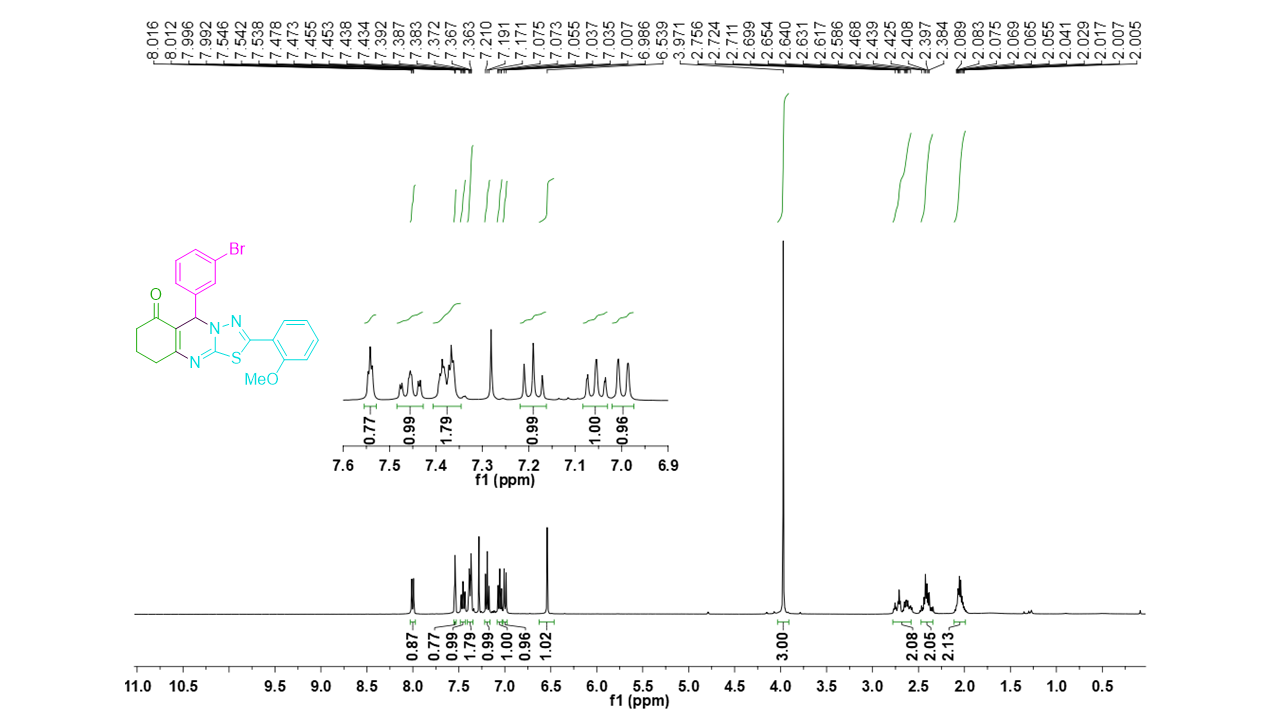
**

**Figure S26. ^1^H NMR (400 MHz, CDCl_3_) of 4m**

**
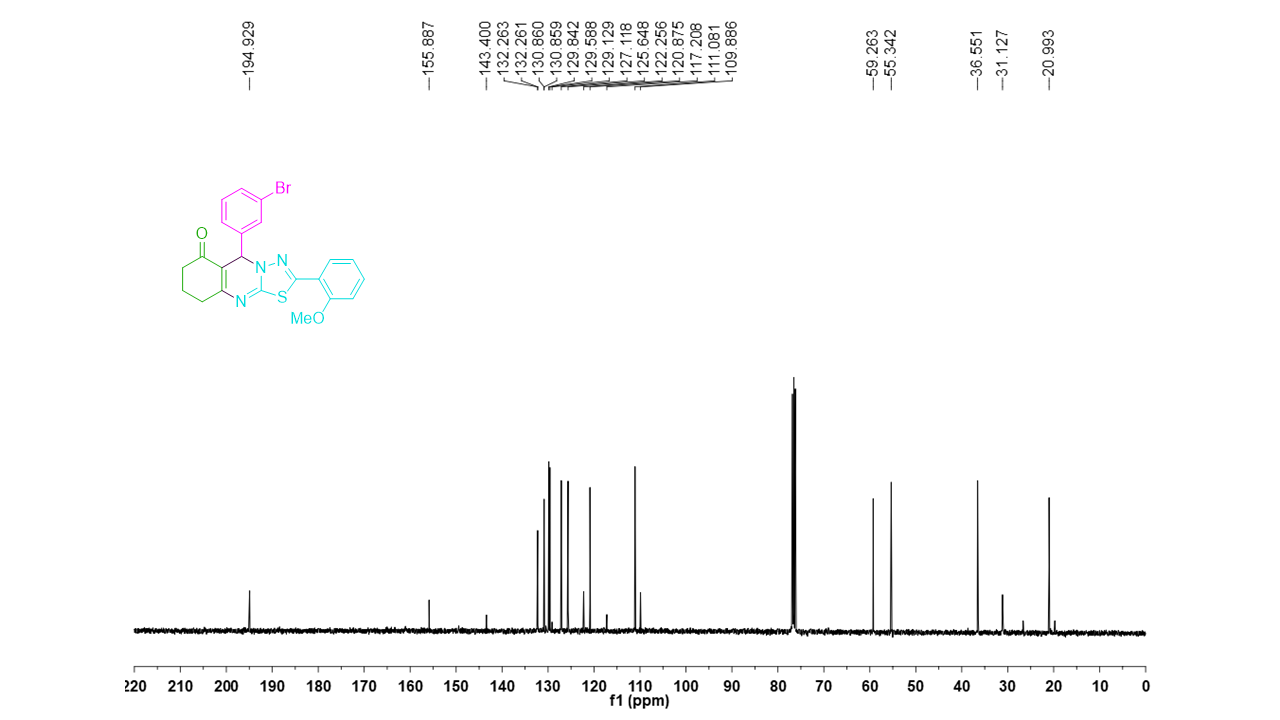
**

**Figure S27. ^13^C NMR (100 MHz, CDCl_3_) of 4m**

**
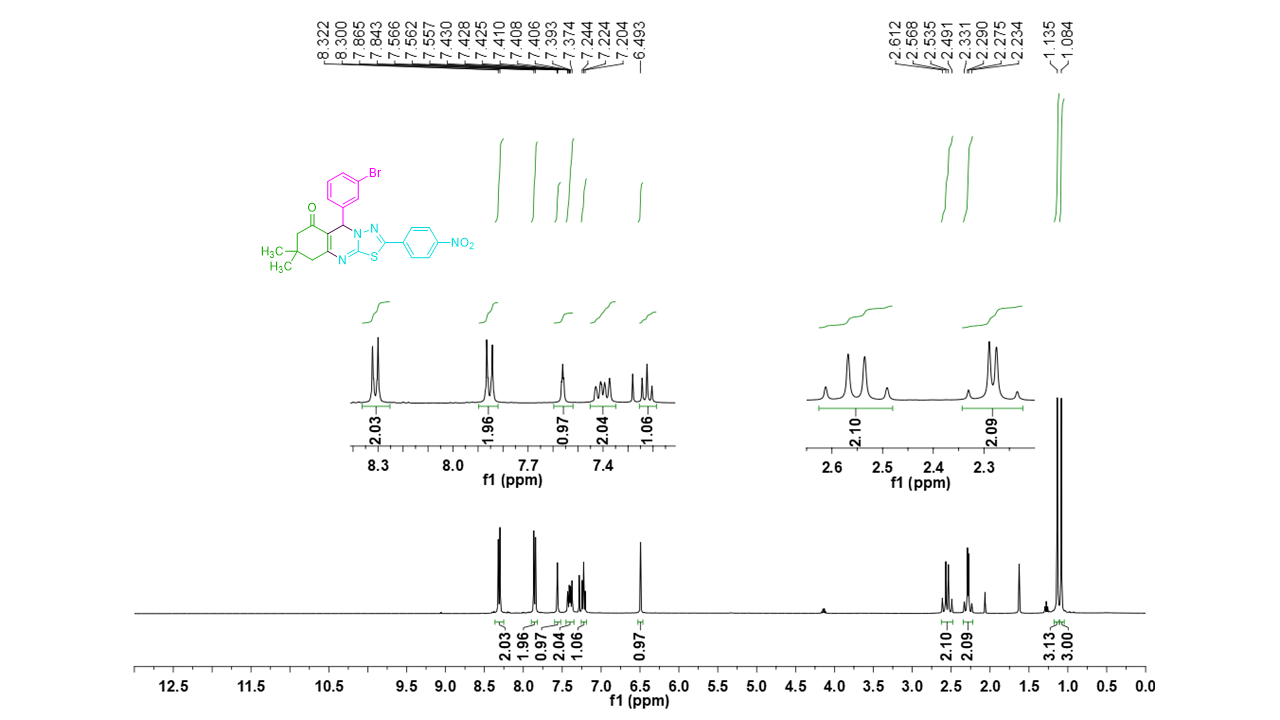
**

**Figure S28. ^1^H NMR (400 MHz, CDCl_3_) of 4n**

**
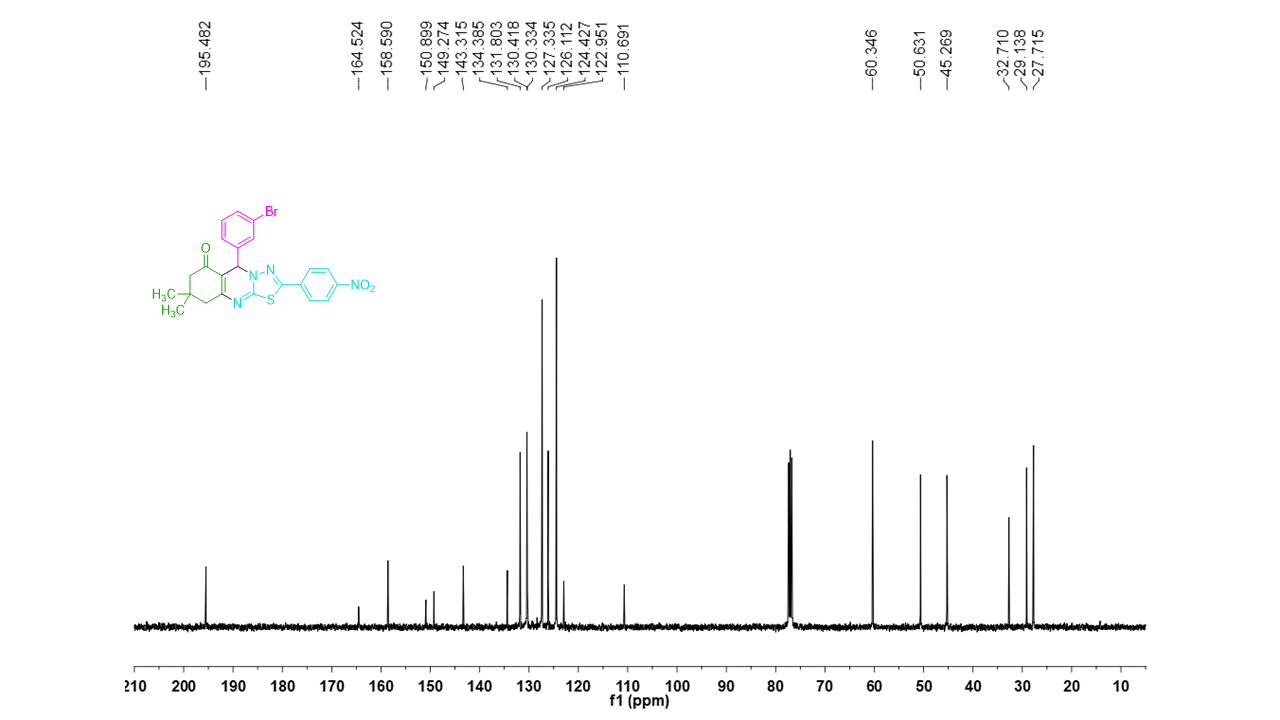
**

**Figure S29. ^13^C NMR (100 MHz, CDCl_3_) of 4n**

**
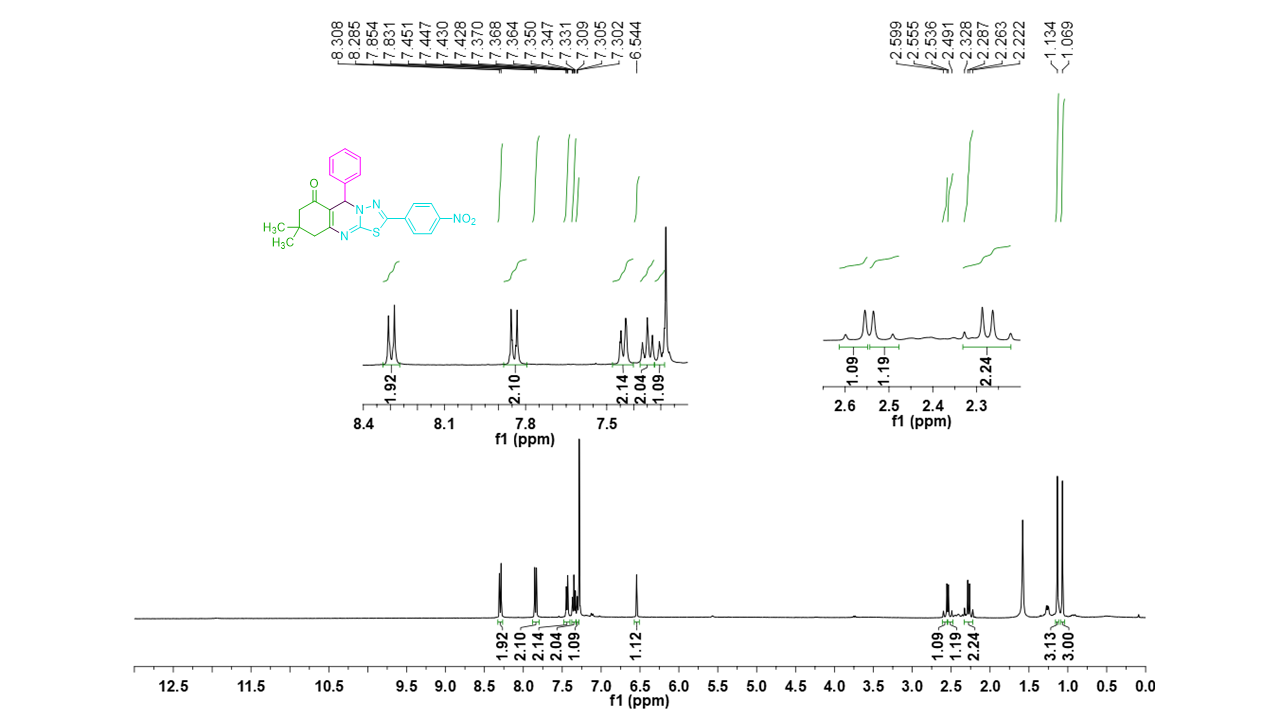
**

**Figure S30. ^1^H NMR (400 MHz, CDCl_3_) of 4o**

**
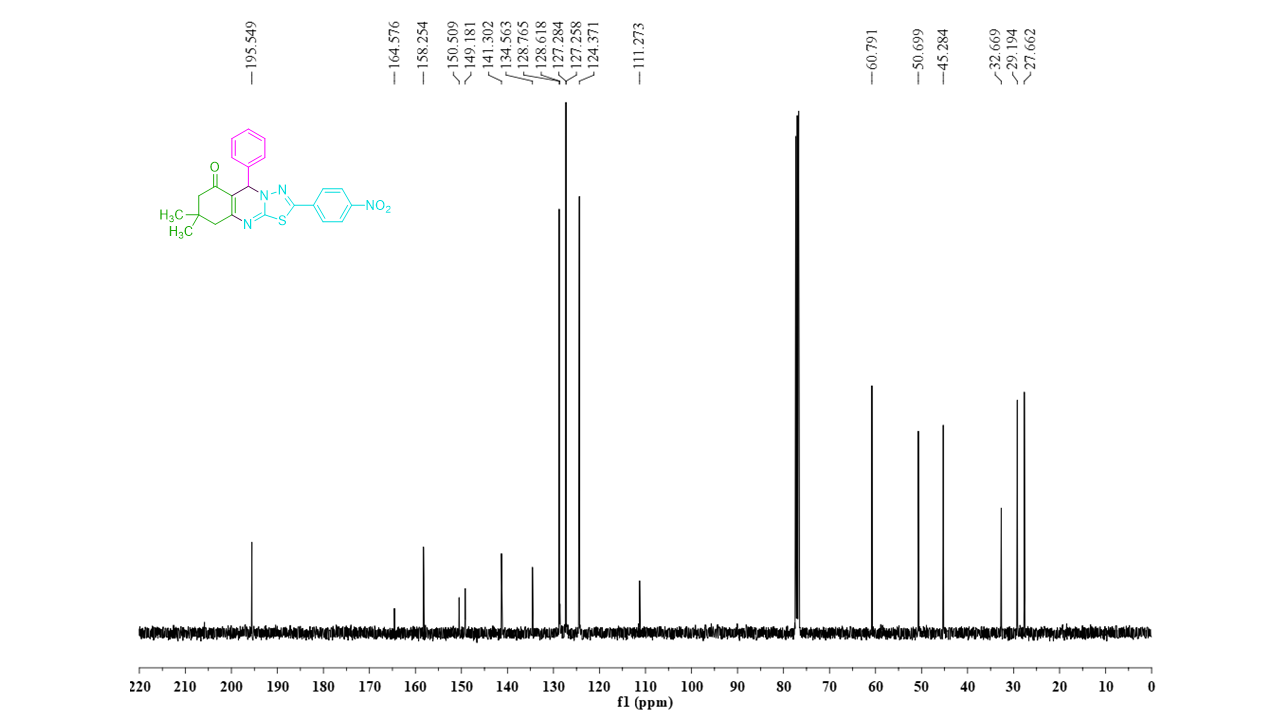
**

**Figure S31. ^13^C NMR (100 MHz, CDCl_3_) of 4o**

**
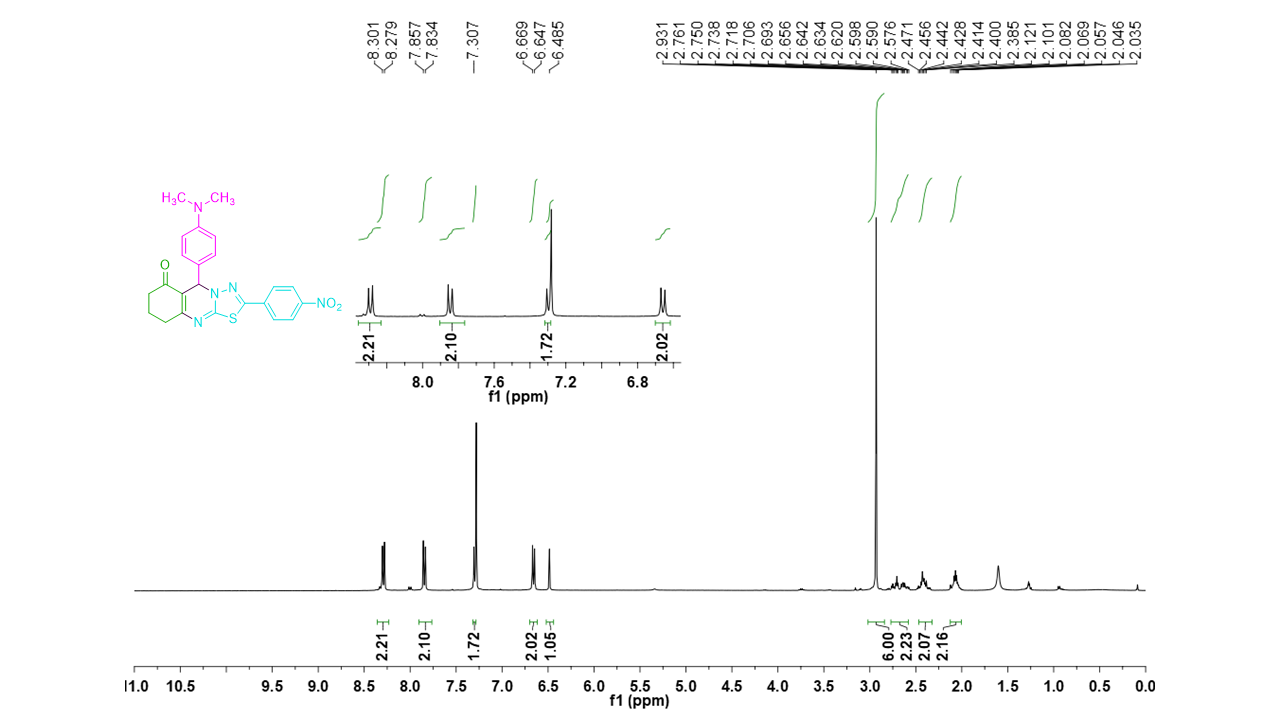
**

**Figure S32. ^1^H NMR (400 MHz, CDCl_3_) of 4p**

**
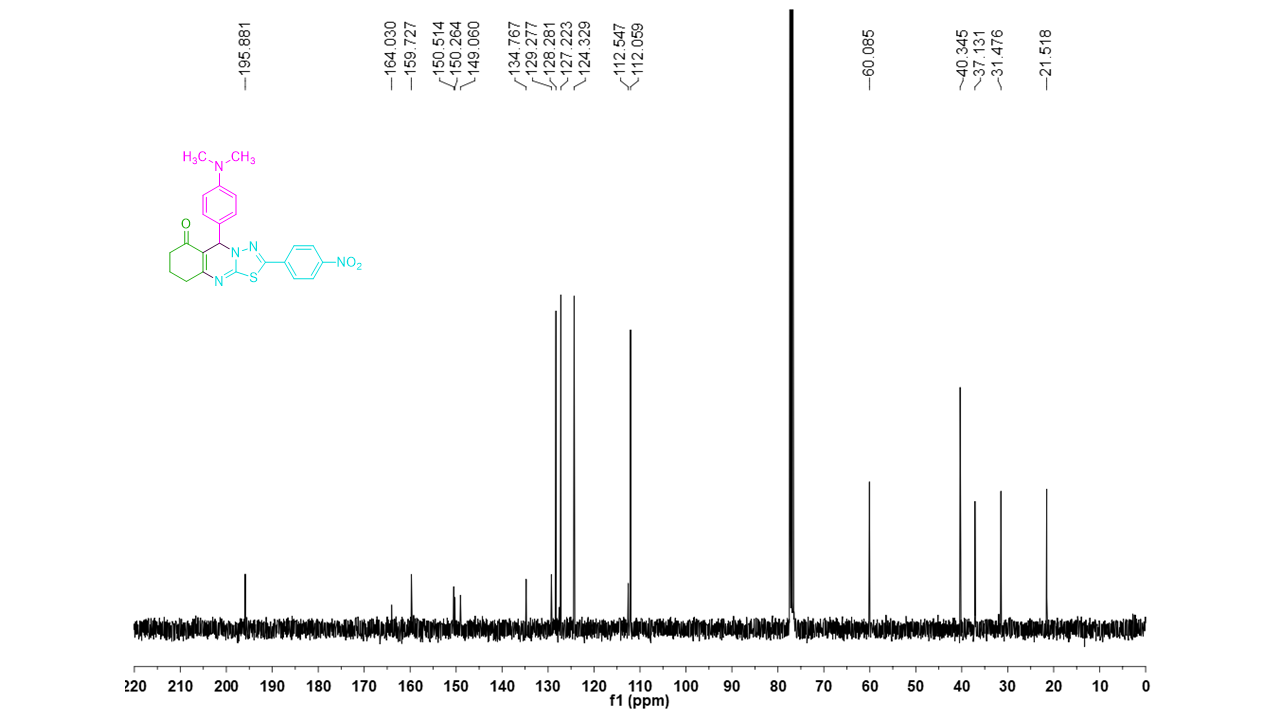
**

**Figure S33. ^13^C NMR (100 MHz, CDCl_3_) of 4p**

**
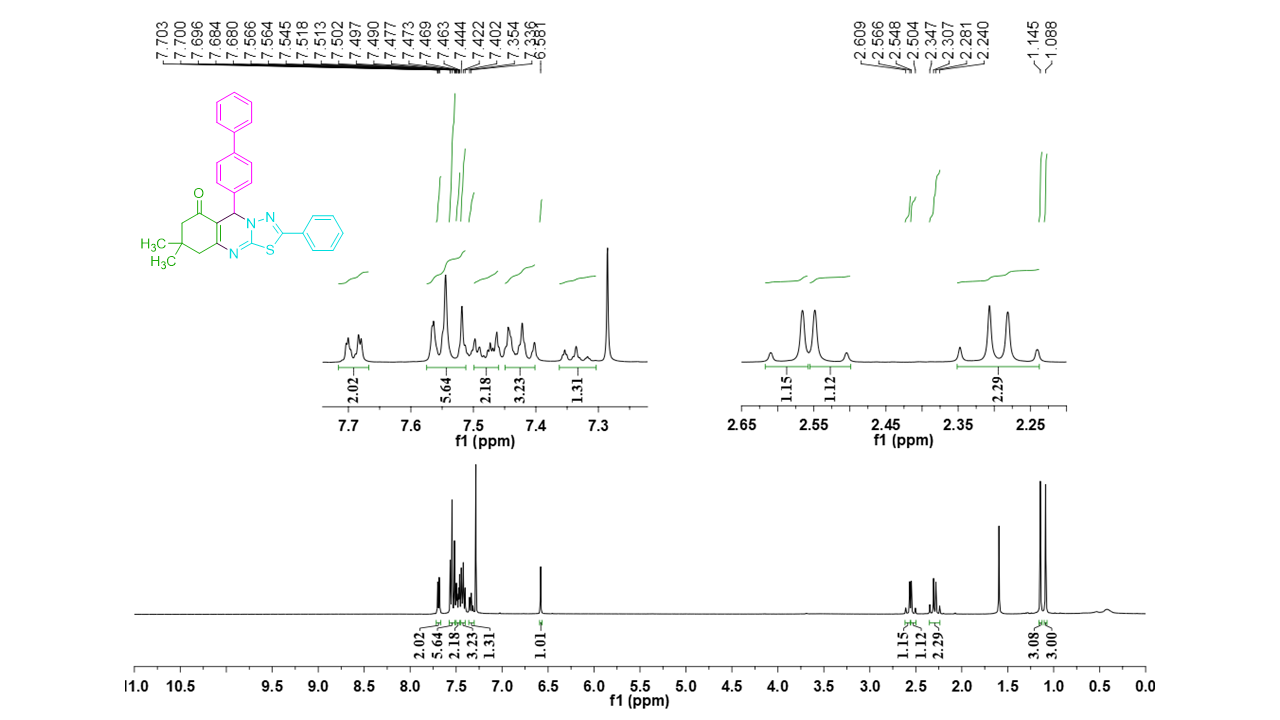
**

**Figure S34. ^1^H NMR (400 MHz, CDCl_3_) of 4q**

**
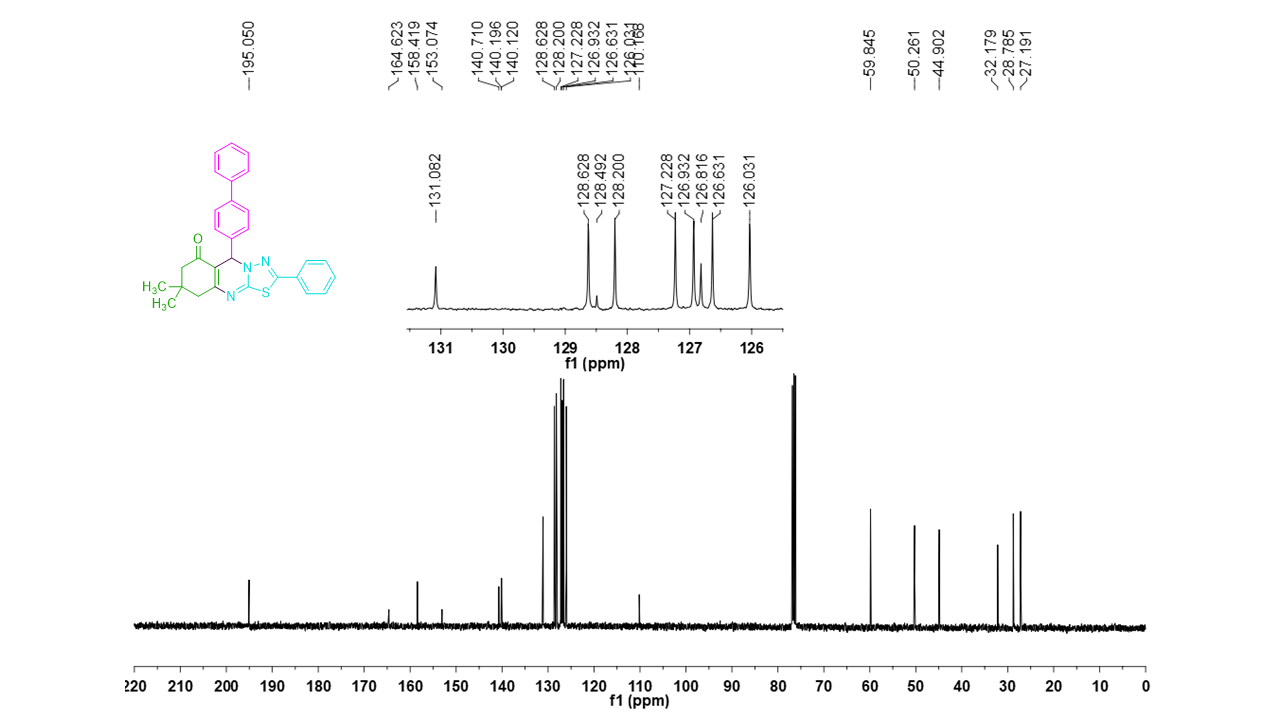
**

**Figure S35. ^13^C NMR (100 MHz, CDCl_3_) of 4q**

**
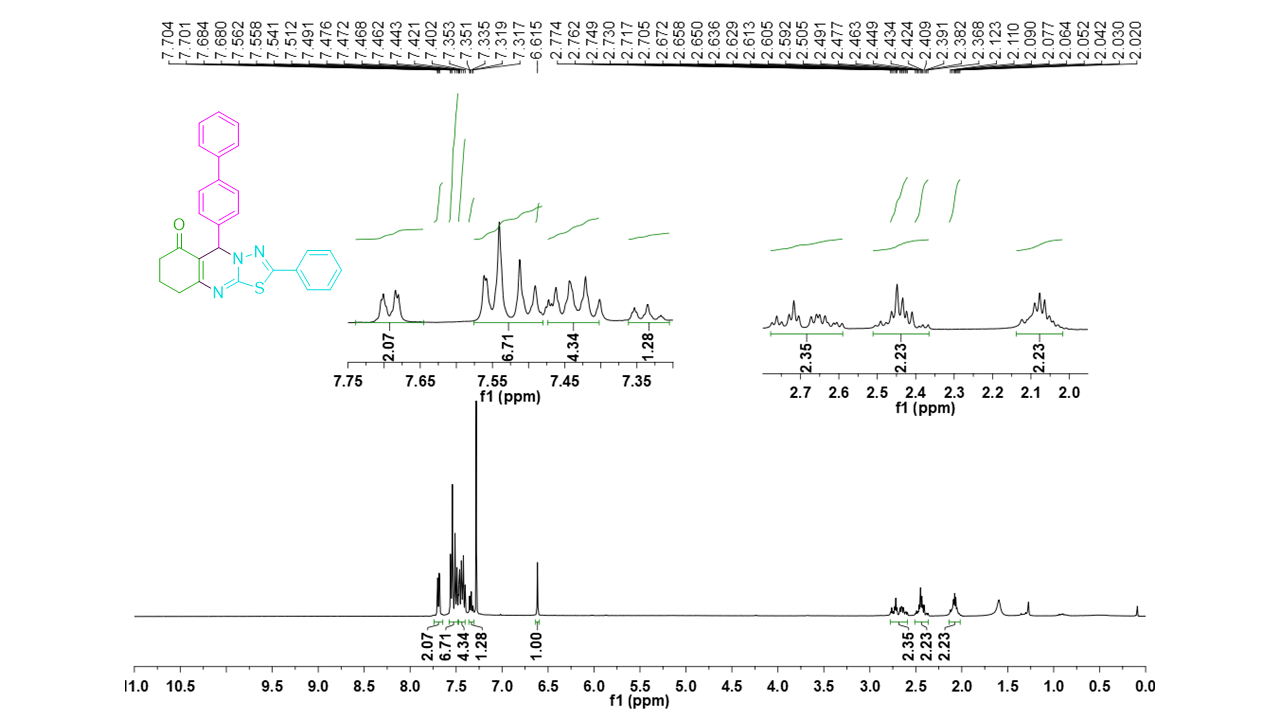
**

**Figure S36. ^1^H NMR (400 MHz, CDCl_3_) of 4r**

**
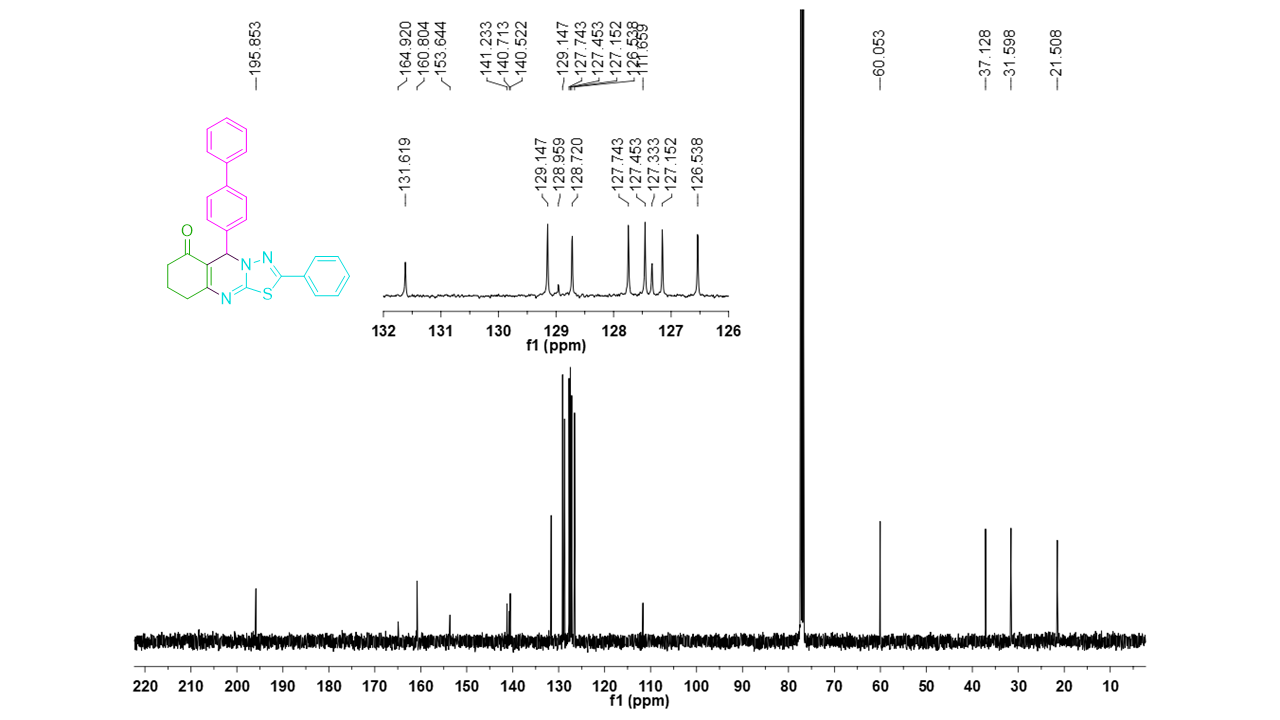
**

**Figure S37. ^13^C NMR (100 MHz, CDCl_3_) of 4r**

**
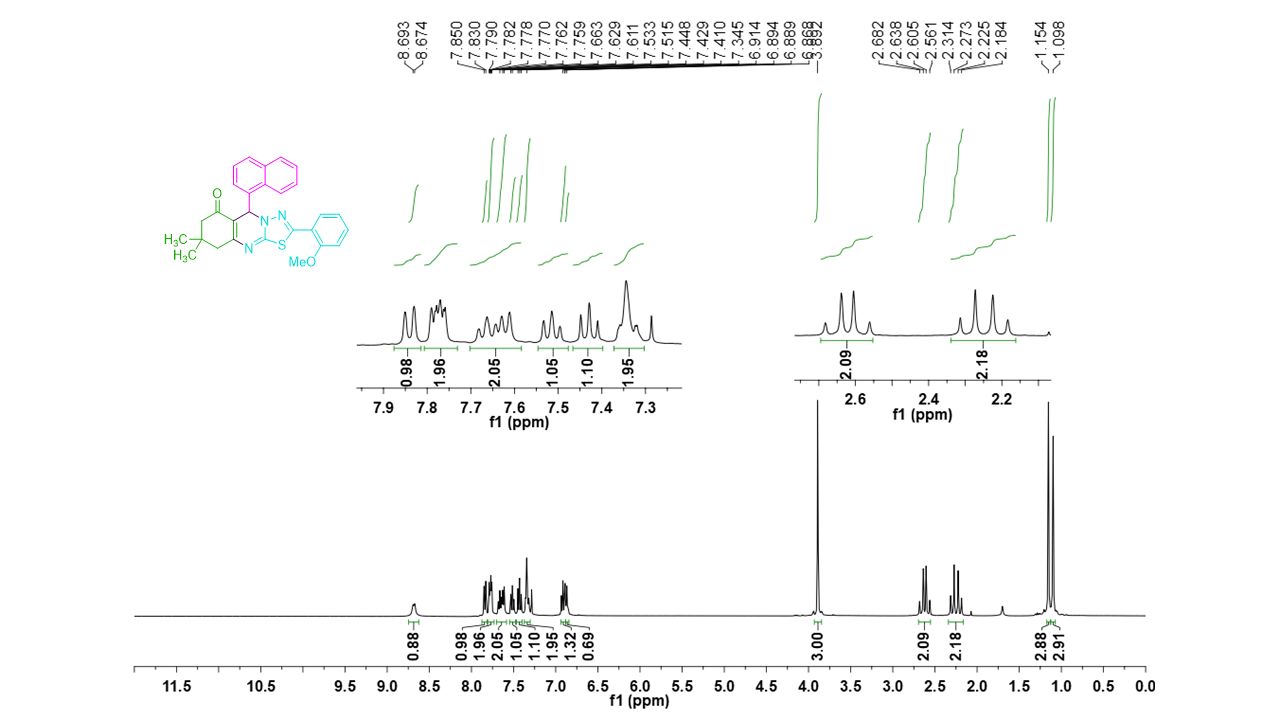
**

**Figure S38. ^1^H NMR (400 MHz, CDCl_3_) of 4s**

**
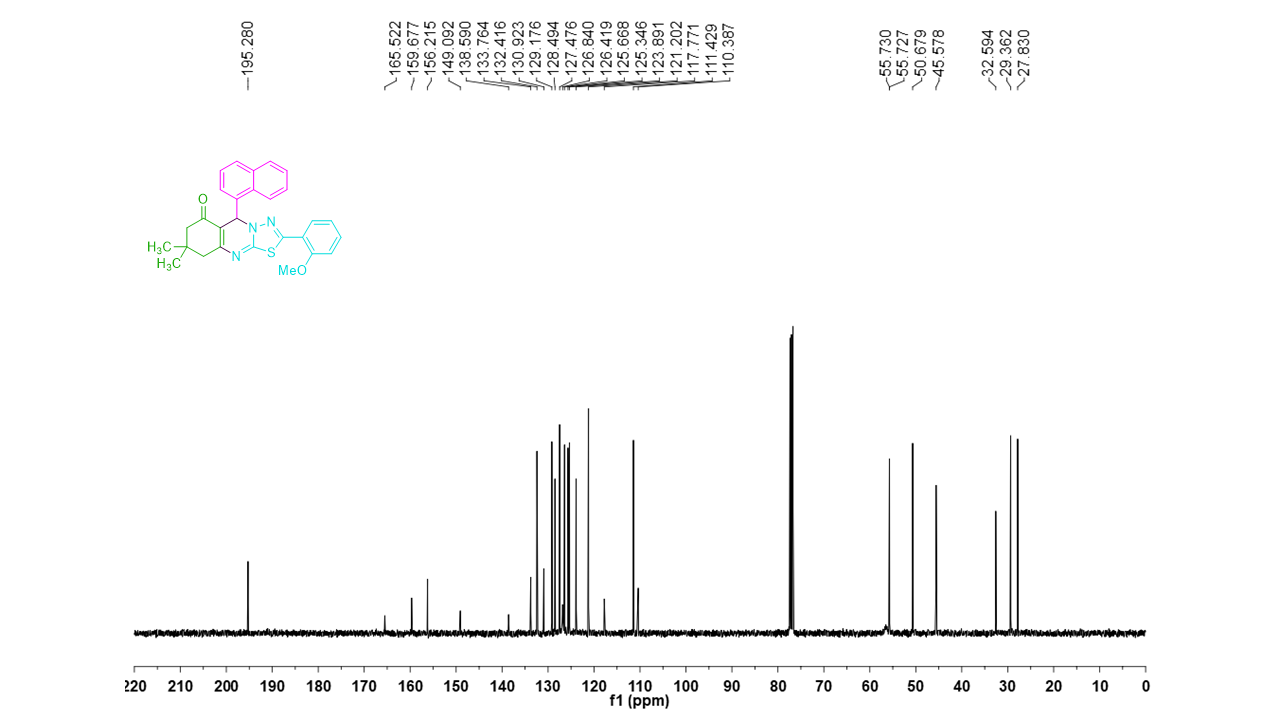
**

**Figure S39. ^13^C NMR (100 MHz, CDCl_3_) of 4s**

**
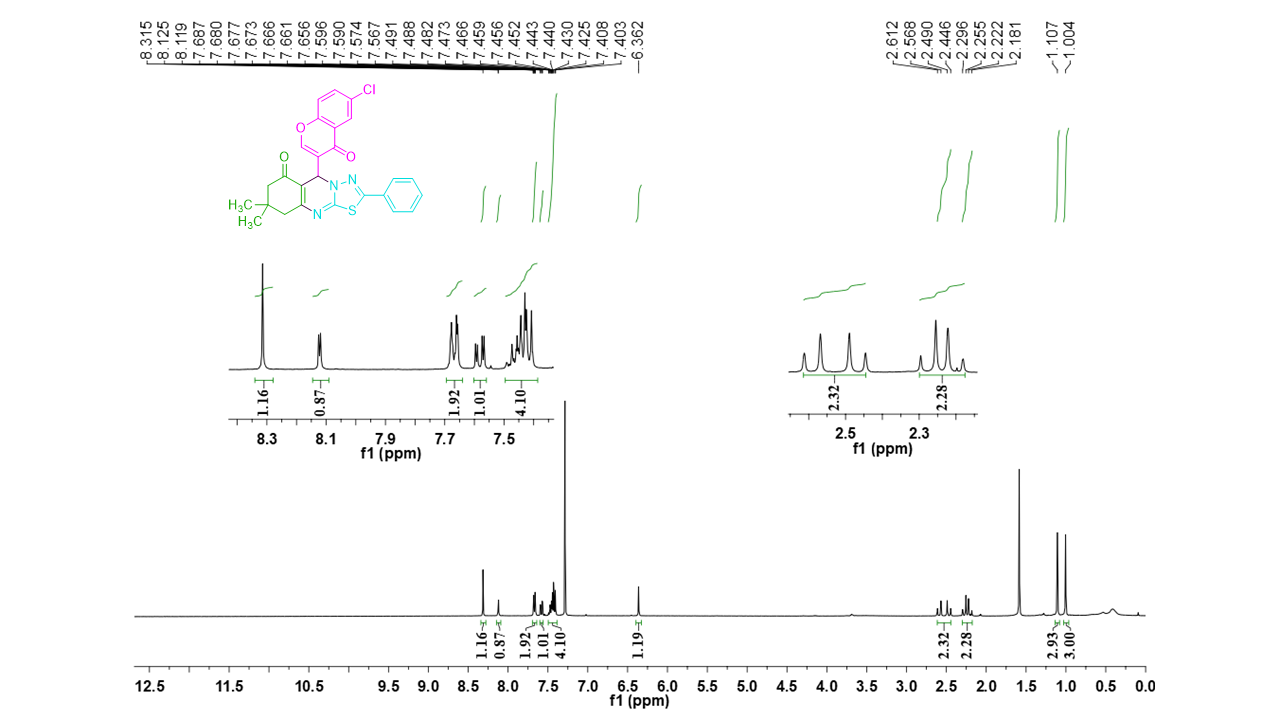
**

**Figure S40. ^1^H NMR (400 MHz, CDCl_3_) of 4t**

**
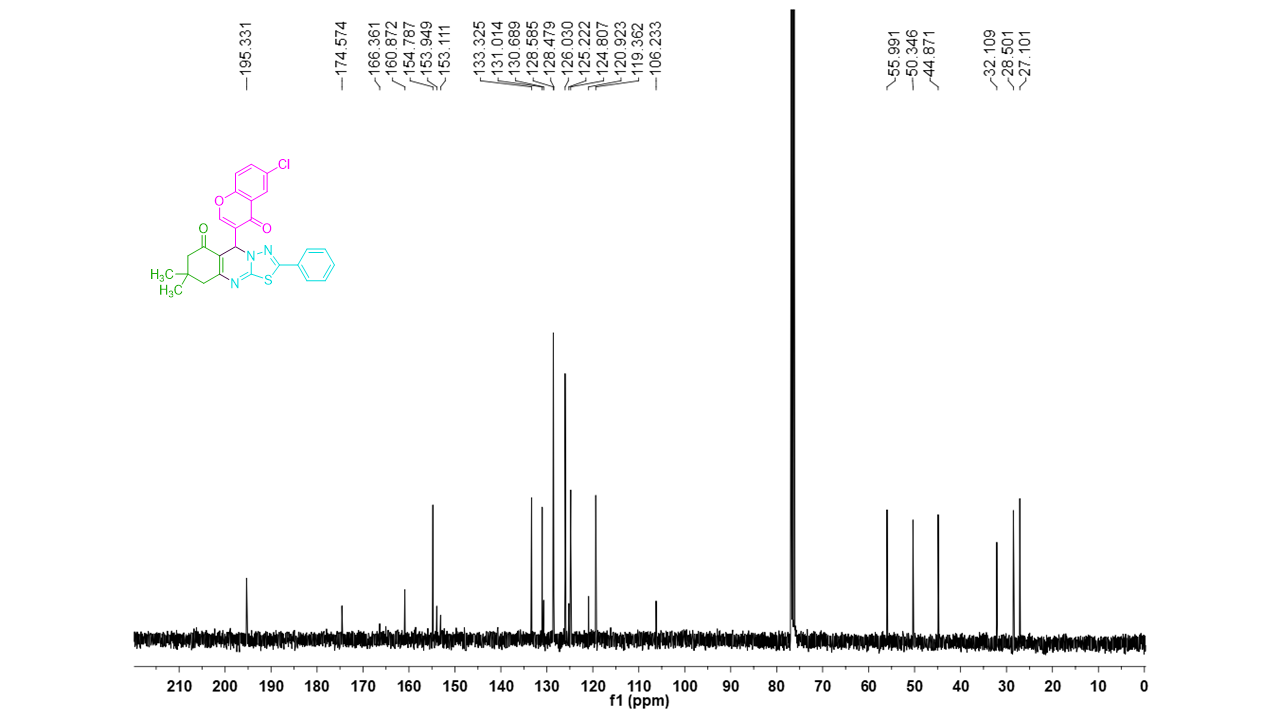
**

**Figure S41. ^13^C NMR (100 MHz, CDCl_3_) of 4t**

**
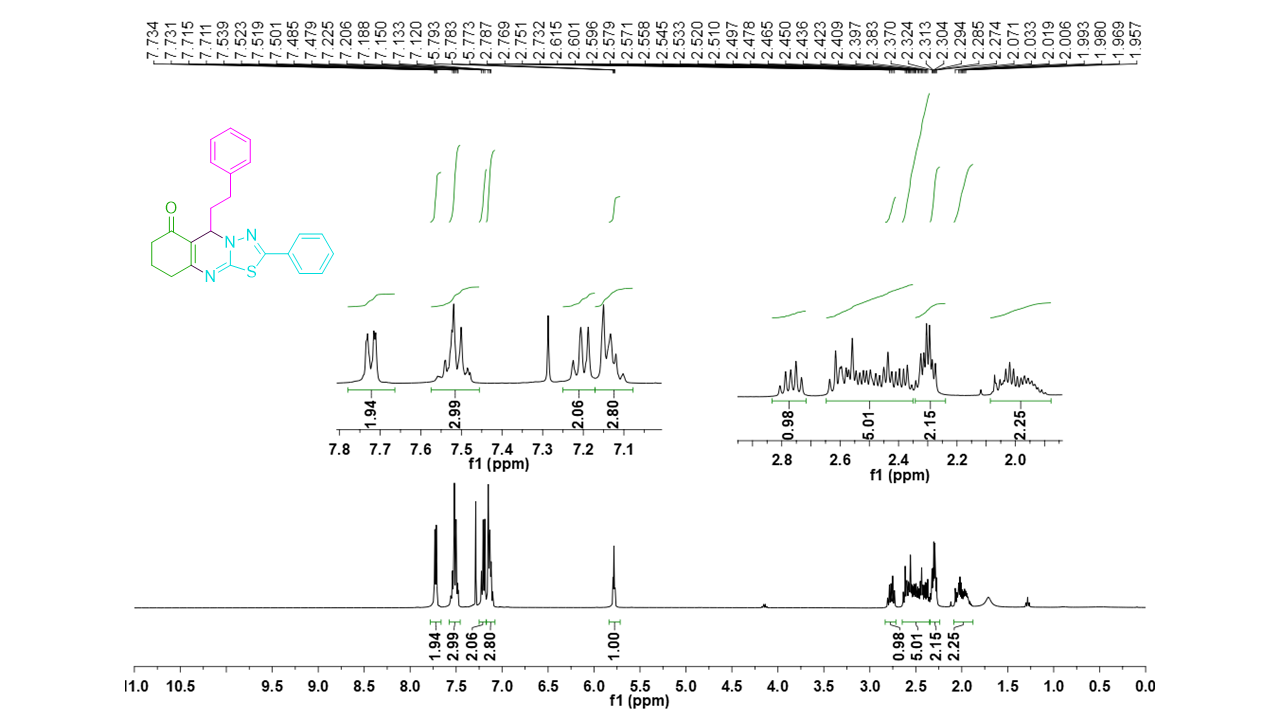
**

**Figure S42. ^1^H NMR (400 MHz, CDCl_3_) of 4u**

**
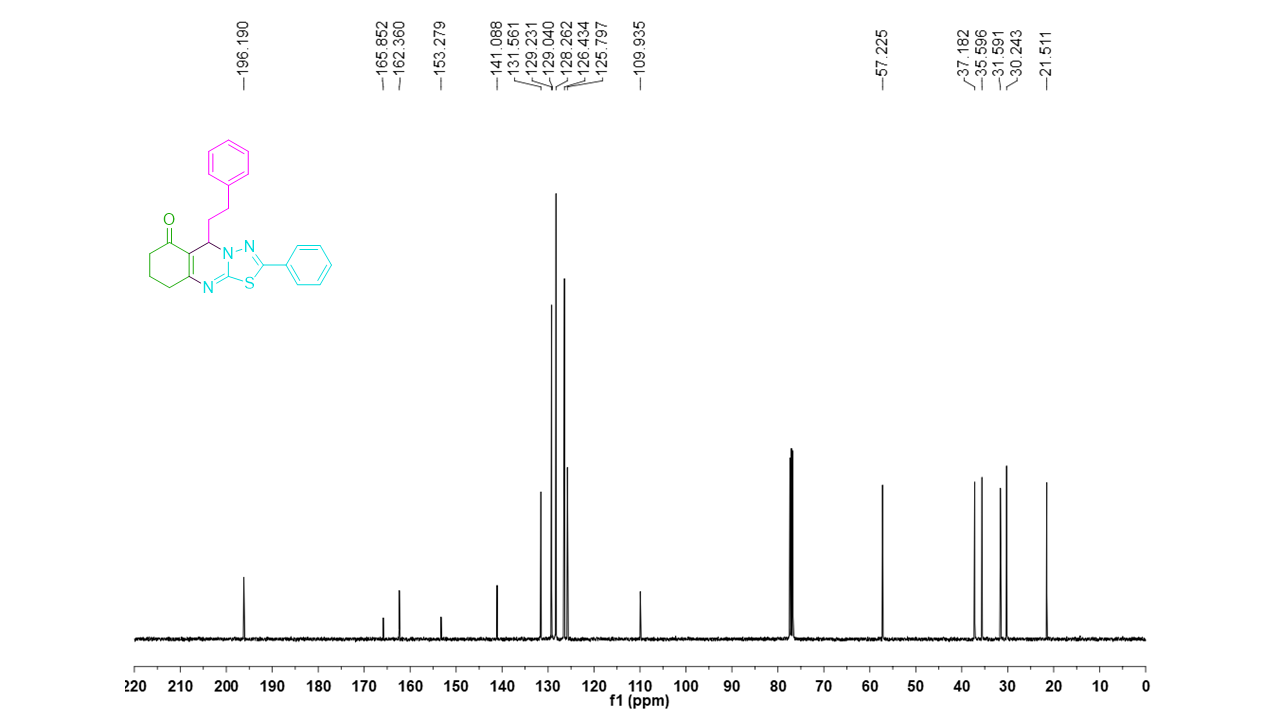
**

**Figure S43. ^13^C NMR (100 MHz, CDCl_3_) of 4u**

**
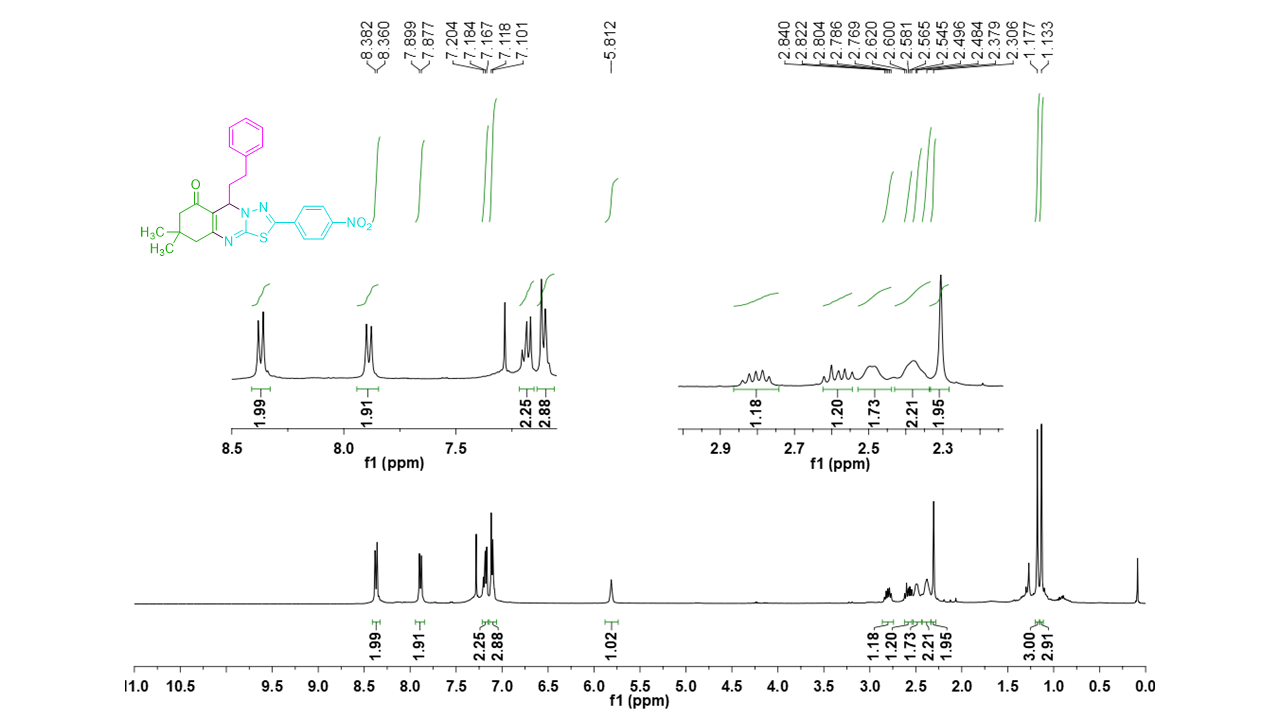
**

**Figure S44. ^1^H NMR (400 MHz, CDCl_3_) of 4v**

**
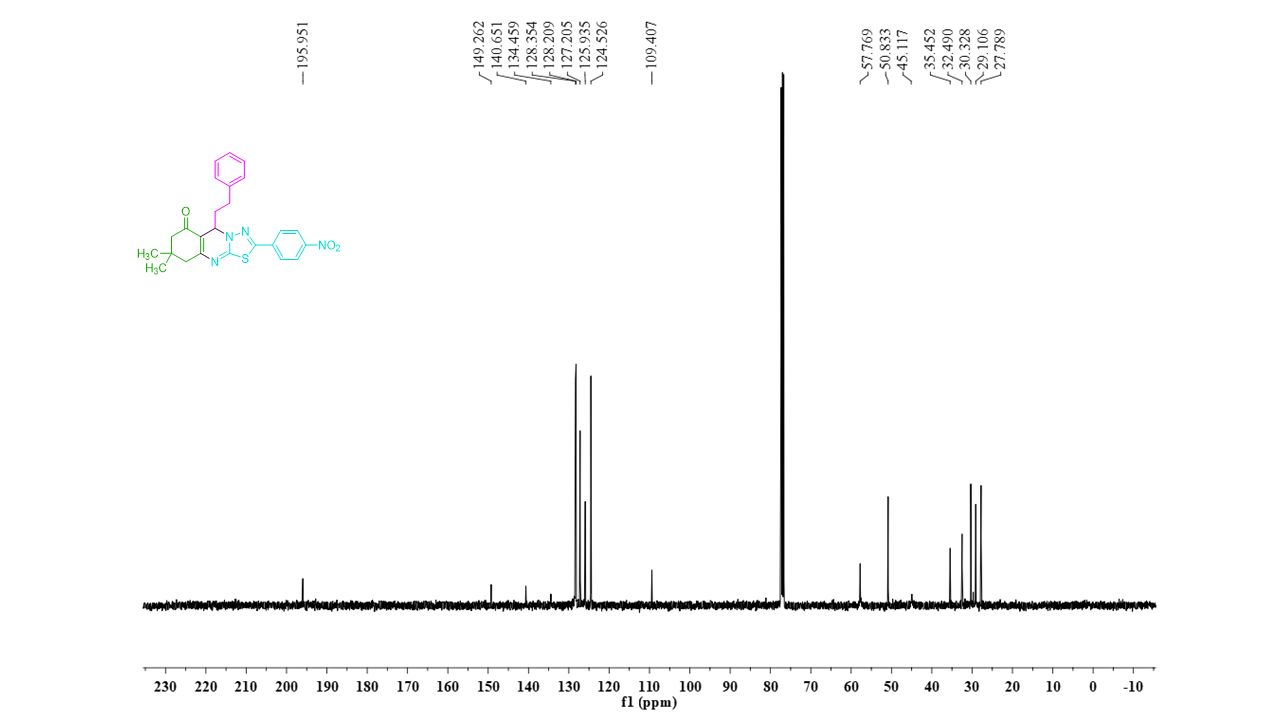
**

**Figure S45. ^13^C NMR (100 MHz, CDCl_3_) of 4v**

**
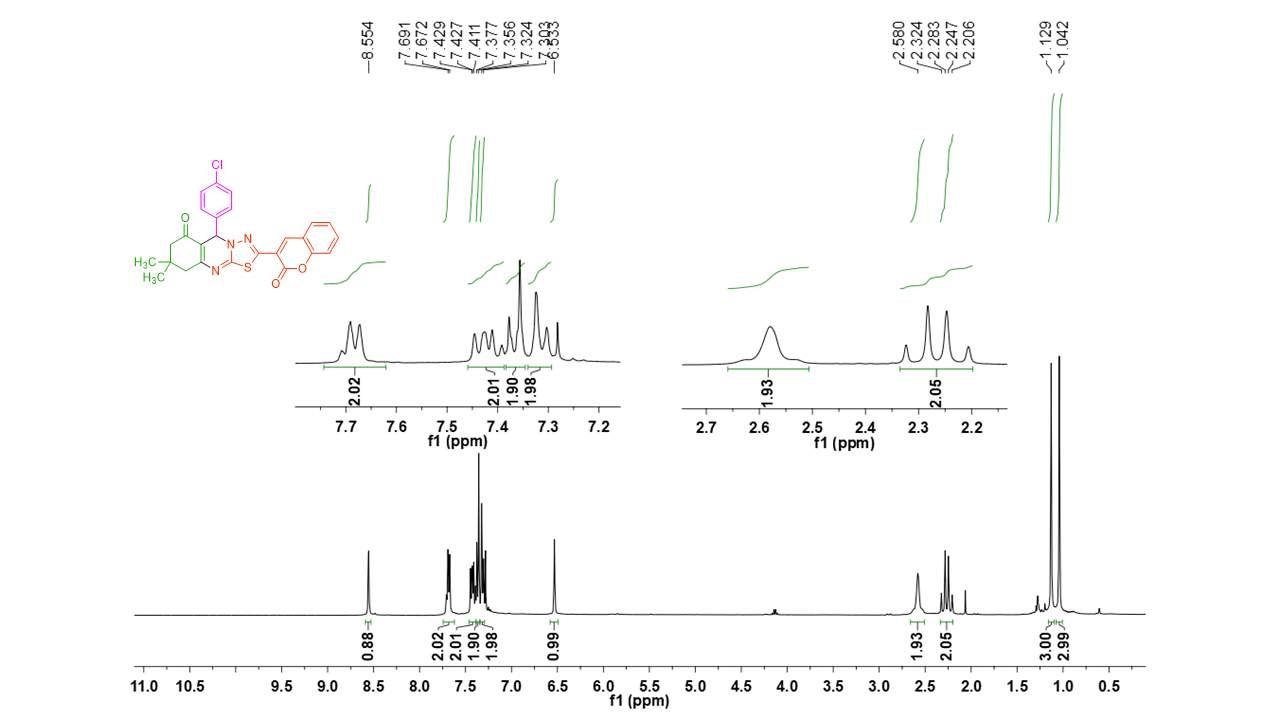
**

**Figure S46. ^1^H NMR (400 MHz, CDCl_3_) of 6a**

**
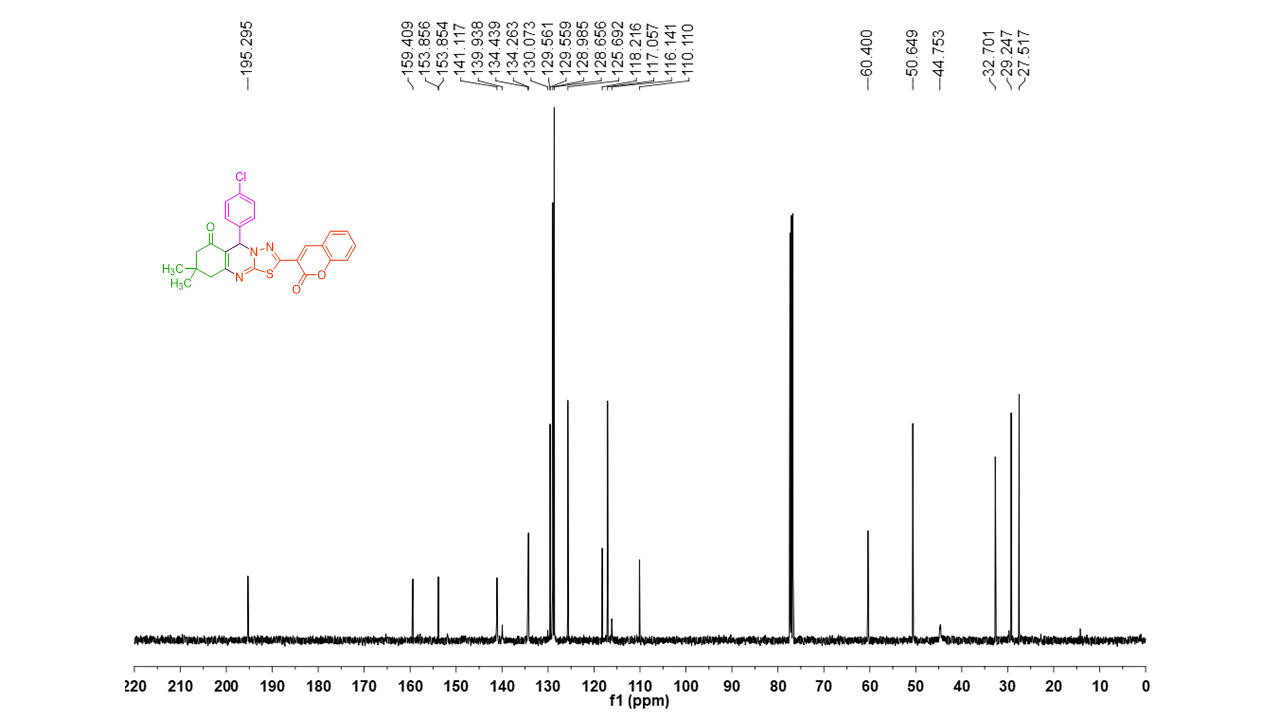
**

**Figure S47. ^13^C NMR (100 MHz, CDCl_3_) of 6a**

**
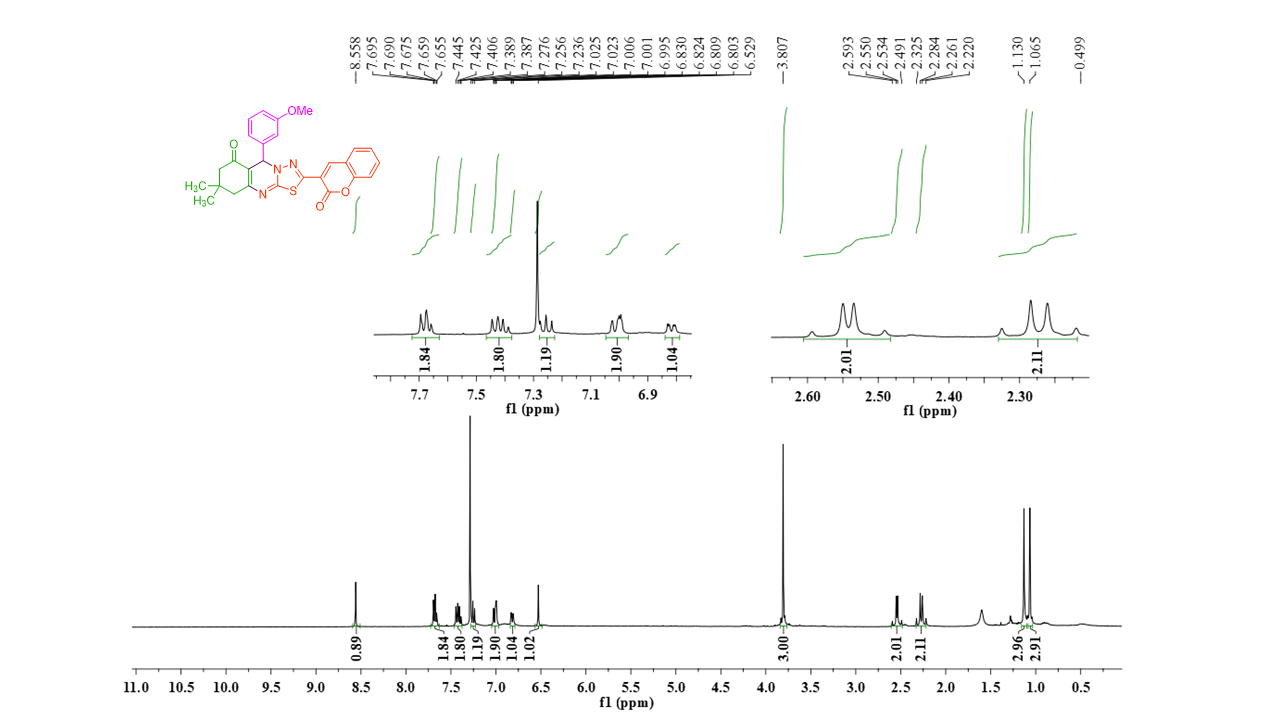
**

**Figure S48. ^1^H NMR (400 MHz, CDCl_3_) of 6b**

**
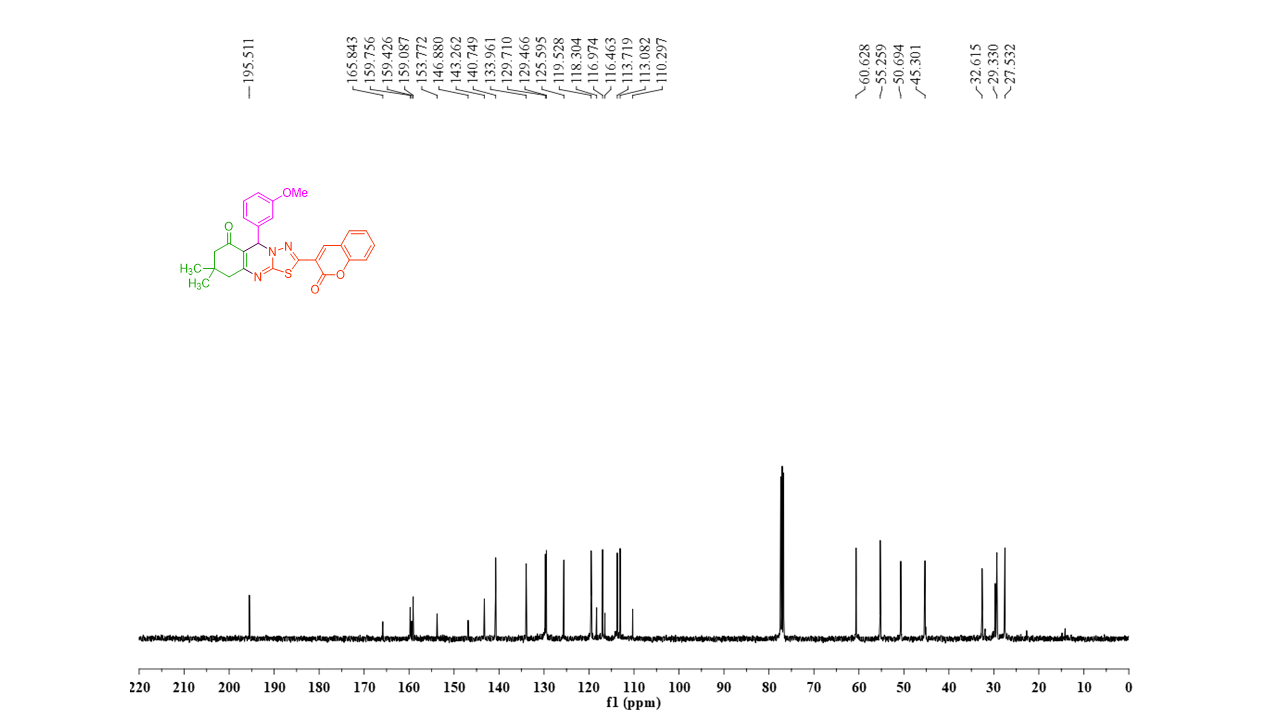
**

**Figure S49. ^13^C NMR (100 MHz, CDCl_3_) of 6b**

**
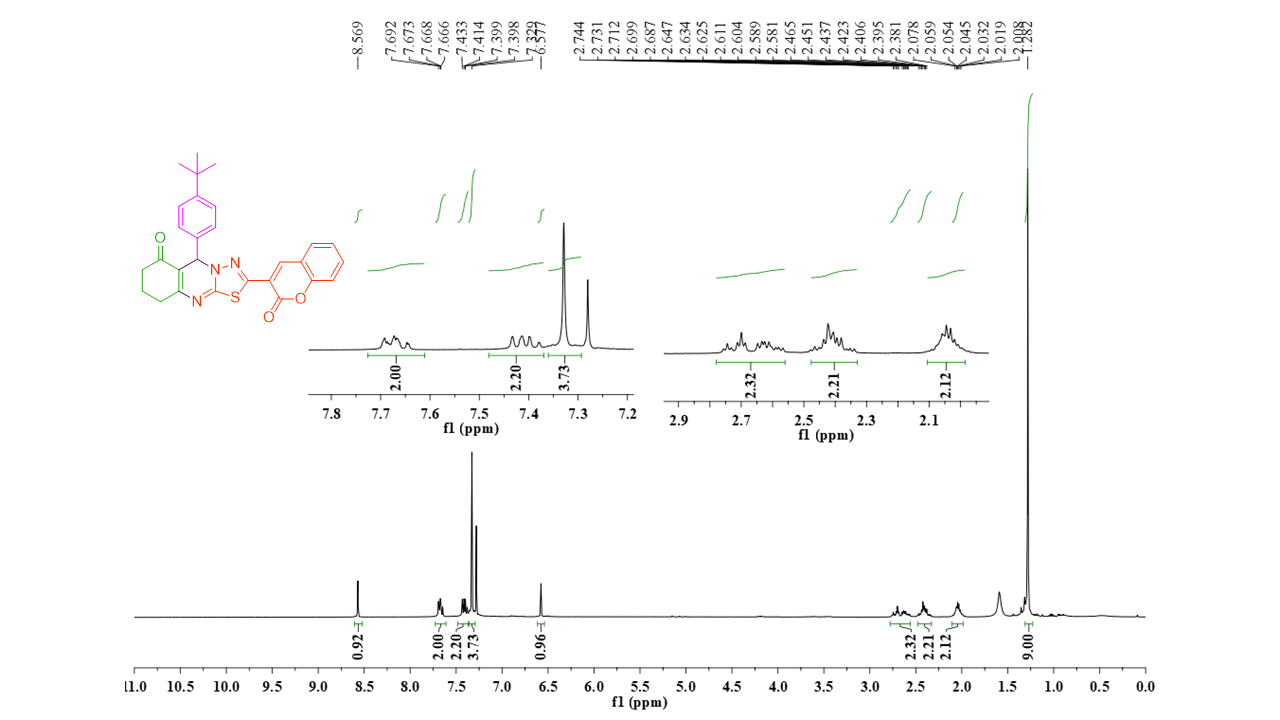
**

**Figure S50. ^1^H NMR (400 MHz, CDCl_3_) of 6c**

**
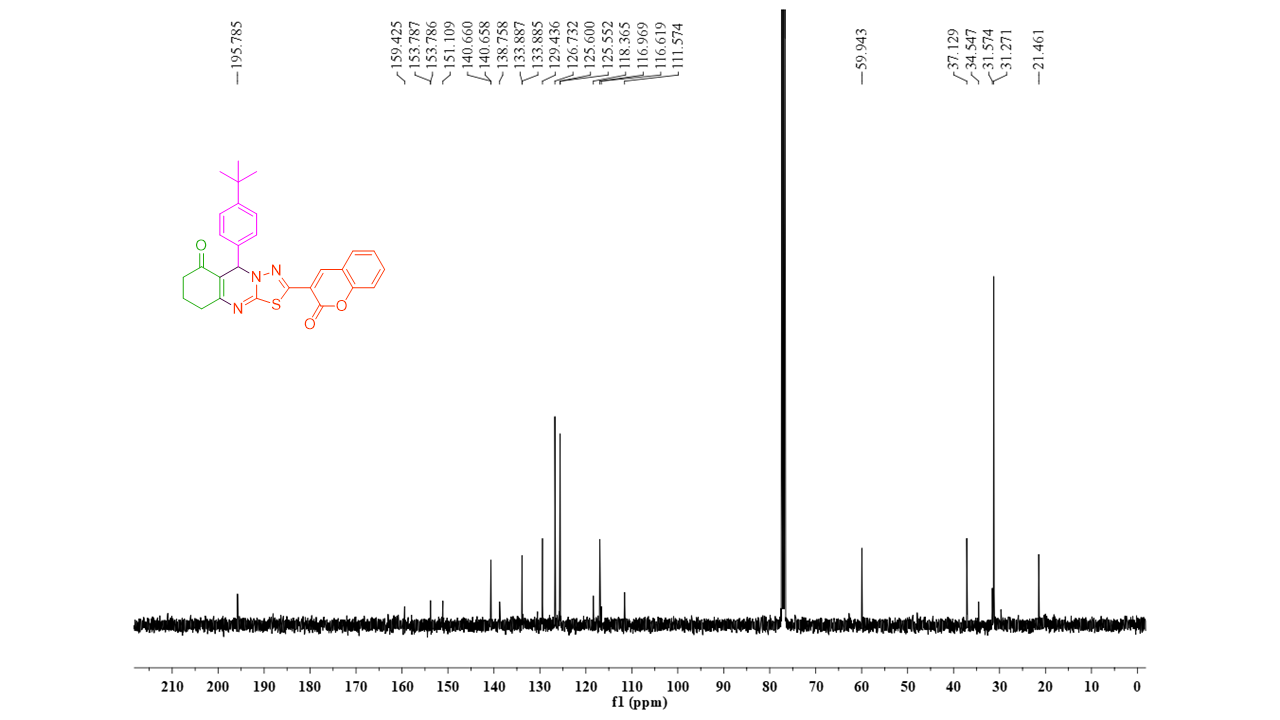
**

**Figure S51. ^13^C NMR (100 MHz, CDCl_3_) of 6c**

**
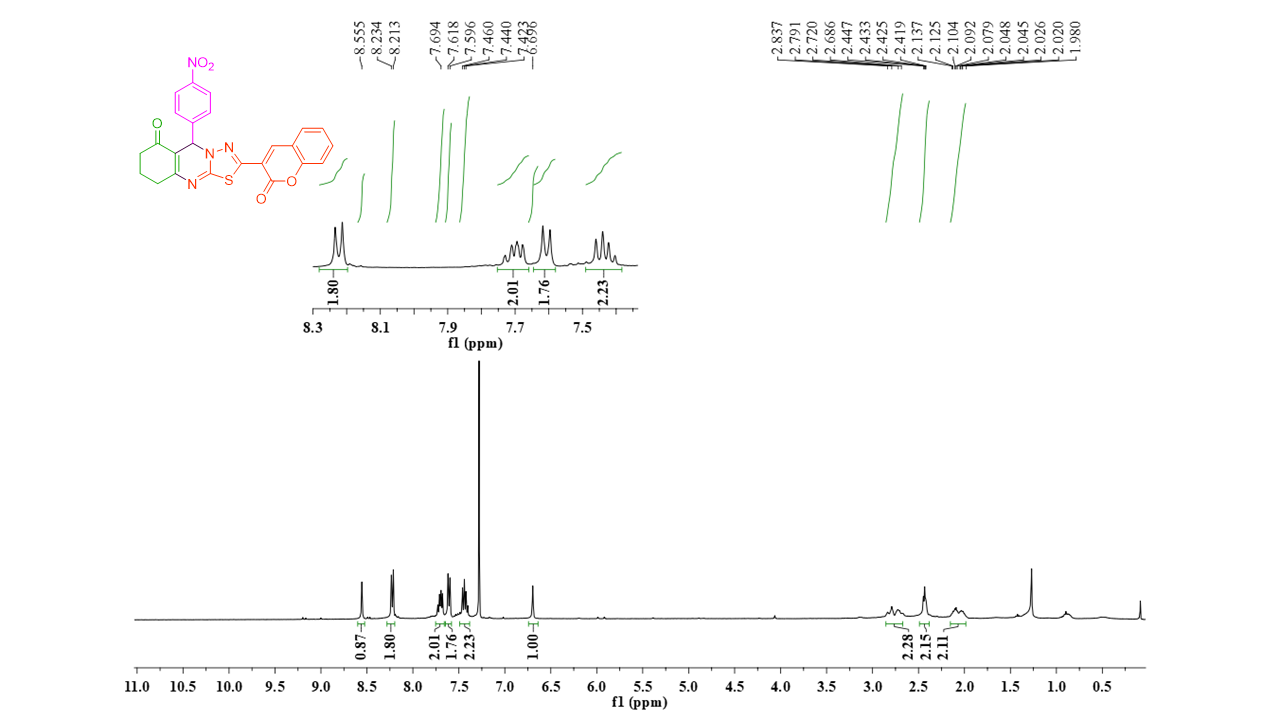
**

**Figure S52. ^1^H NMR (400 MHz, CDCl_3_) of 6d**

**
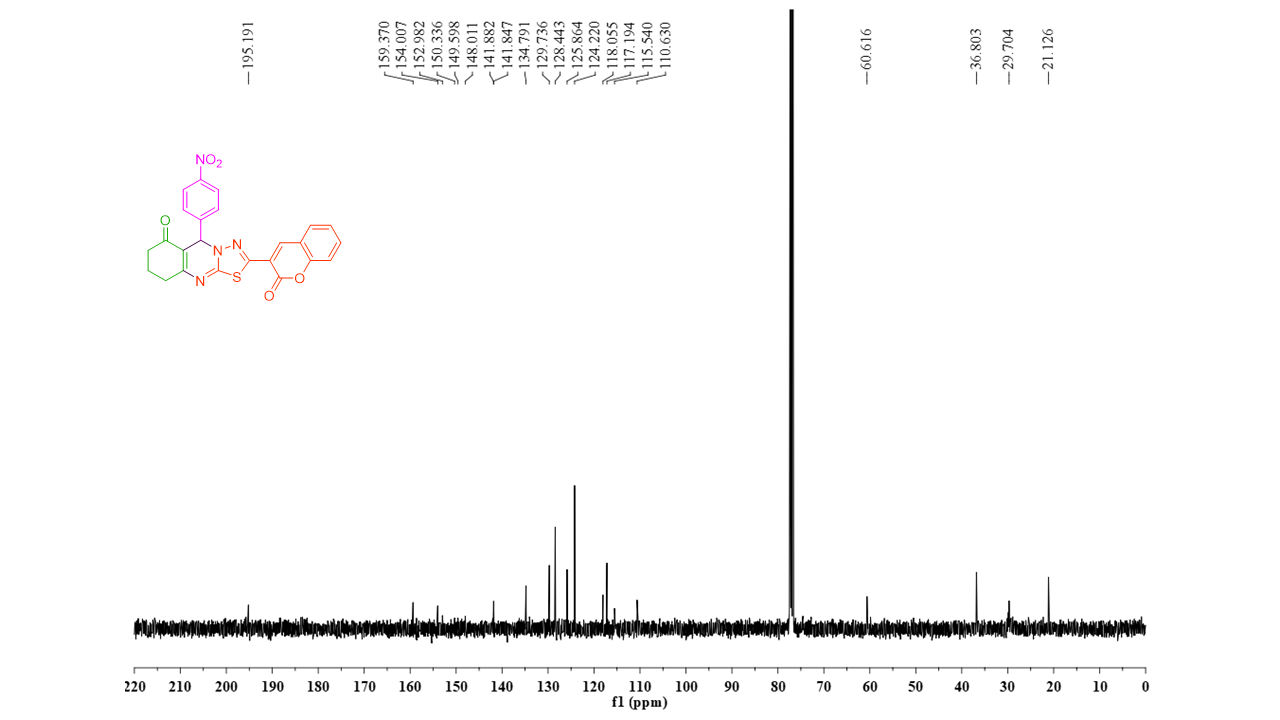
**

**Figure S53. ^13^C NMR (100 MHz, CDCl_3_) of 6d**

**
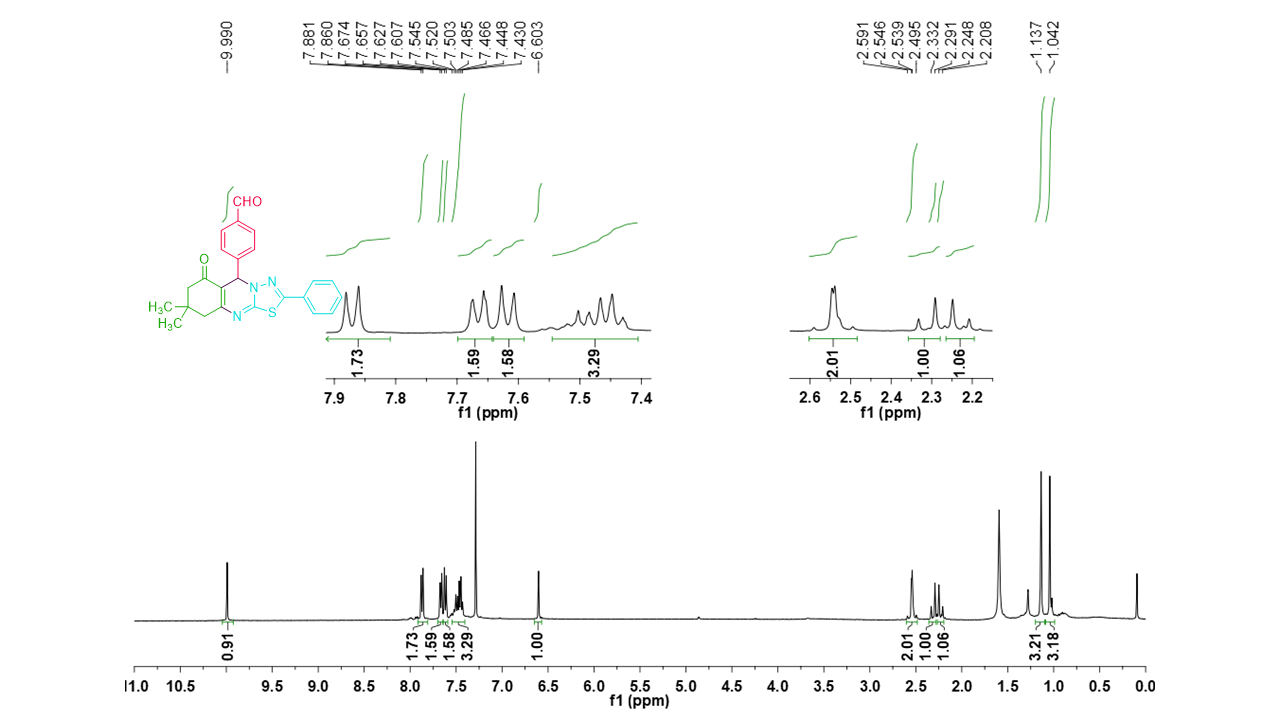
**

**Figure S54. ^1^H NMR (400 MHz, CDCl_3_) of 8a**

**
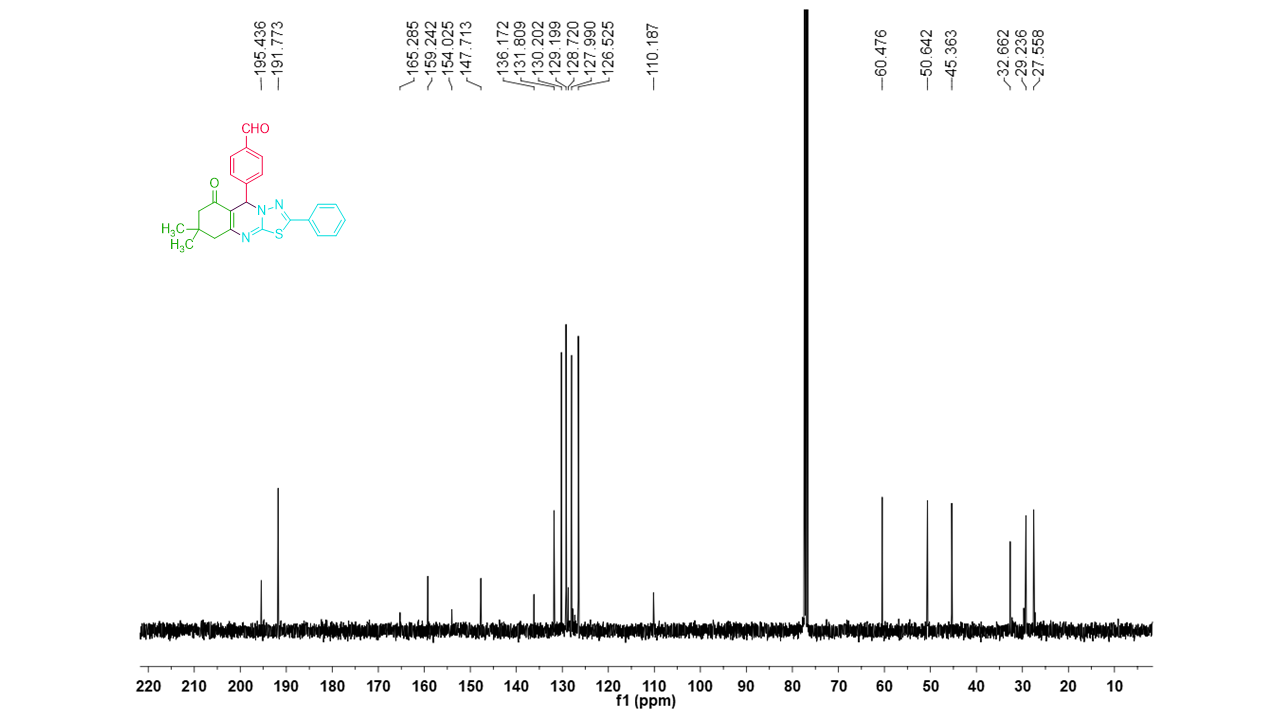
**

**Figure S55. ^13^C NMR (100 MHz, CDCl_3_) of 8a**

**
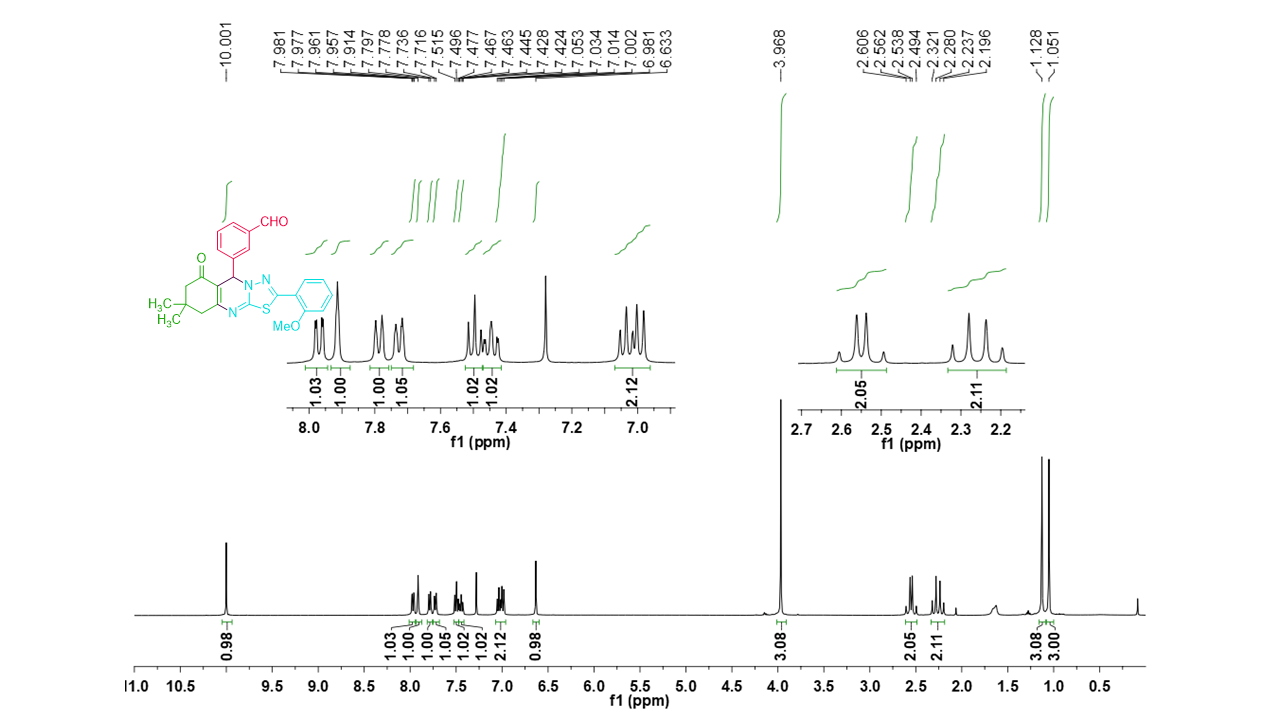
**

**Figure S56. ^1^H NMR (400 MHz, CDCl_3_) of 8b**

**
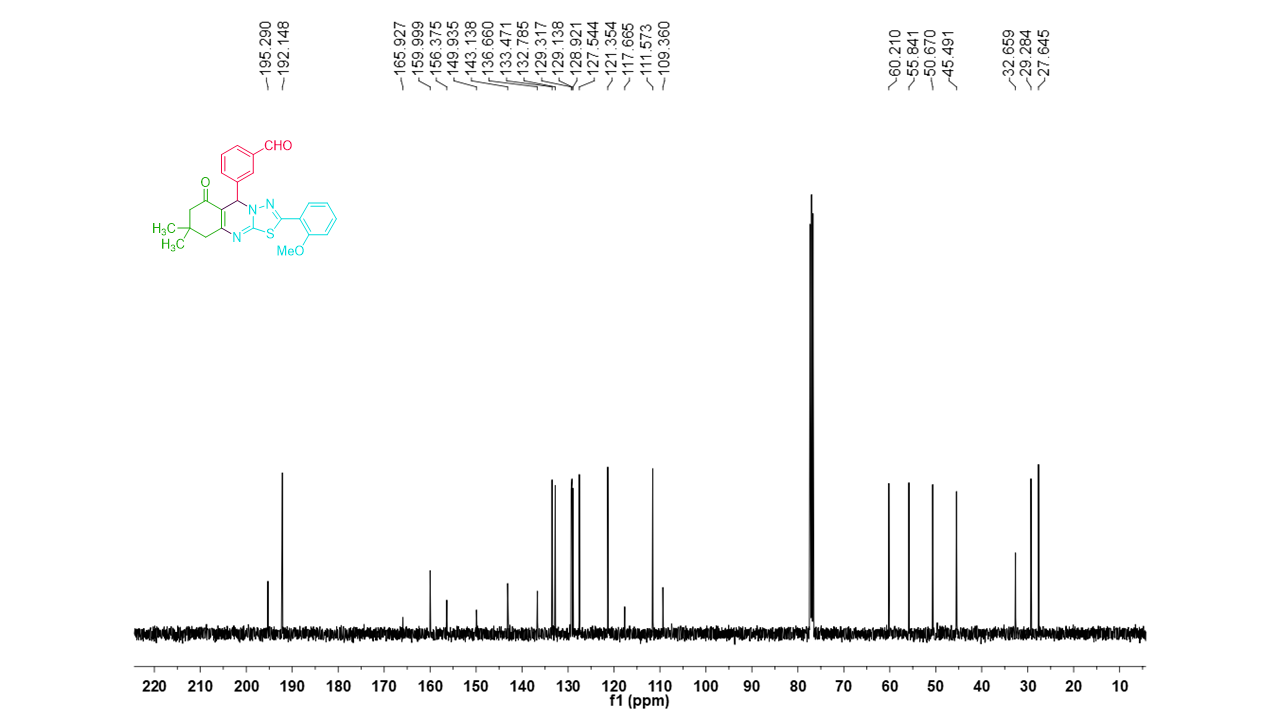
**

**Figure S57. ^13^C NMR (100 MHz, CDCl_3_) of 8b**

**
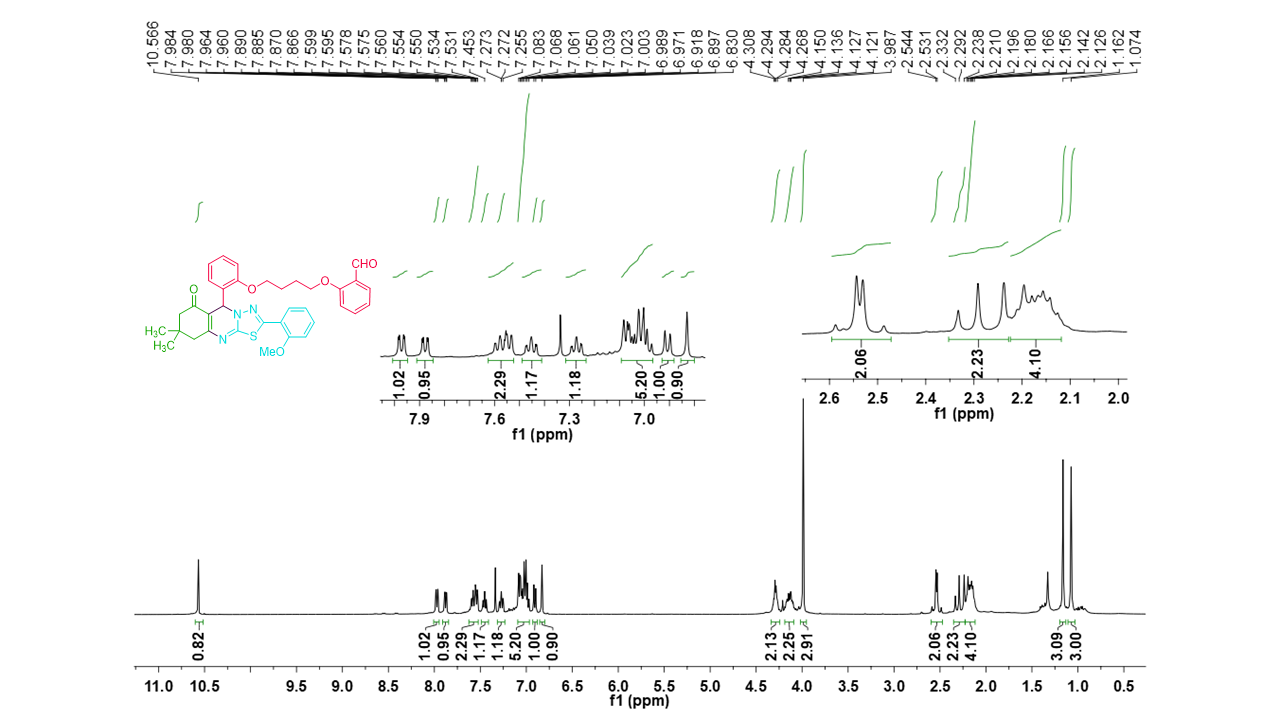
**

**Figure S58. ^1^H NMR (400 MHz, CDCl_3_) of 8c**

**
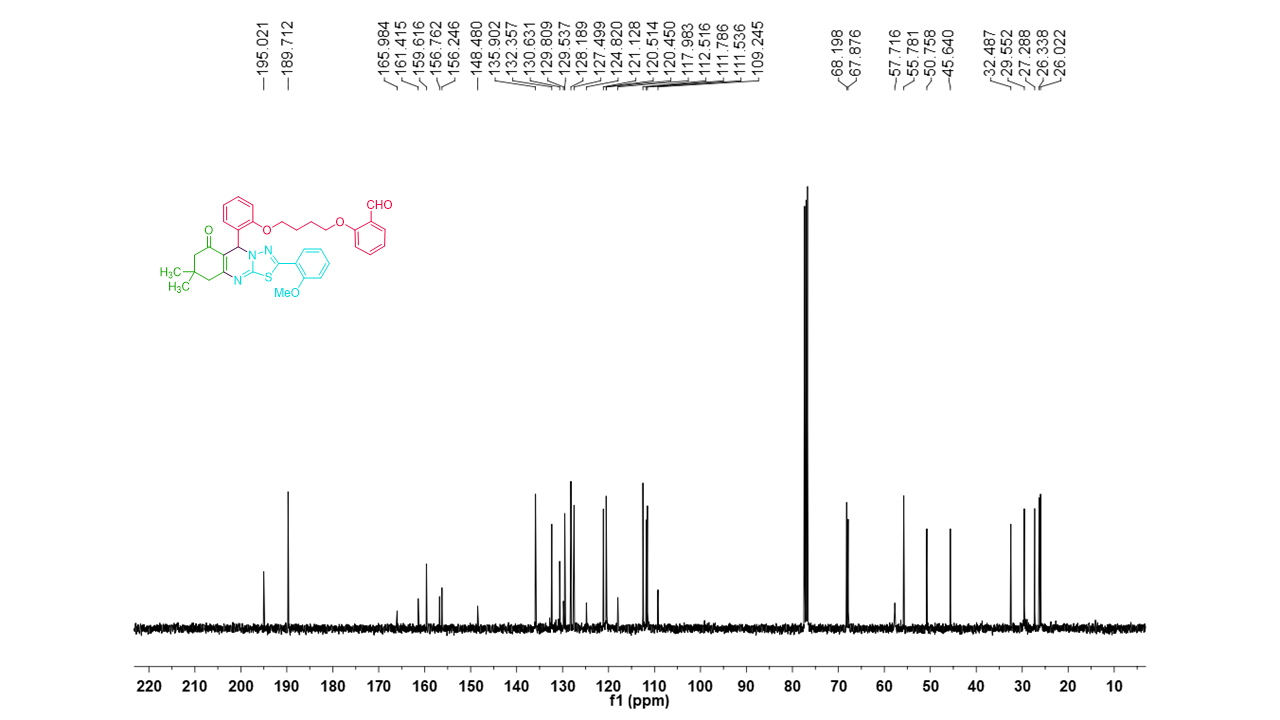
**

**Figure S59. ^13^C NMR (100 MHz, CDCl_3_) of 8c**

**
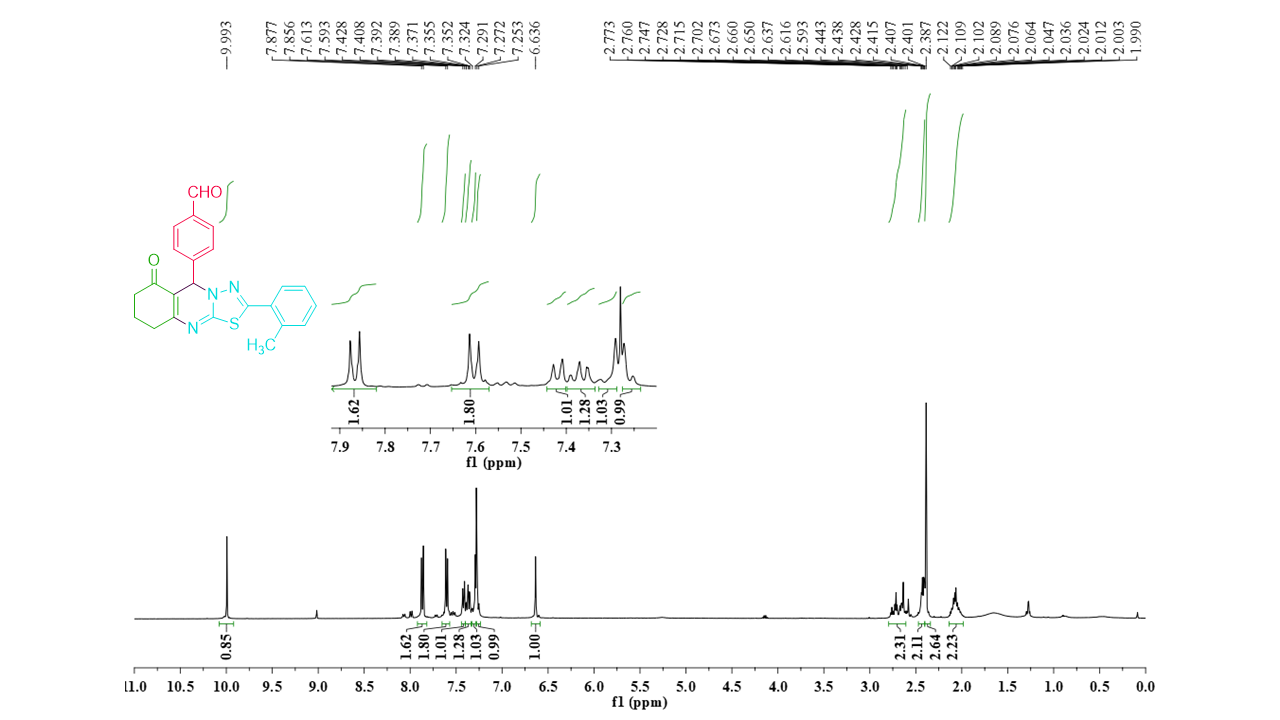
**

**Figure S60. ^1^H NMR (400 MHz, CDCl_3_) of 8d**

**
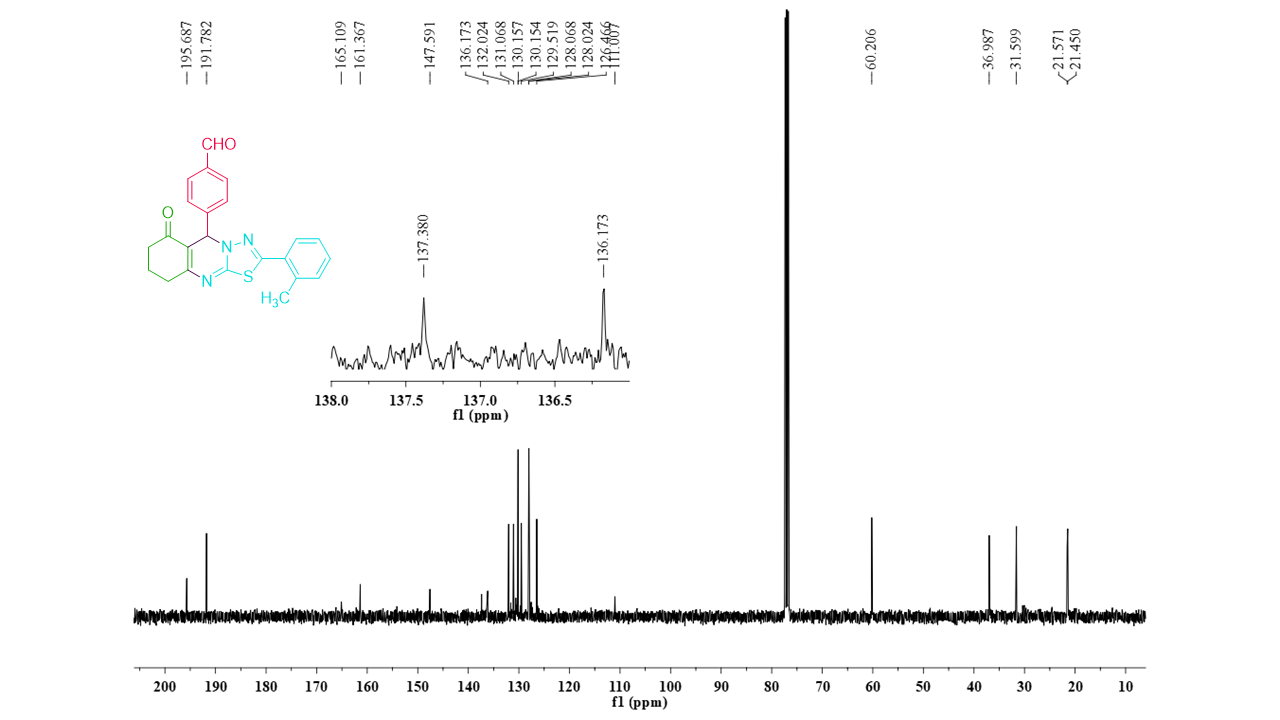
**

**Figure S61. ^13^C NMR (100 MHz, CDCl_3_) of 8d**

**
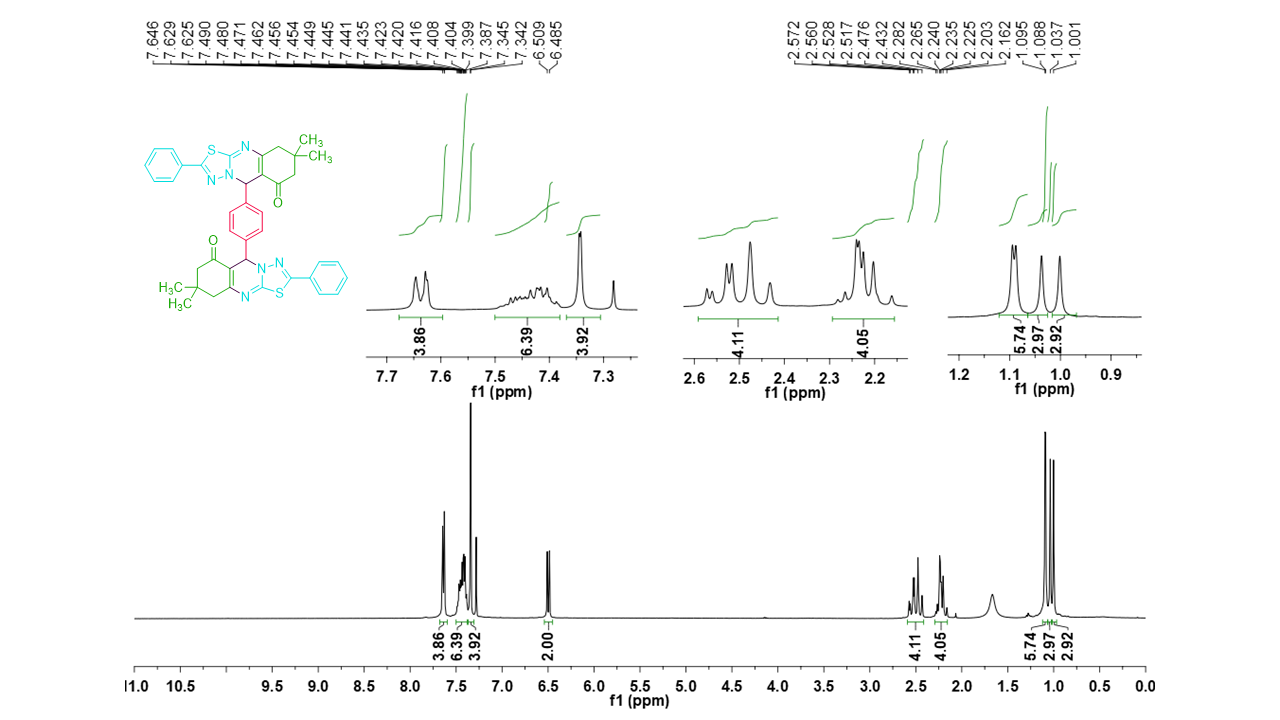
**

**Figure S62. ^1^H NMR (400 MHz, CDCl_3_) of 9a**

**
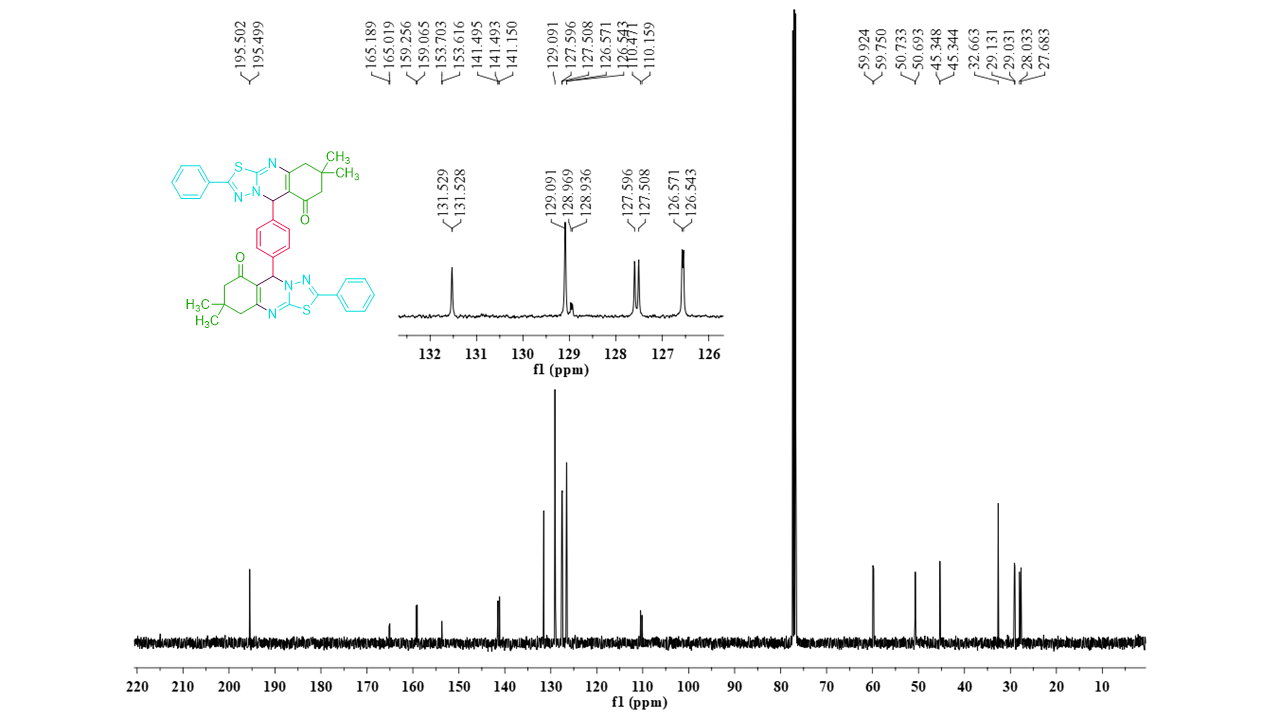
**

**Figure S63. ^13^C NMR (100 MHz, CDCl_3_) of 9a**

**
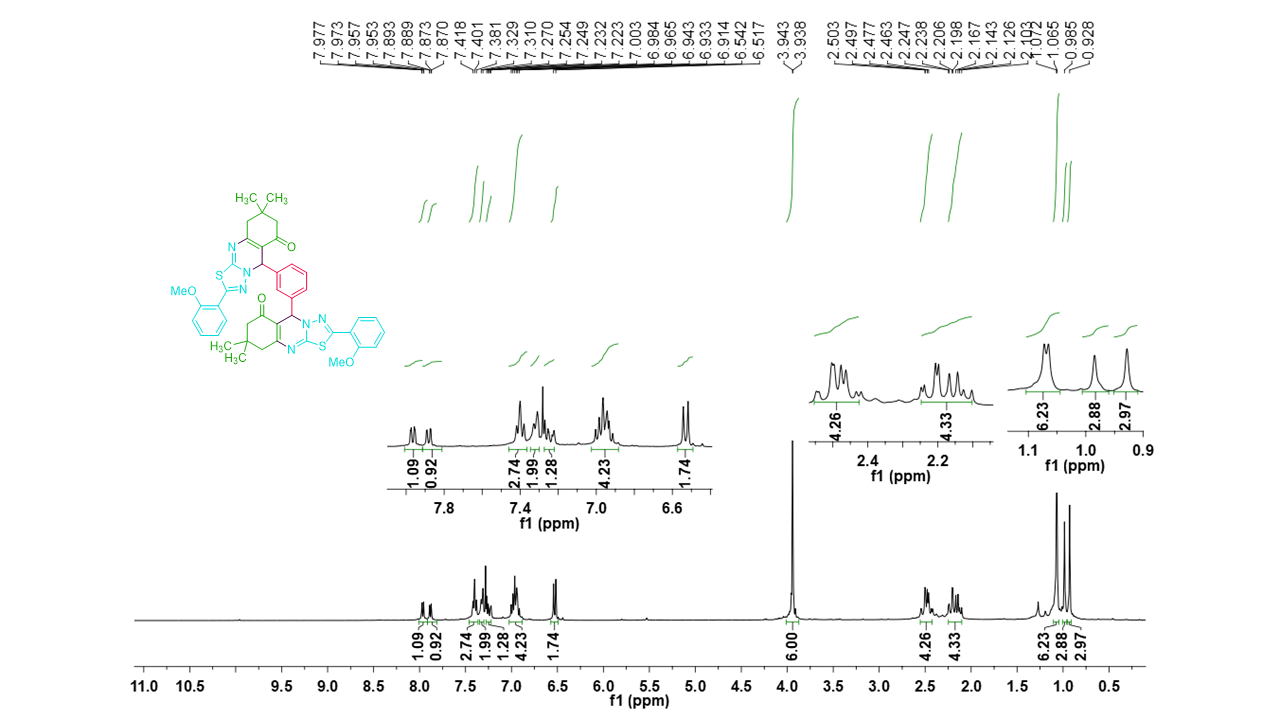
**

**Figure S64. ^1^H NMR (400 MHz, CDCl_3_) of 9b**

**
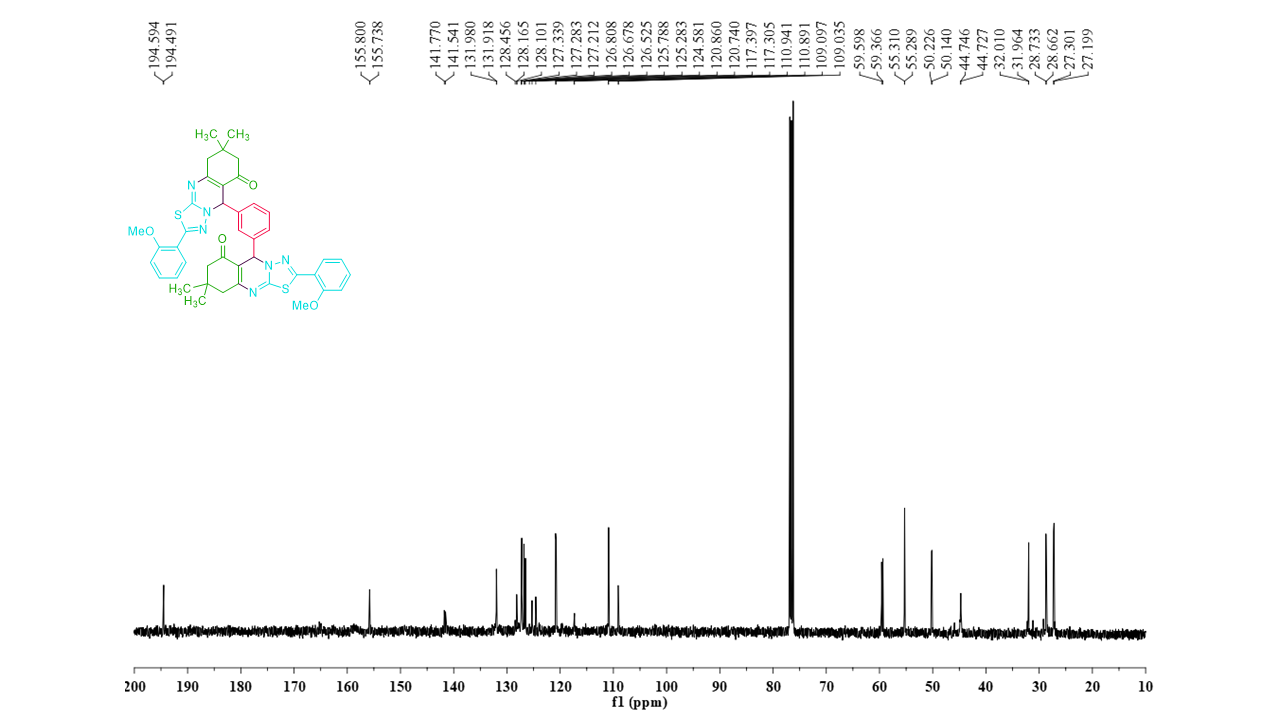
**

**Figure S65. ^13^C NMR (100 MHz, CDCl_3_) of 9b**

**
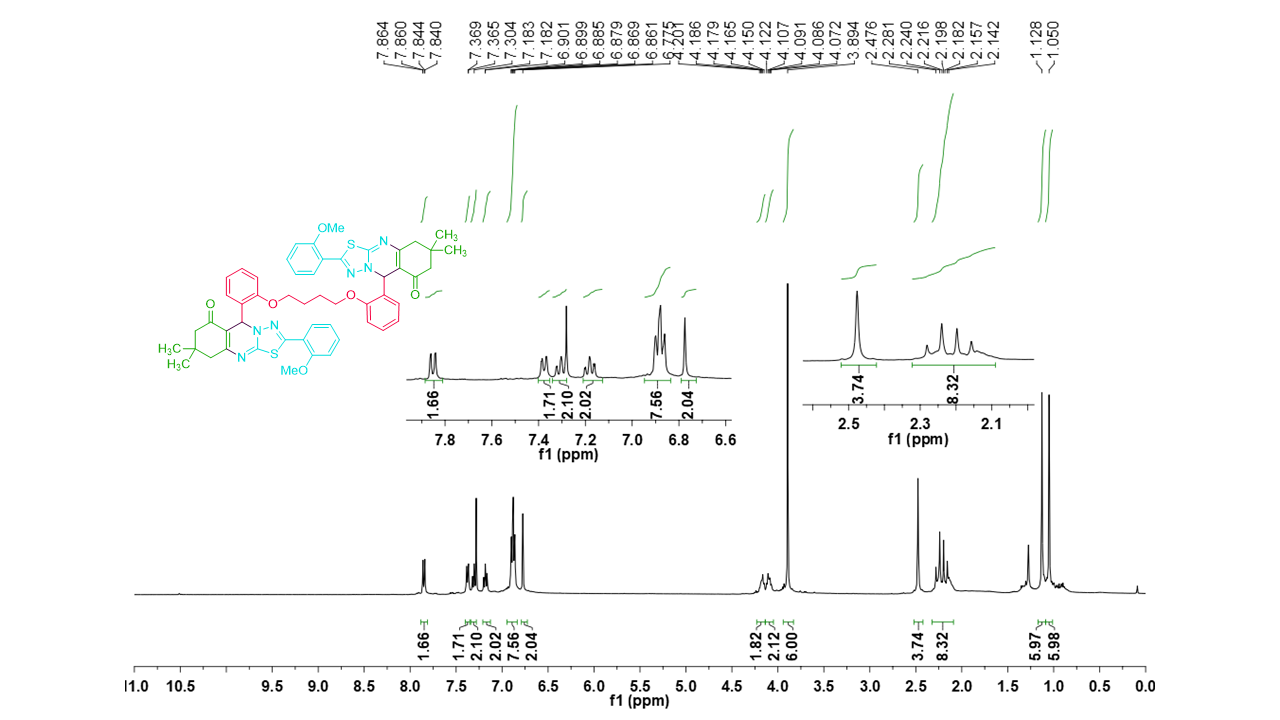
**

**Figure S66. ^1^H NMR (400 MHz, CDCl_3_) of 9c**

**
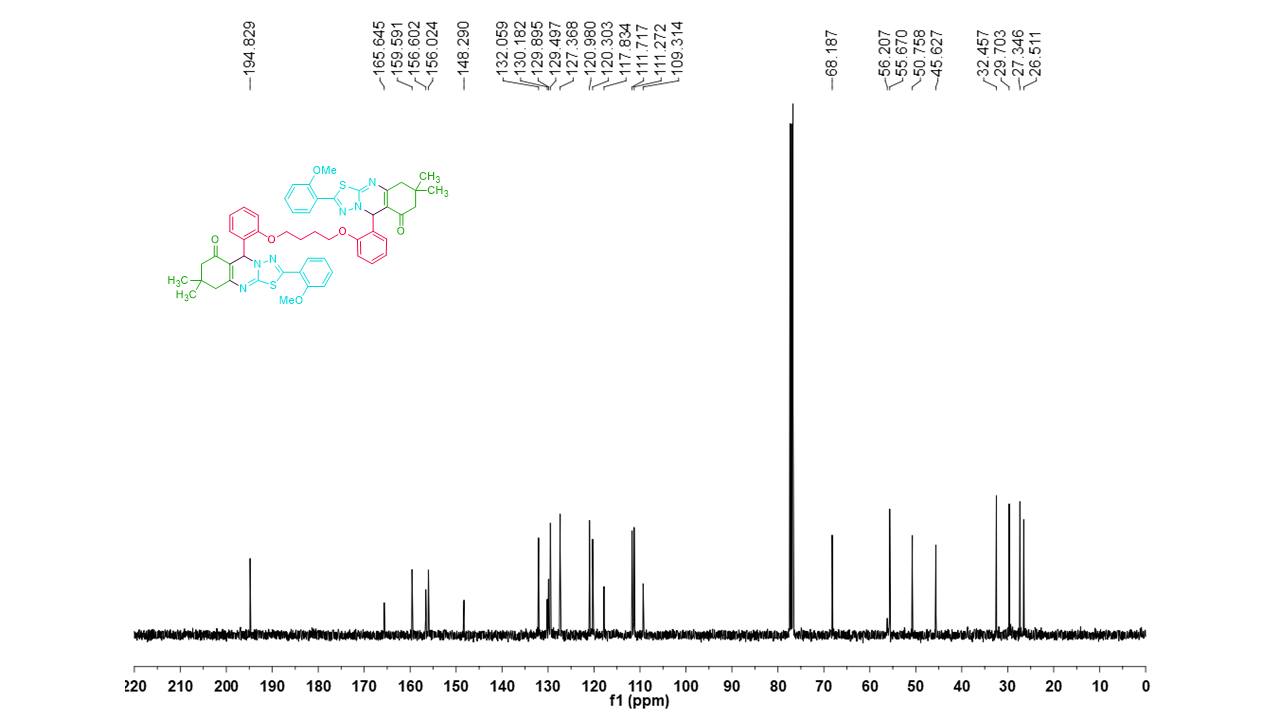
**

**Figure S67. ^13^C NMR (100 MHz, CDCl_3_) of 9c**

**
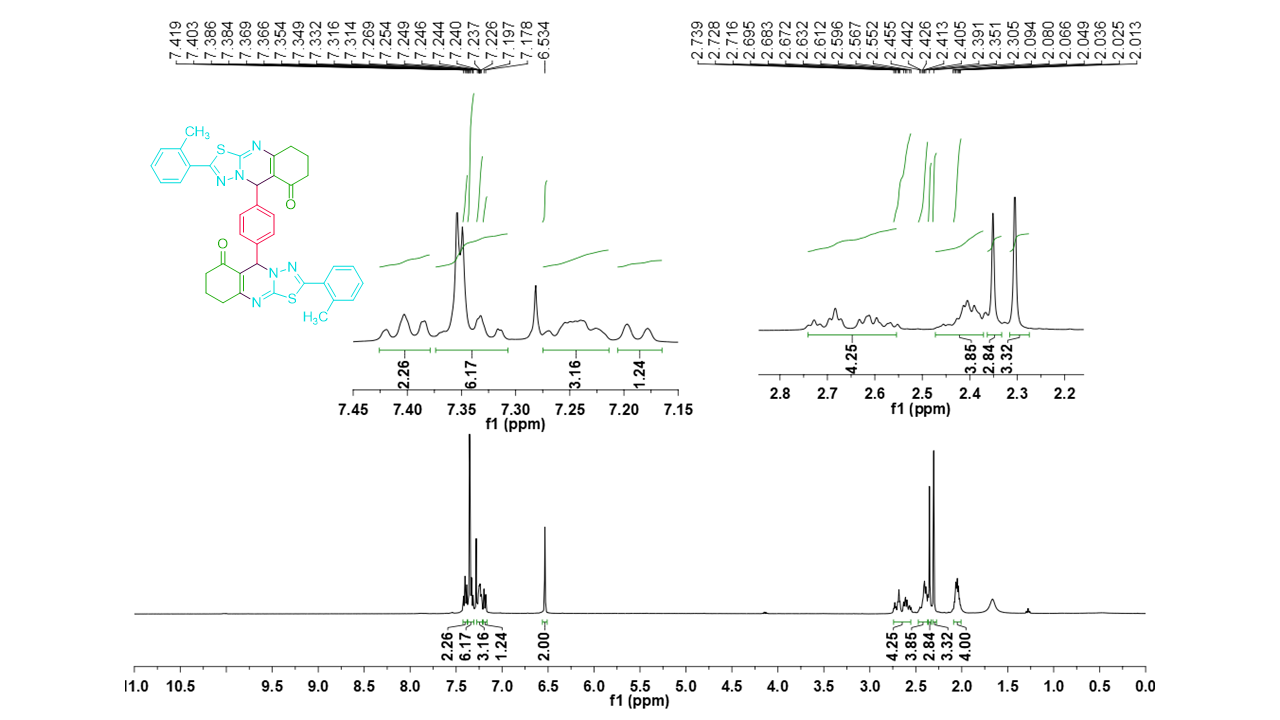
**

**Figure S68. ^1^H NMR (400 MHz, CDCl_3_) of 9d**

**
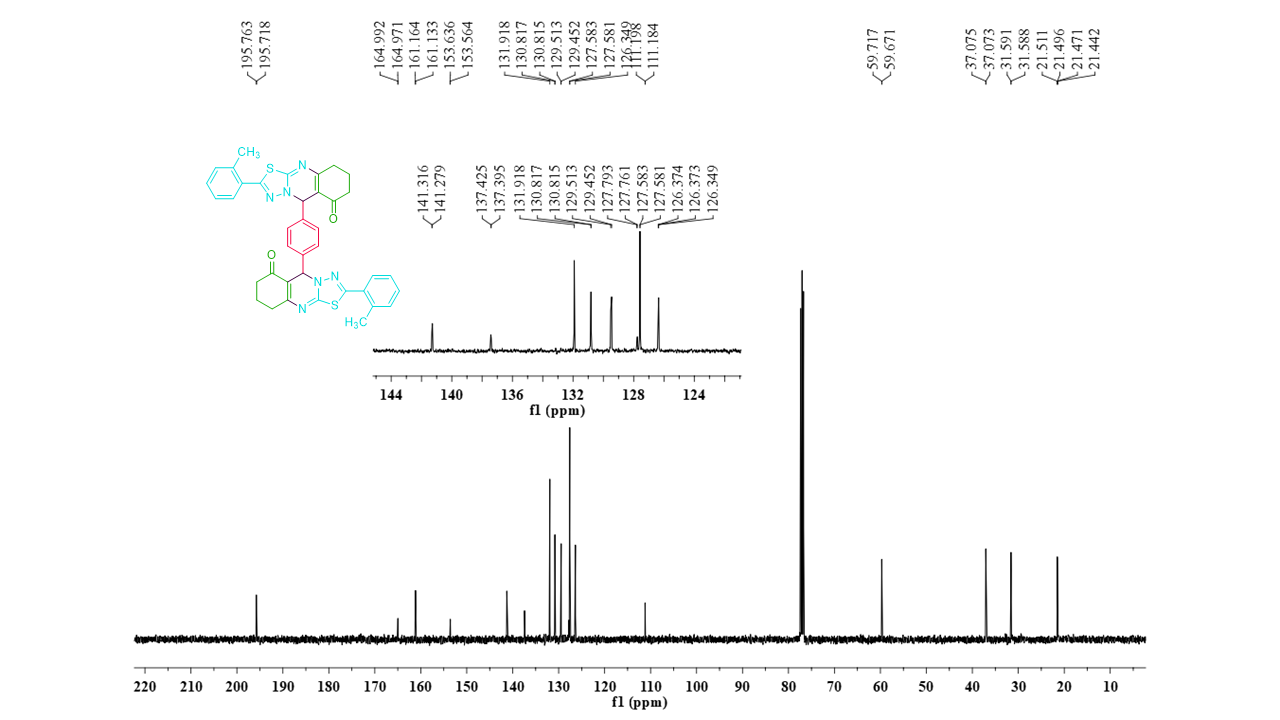
**

**Figure S69. ^13^C NMR (100 MHz, CDCl_3_) of 9d**

**
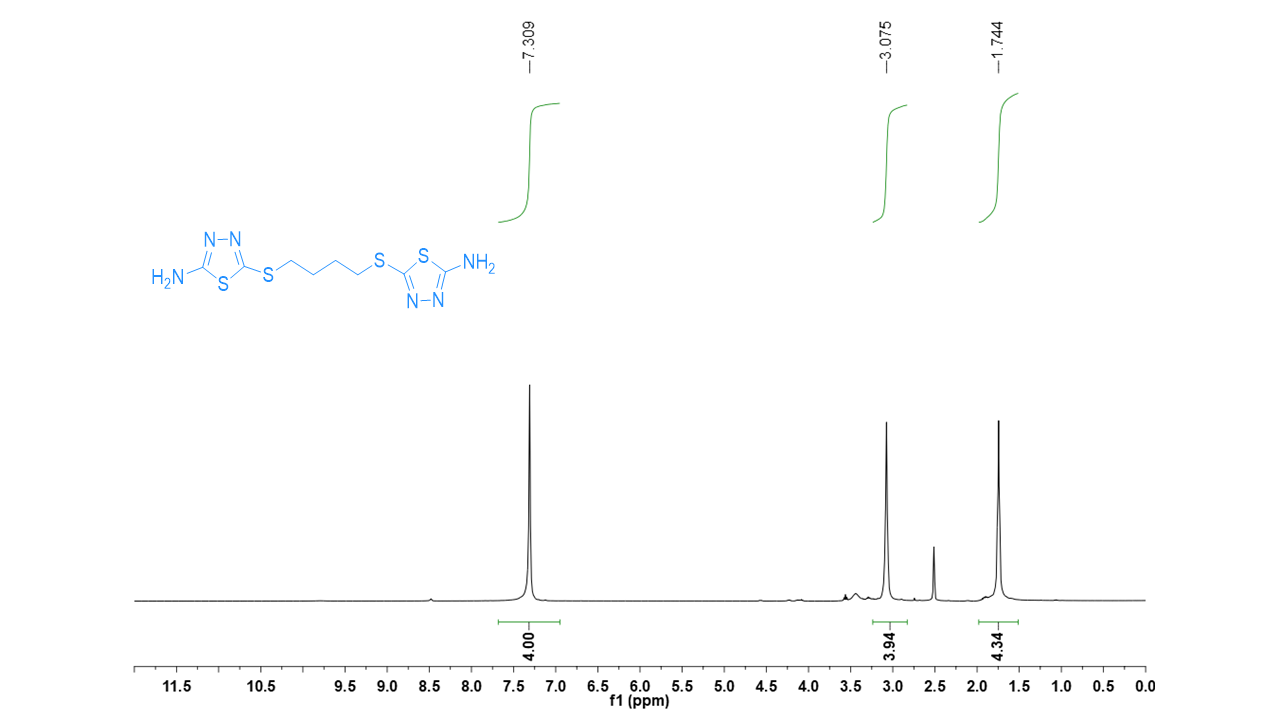
**

**Figure S70. ^1^H NMR (400 MHz, DMSO-*d_6_*) of 10**

**
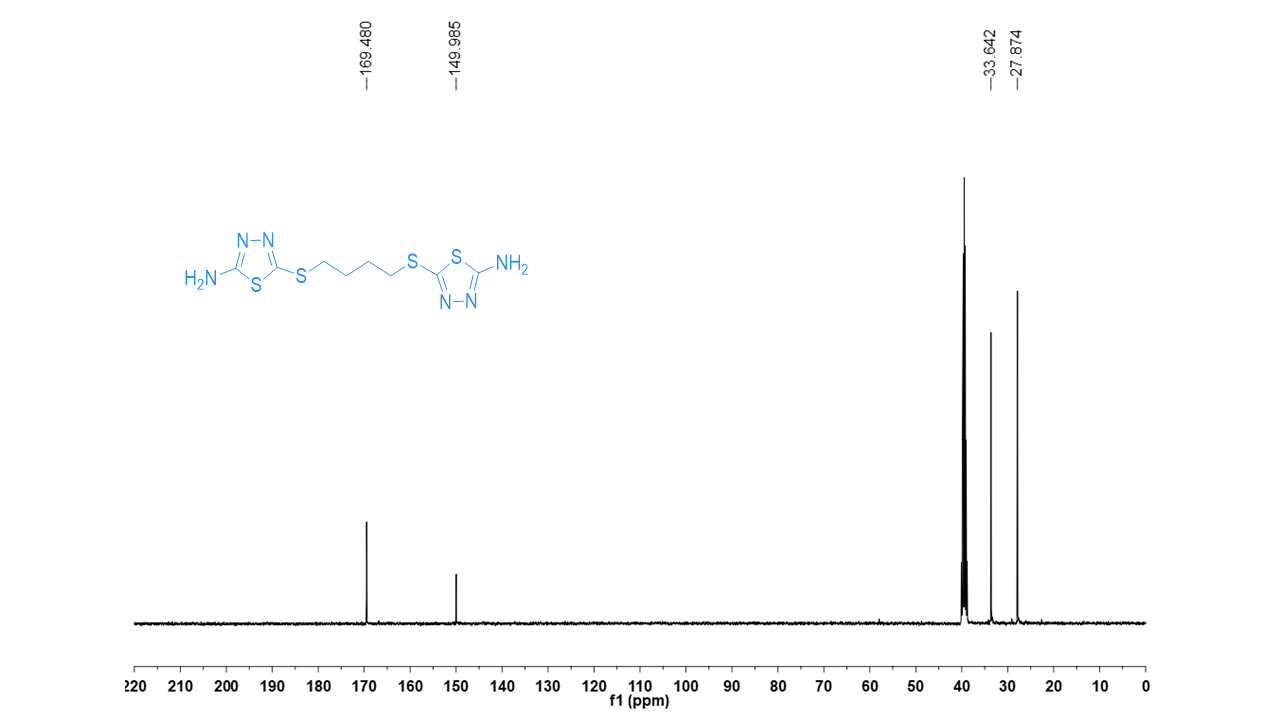
**

**Figure S71. ^13^C NMR (100 MHz, DMSO-*d_6_*) of 10**

**
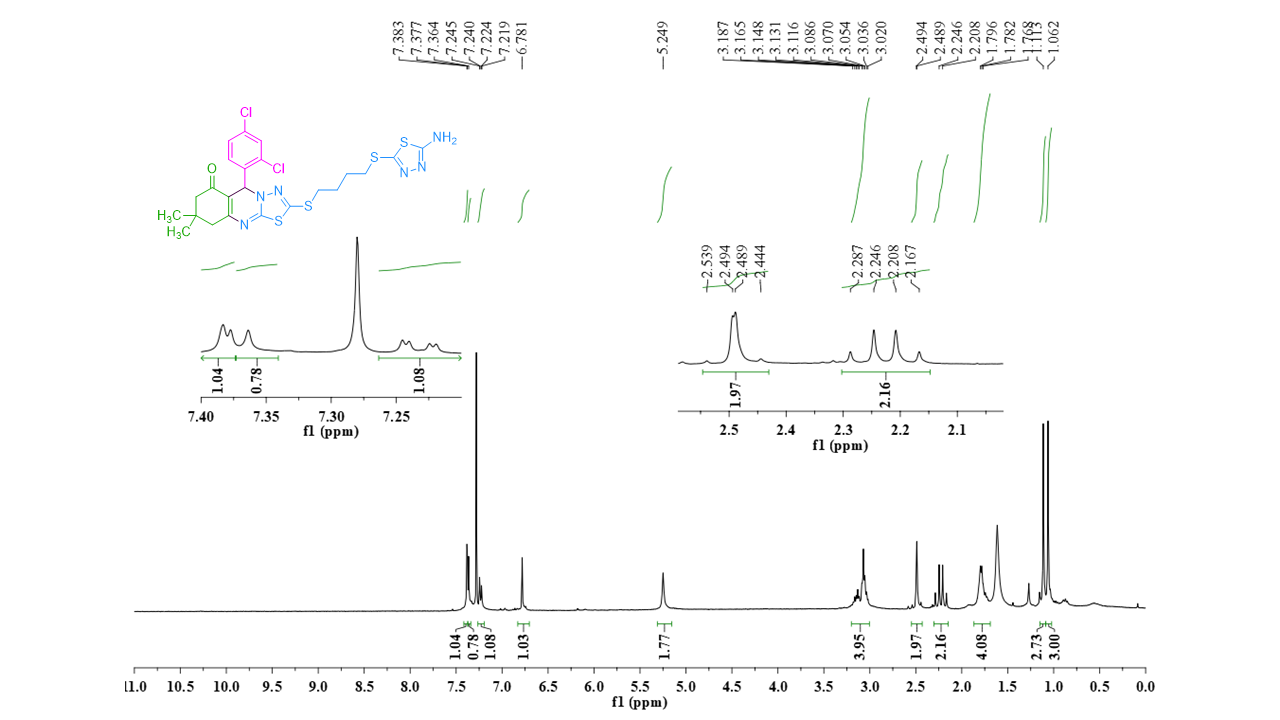
**

**Figure S72. ^1^H NMR (400 MHz, CDCl_3_) of 11a**

**
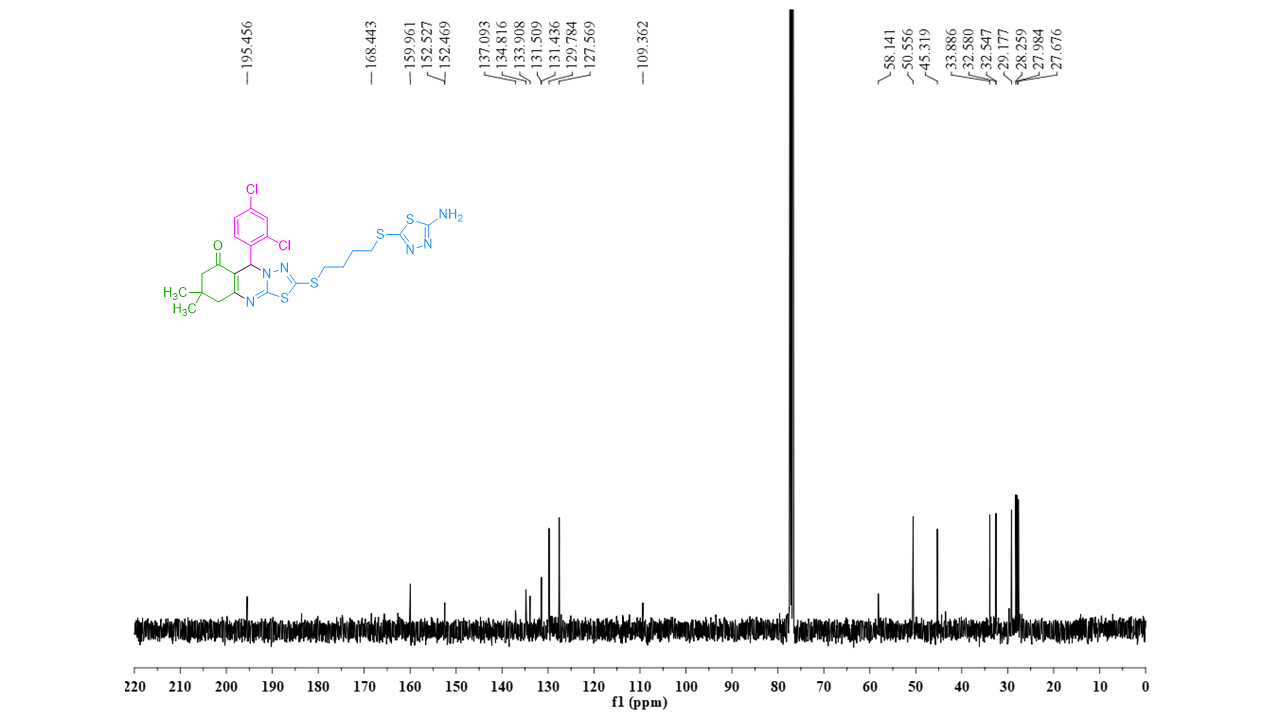
**

**Figure S73. ^13^C NMR (100 MHz, CDCl_3_) of 11a**

**
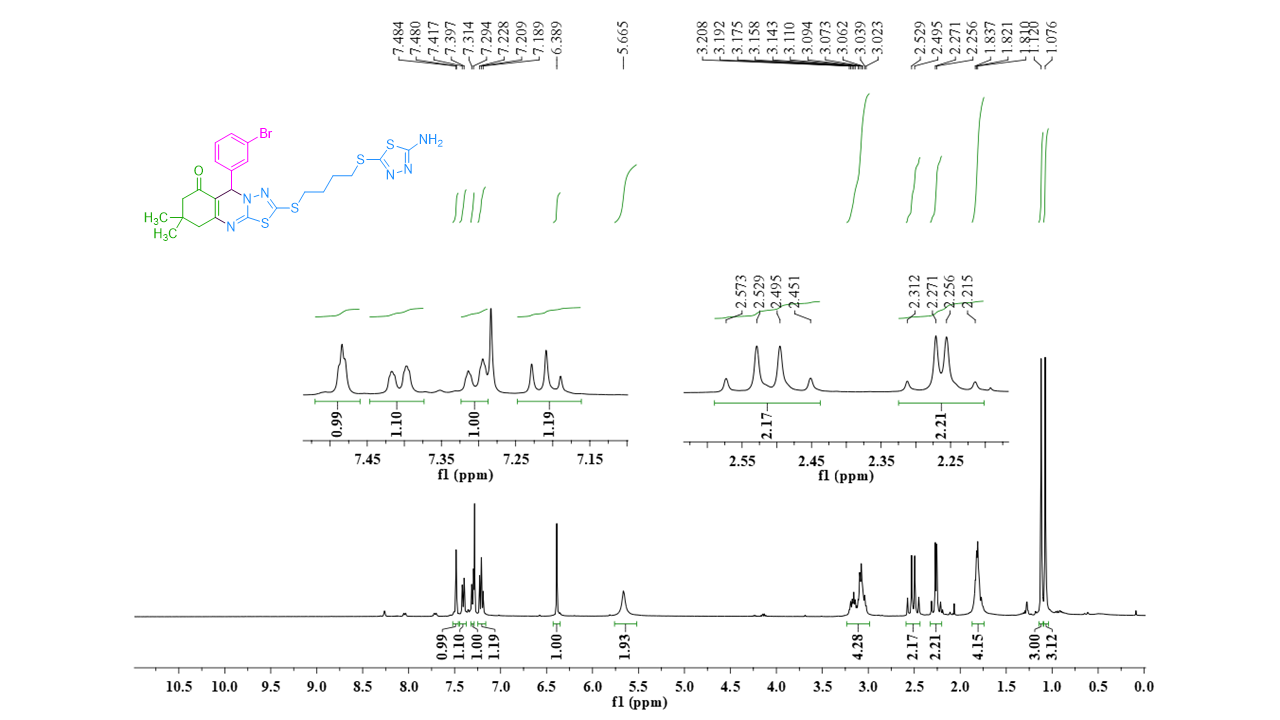
**

**Figure S74. ^1^H NMR (400 MHz, CDCl_3_) of 11b**

**
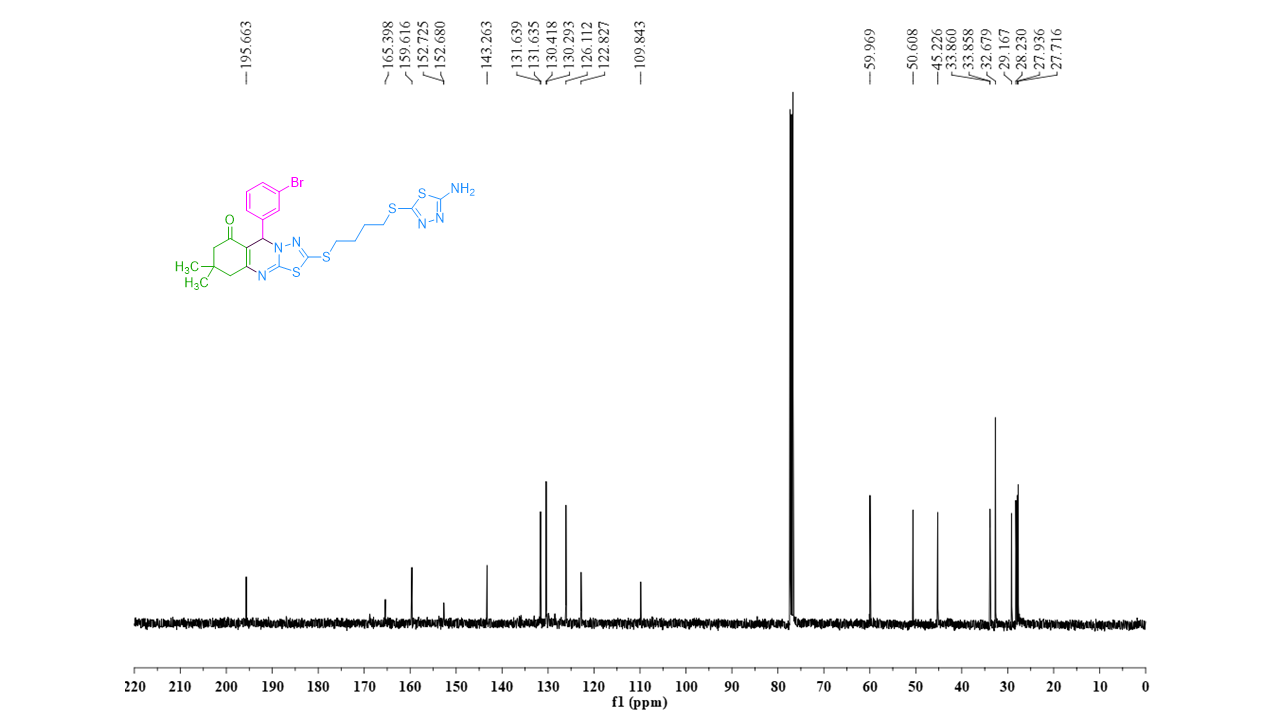
**

**Figure S75. ^13^C NMR (100 MHz, CDCl_3_) of 11b**

**
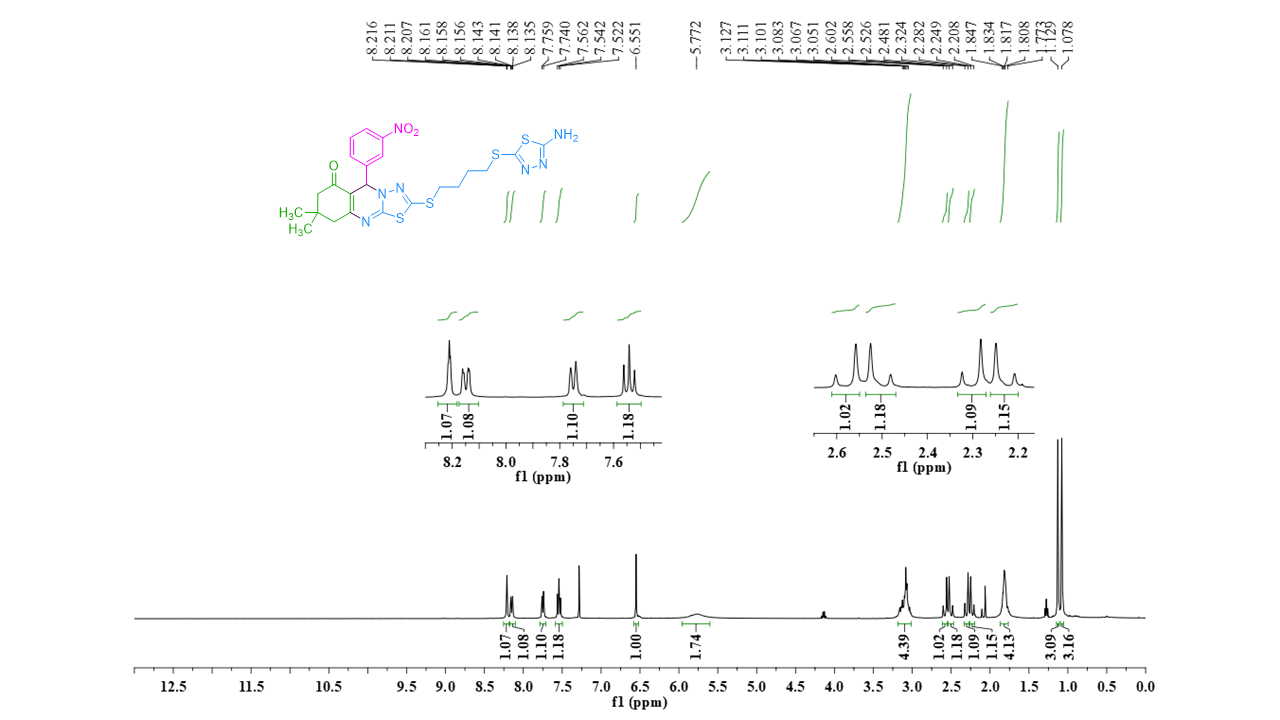
**

**Figure S76. ^1^H NMR (400 MHz, CDCl_3_) of 11c**

**
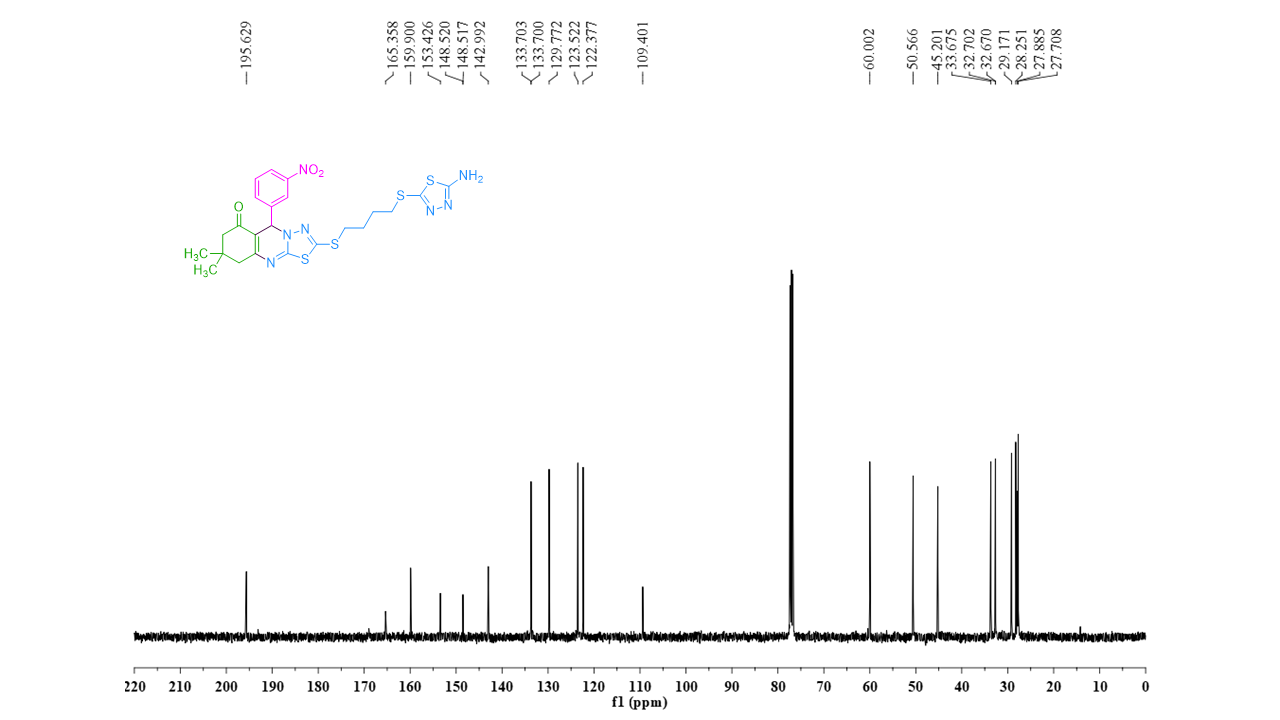
**

**Figure S77. ^13^C NMR (100 MHz, CDCl_3_) of 11c**

**
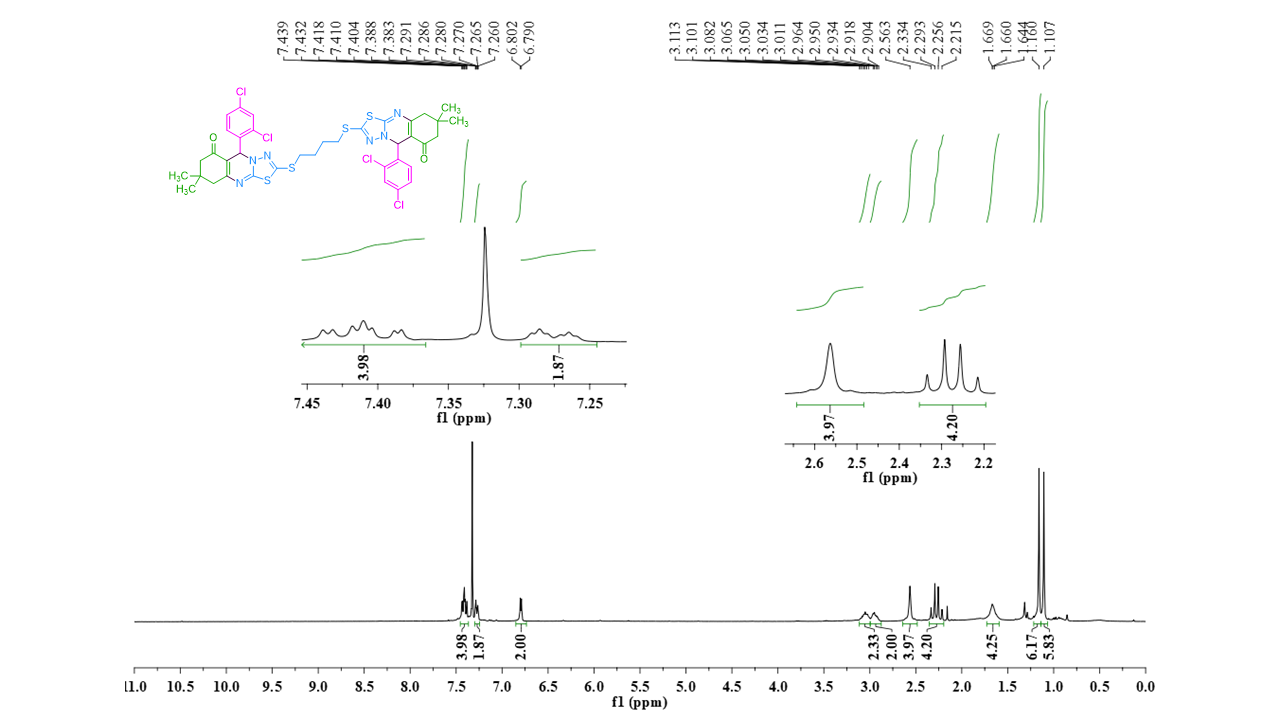
**

**Figure S78. ^1^H NMR (400 MHz, CDCl_3_) of 12a**

**
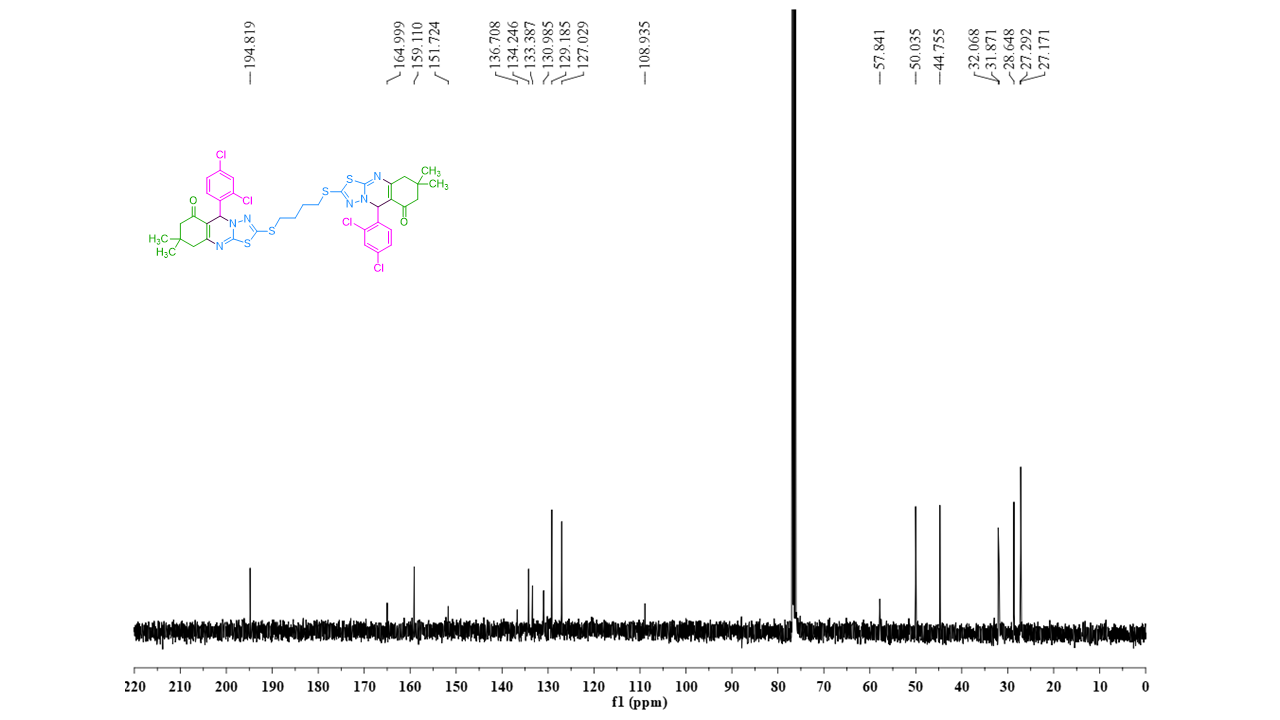
**

**Figure S79. ^13^C NMR (100 MHz, CDCl_3_) of 12a**

**
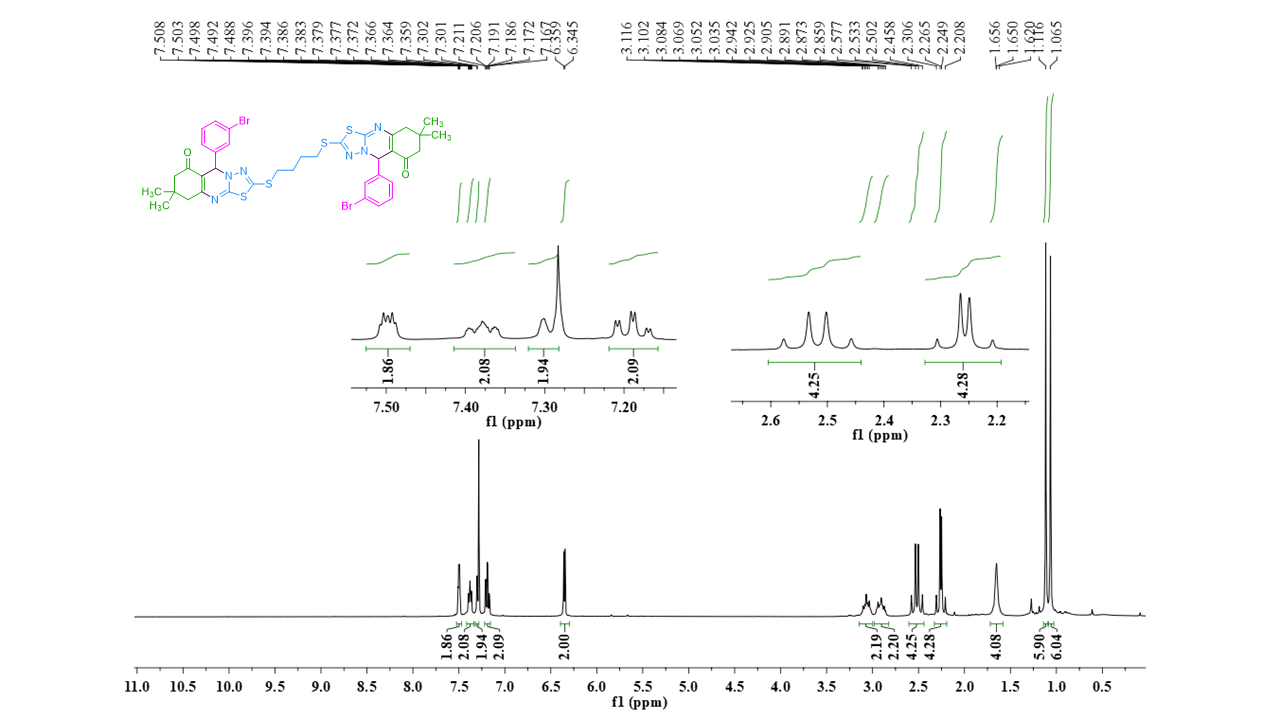
**

**Figure S80. ^1^H NMR (400 MHz, CDCl_3_) of 12b**

**
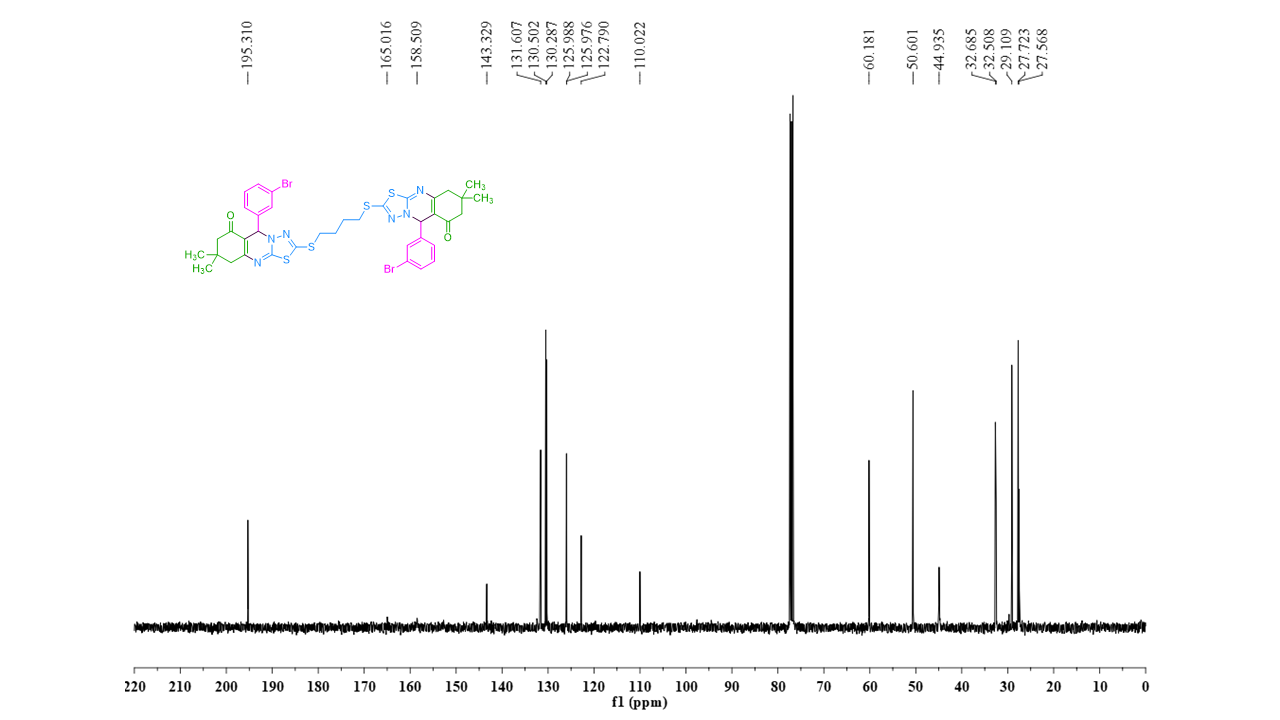
**

**Figure S81. ^13^C NMR (100 MHz, CDCl_3_) of 12b**

**
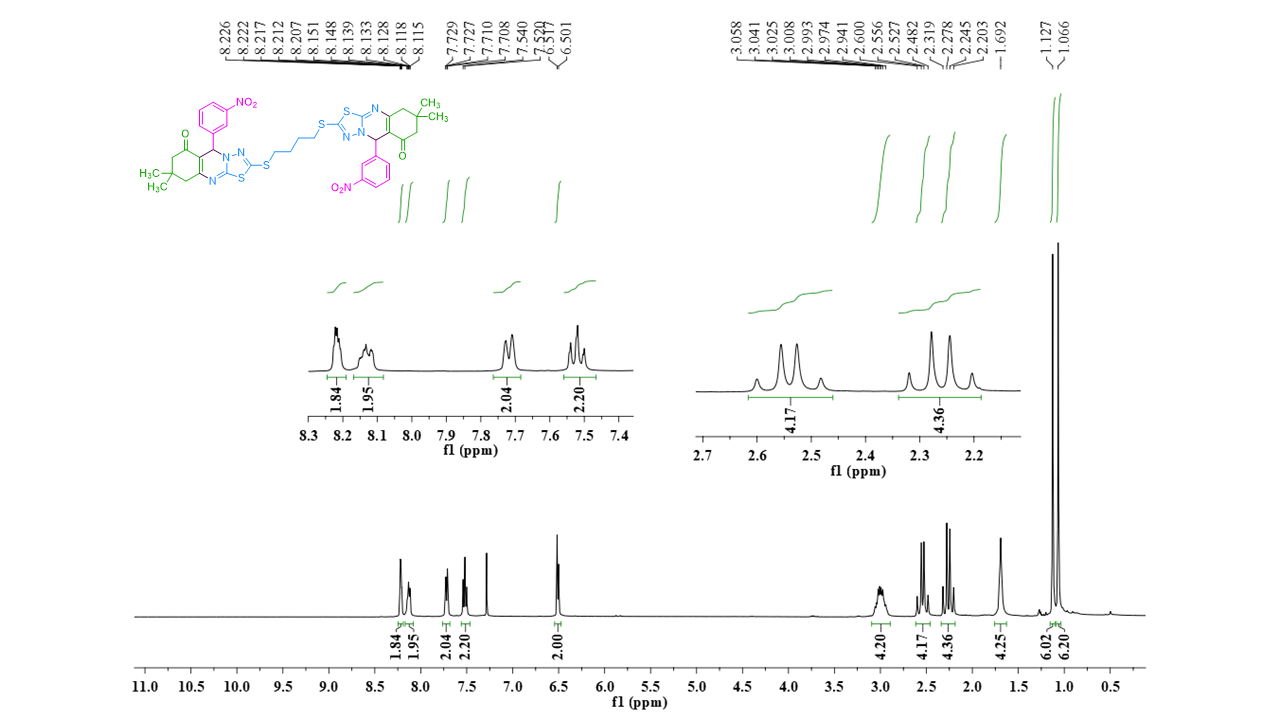
**

**Figure S82. ^1^H NMR (400 MHz, CDCl_3_) of 12c**

**
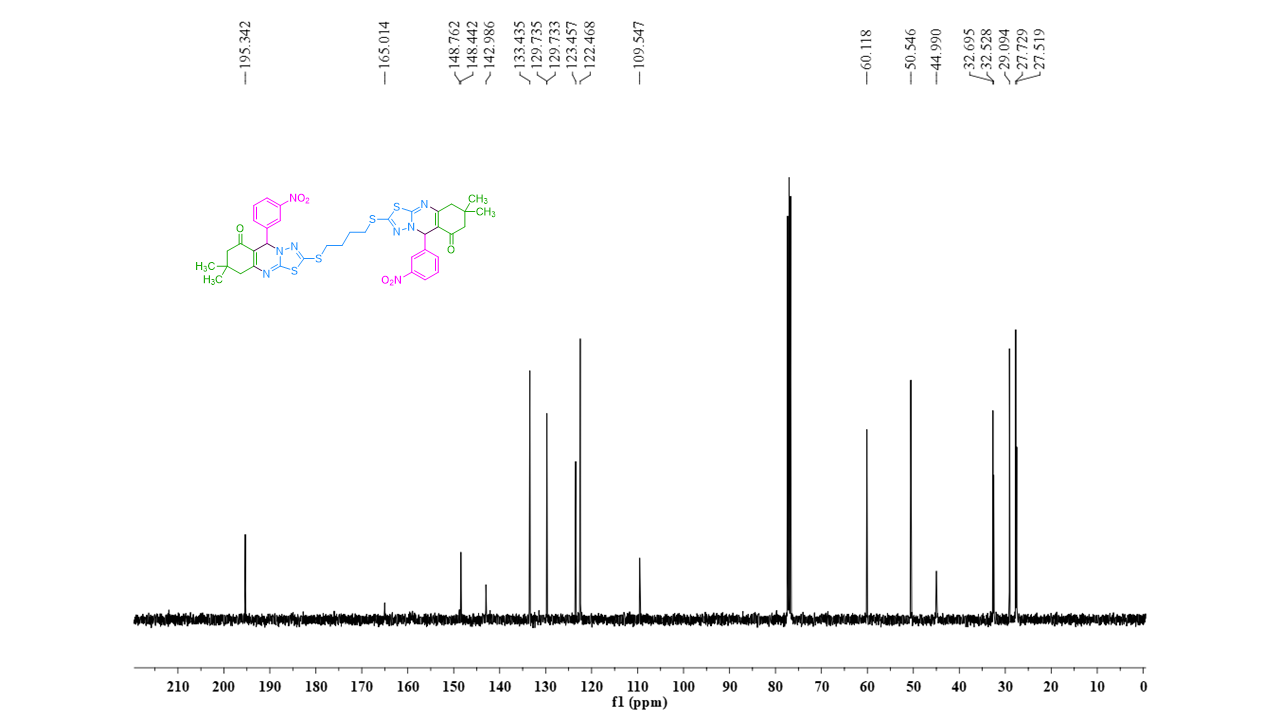
**

**Figure S83. ^13^C NMR (100 MHz, CDCl_3_) of 12c**


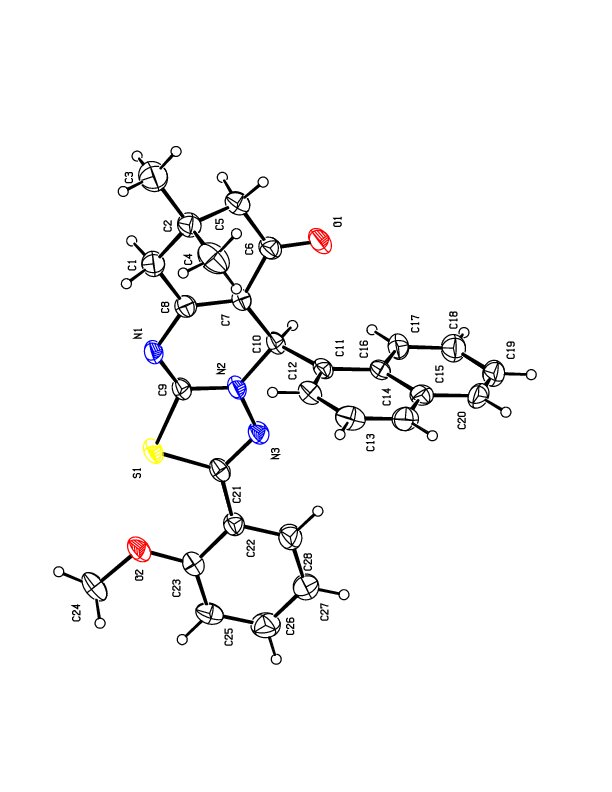


**Figure S84.** X-ray crystallography structure of 4s.

| Empirical formula | C_28_H_25_N_3_O_2_S |
| --- | --- |
| Formula weight | 467.57 |
| Temperature | 298(2) K |
| Wavelength | 0.71073 Å |
| Crystal system | Monoclinic |
| Space group | P 21/n |
| Unit cell dimensions | a = 12.641(3) Å alpha = 90 deg. |
|  | b = 11.782(2) Å beta = 101.43(3) deg. |
|  | c = 16.121(3) Å gamma = 90 deg. |
| Volume | 2353.4(9) Å^3^ |
| Z | 4 |
| Density (calculated) | 1.320 Mg/m^3^ |
| Absorption coefficient | 0.169 mm^-1^ |
| F(000)  Crystal size | 984.0  0.50 x 0.50 x 0.20 mm |
| Theta range for data collection | 1.88 to 25.00 deg. |
| Limiting indices | -15<=h<=15, -14<=k<=12, -19<=l<=16 |
| Reflections collected / unique | 10937 / 4119 [R(int) = 0.1127] |
| Completeness to theta | 25.00 99.5 % |
| Absorption correction | Numerical |
| Refinement method | Full-matrix least-squares on *F^2^* |
| Data / restraints / parameters | 4119 / 0 / 310 |
| Goodness-of-fit on *F*^2^ | 0.908 |
| Final R indices [I>2σ (I)] | R1 = 0.0609, wR2 = 0.0929 |
| R indices (all data) | R1 = 0.1457, wR2 = 0.1092 |
| Largest diff. peak and hole | 0.176 and -0.238 e.Å^-3^ |
| **Table S1.** Crystal data and structure refinement for **4s** | |

| S(1)-C(9)  S(1)-C(21)  O(1)-C(6)  O(2)-C(23)  O(2)-C(24)  N(1)-C(9)  N(1)-C(8)  N(2)-C(9)  N(2)-N(3)  N(2)-C(10)  N(3)-C(21)  C(1)-C(8)  C(1)-C(2)  C(1)-H(1A)  C(1)-H(1B)  C(2)-C(5)  C(2)-C(3)  C(2)-C(4)  C(3)-H(3A)  C(3)-H(3B)  C(3)-H(3C)  C(4)-H(4A)  C(4)-H(4B)  C(4)-H(4C)  C(5)-C(6)  C(5)-H(5A)  C(5)-H(5B)  C(6)-C(7)  C(7)-C(8)  C(7)-C(10)  C(10)-C(11)  C(10)-H(10)  C(11)-C(12)  C(11)-C(16)  C(12)-C(13)  C(12)-H(12)  C(13)-C(14)  C(13)-H(13)  C(14)-C(15)  C(14)-H(14)  C(15)-C(20)  C(15)-C(16)  C(16)-C(17)  C(17)-C(18)  C(17)-H(17)  C(18)-C(19)  C(18)-H(18)  C(19)-C(20)  C(19)-H(19)  C(20)-H(20)  C(21)-C(22)  C(22)-C(28)  C(22)-C(23)  C(23)-C(25)  C(24)-H(24A)  C(24)-H(24B)  C(24)-H(24C)  C(25)-C(26)  C(25)-H(25)  C(26)-C(27)  C(26)-H(26)  C(27)-C(28)  C(27)-H(27)  C(28)-H(28)  C(9)-S(1)-C(21)  C(23)-O(2)-C(24)  C(9)-N(1)-C(8)  C(9)-N(2)-N(3)  C(9)-N(2)-C(10)  N(3)-N(2)-C(10)  C(21)-N(3)-N(2)  C(8)-C(1)-C(2)  C(8)-C(1)-H(1A)  C(2)-C(1)-H(1A)  C(8)-C(1)-H(1B)  C(2)-C(1)-H(1B)  H(1A)-C(1)-H(1B)  C(5)-C(2)-C(3)  C(5)-C(2)-C(1)  C(3)-C(2)-C(1)  C(5)-C(2)-C(4)  C(3)-C(2)-C(4)  C(1)-C(2)-C(4)  C(2)-C(3)-H(3A)  C(2)-C(3)-H(3B)  H(3A)-C(3)-H(3B)  C(2)-C(3)-H(3C)  H(3A)-C(3)-H(3C)  H(3B)-C(3)-H(3C)  C(2)-C(4)-H(4A)  C(2)-C(4)-H(4B)  H(4A)-C(4)-H(4B)  C(2)-C(4)-H(4C)  H(4A)-C(4)-H(4C)  H(4B)-C(4)-H(4C)  C(6)-C(5)-C(2)  C(6)-C(5)-H(5A)  C(2)-C(5)-H(5A)  C(6)-C(5)-H(5B)  C(2)-C(5)-H(5B)  H(5A)-C(5)-H(5B)  O(1)-C(6)-C(7)  O(1)-C(6)-C(5)  C(7)-C(6)-C(5)  C(8)-C(7)-C(6)  C(8)-C(7)-C(10)  C(6)-C(7)-C(10)  C(7)-C(8)-N(1)  C(7)-C(8)-C(1)  N(1)-C(8)-C(1)  N(1)-C(9)-N(2)  N(1)-C(9)-S(1)  N(2)-C(9)-S(1)  N(2)-C(10)-C(7)  N(2)-C(10)-C(11)  C(7)-C(10)-C(11)  N(2)-C(10)-H(10)  C(7)-C(10)-H(10)  C(11)-C(10)-H(10)  C(12)-C(11)-C(16)  C(12)-C(11)-C(10)  C(16)-C(11)-C(10)  C(11)-C(12)-C(13)  C(11)-C(12)-H(12)  C(13)-C(12)-H(12)  C(14)-C(13)-C(12)  C(14)-C(13)-H(13)  C(12)-C(13)-H(13)  C(13)-C(14)-C(15)  C(13)-C(14)-H(14)  C(15)-C(14)-H(14)  C(14)-C(15)-C(20)  C(14)-C(15)-C(16)  C(20)-C(15)-C(16)  C(17)-C(16)-C(15)  C(17)-C(16)-C(11)  C(15)-C(16)-C(11)  C(18)-C(17)-C(16)  C(18)-C(17)-H(17)  C(16)-C(17)-H(17)  C(17)-C(18)-C(19)  C(17)-C(18)-H(18)  C(19)-C(18)-H(18)  C(20)-C(19)-C(18)  C(20)-C(19)-H(19)  C(18)-C(19)-H(19)  C(19)-C(20)-C(15)  C(19)-C(20)-H(20)  C(15)-C(20)-H(20)  N(3)-C(21)-C(22)  N(3)-C(21)-S(1)  C(22)-C(21)-S(1)  C(28)-C(22)-C(23)  C(28)-C(22)-C(21)  C(23)-C(22)-C(21)  O(2)-C(23)-C(25)  O(2)-C(23)-C(22)  C(25)-C(23)-C(22)  O(2)-C(24)-H(24A)  O(2)-C(24)-H(24B)  H(24A)-C(24)-H(24B)  O(2)-C(24)-H(24C)  H(24A)-C(24)-H(24C)  H(24B)-C(24)-H(24C)  C(23)-C(25)-C(26)  C(23)-C(25)-H(25)  C(26)-C(25)-H(25)  C(27)-C(26)-C(25)  C(27)-C(26)-H(26)  C(25)-C(26)-H(26)  C(26)-C(27)-C(28)  C(26)-C(27)-H(27)  C(28)-C(27)-H(27)  C(27)-C(28)-C(22)  C(27)-C(28)-H(28)  C(22)-C(28)-H(28) | 1.741(3)  1.743(4)  1.228(4)  1.357(4)  1.438(3)  1.296(4)  1.387(4)  1.346(4)  1.368(4)  1.464(4)  1.291(4)  1.485(5)  1.535(4)  0.9700  0.9700  1.516(5)  1.524(6)  1.539(6)  0.9600  0.9600  0.9600  0.9600  0.9600  0.9600  1.492(5)  0.9700  0.9700  1.448(4)  1.360(4)  1.501(4)  1.522(5)  0.9800  1.363(5)  1.424(5)  1.389(5)  0.9300  1.362(6)  0.9300  1.399(5)  0.9300  1.411(5)  1.415(5)  1.409(5)  1.358(5)  0.9300  1.392(5)  0.9300  1.347(6)  0.9300  0.9300  1.467(5)  1.382(5)  1.399(4)  1.369(5)  0.9600  0.9600  0.9600  1.372(5)  0.9300  1.370(5)  0.9300  1.370(5)  0.9300  0.9300  89.34(16)  118.9(3)  113.9(3)  117.8(3)  123.9(3)  118.0(3)  110.6(3)  114.7(3)  108.6  108.6  108.6  108.6  107.6  111.3(4)  107.4(3)  109.7(3)  109.1(3)  109.3(4)  110.0(3)  109.5  109.5  109.5  109.5  109.5  109.5  109.5  109.5  109.5  109.5  109.5  109.5  114.7(3)  108.6  108.6  108.6  108.6  107.6  120.1(4)  121.1(3)  118.7(3)  119.9(3)  123.3(3)  116.7(3)  124.0(3)  122.2(3)  113.7(3)  127.8(3)  124.3(2)  108.0(3)  106.9(2)  109.9(3)  113.5(3)  108.8  108.8  108.8  118.9(3)  118.9(3)  122.2(3)  122.3(4)  118.9  118.9  119.5(4)  120.3  120.3  121.2(4)  119.4  119.4  122.0(4)  119.0(4)  118.9(4)  117.4(3)  123.5(3)  119.1(4)  122.2(4)  118.9  118.9  119.8(4)  120.1  120.1  120.2(4)  119.9  119.9  121.5(4)  119.2  119.2  120.5(3)  114.3(3)  125.2(2)  118.1(4)  118.7(3)  123.2(3)  124.5(3)  115.4(3)  120.1(4)  109.5  109.5  109.5  109.5  109.5  109.5  120.7(3)  119.7  119.7  120.0(4)  120.0  120.0  119.8(4)  120.1  120.1  121.3(3)  119.4  119.4 |
| --- | --- |
| Table S2. Bond lengths [A] and angles [deg] for 4s | |

**Validation of green chemistry metrics for all synthesized compounds.**

The following formulae were used for the calculation of Effective Mass Yield (EMY), Atom Economy (AE), Atom Efficiency (AEf), Carbon Efficiency (CE), Reaction Mass Efficiency (RME), Optimum Efficiency (OE), and E-factor.^2-6^ Calculated data for all synthesized compounds are presented in Table S1.
**Materials used for metrics calculations:**

**For 4(a-v), 6 (a-d), 8(a-d), 9 (a-d), 11 (a-c), and 12 (a-c) series of compounds:**

Dimedone/1,3-cyclohexanedione (**1**; 1 mmol), aldehyde (**2**; 1 mmol), 5-aryl-1,3,4-thiadiazol-2-amine (**3**; 1 mmol), [HDPH]: CoCl_4_^2-^ (0.2mmol) under solvent-free conditions.

**Note** **1:** For the synthesis of **6a-d** 3-(5-amino-1,3,4-thiadiazol-2-yl)-2H-chromen-2-one have been used.

**Note 2:** For the synthesis of **9a-d** 0.5 mmol of mmol dialdehyde has been used.

**Note 3:** For the synthesis of **11a-c** 1 mmol of 5,5'-((butane-1,4-diylbis(sulfanediyl))bis(4,1-phenylene))bis(1,3,4-thiadiazol-2-amine) have been used.

**Note 4:** For the synthesis of **12a-c** 0.5 mmol of 5,5'-((butane-1,4-diylbis(sulfanediyl))bis(4,1-phenylene))bis(1,3,4-thiadiazol-2-amine) have been used.

**Respective amounts:**

**Reagents (Molecular weight, MW):** dimedone: 0.280 or 0.140 g (MW: 140.18), 1,3-cyclohexanedione: 0.224 or 0.112 g (MW: 112.13), 2-chlorobenzaldehyde: 0.140 g (MW: 140.57), 4-chlorobenzaldehyde: 0.140 g (MW: 140.57), 2,4-dichlorobenzaldehyde: 0.175 g (MW: 175.01), 4-bromobenzaldehyde: 0.185 g (MW: 185.02), 3-bromobenzaldehyde: 0.185 g (MW: 185.02), 3-methoxybenzaldehyde : 0.272 g (MW: 136.15), 4-methylbenzaldehyde : 0.120 g (MW: 120.15), 4-nitrobenzaldehyde : 0.151 g (MW: 151.12), 3-nitrobenzaldehyde : 0.151 g (MW: 151.12), benzaldehyde: 0.212 g (MW: 106.12), 2,4-dimethoxybenzaldehyde: 0.166 g (MW: 166.17), 2-hydroxybenzaldehyde: 0.122 g (MW: 122.12), naphthalene-1-carbaldehyde: 0.156 g (MW: 156.18), 3,5-dimethoxy-4-hydroxybenzaldehyde: 0.182 g (MW: 182.18), 4-(benzyloxy)benzaldehyde: 0.212 g (MW: 212.25), 3-phenylpropinal: 0.134 g (MW: 134.18), [1,1'-biphenyl]-4-carbaldehyde: 0.182 g ( MW: 182.22), 4-(dimethylamino)benzaldehyde: 0.149 g (MW: 149.19), 6-chloro-4-oxo-4H-chromene-3-carbaldehyde: 0.208 g (MW: 208.60), 4-(tert-butyl)benzaldehyde: 0.162 g (MW: 162.23), terephthalaldehyde: 0.134 g (MW: 134.13), isophthalaldehyde: 0.134 g (MW: 134.13), 5-phenyl-1,3,4-thiadiazol-2-amine: 0.354 or 0.177 g (MW: 177.22), 5-(o-tolyl)-1,3,4-thiadiazol-2-amine: 0.382 or 0.191 g ( MW: 191.25), 5-(2-methoxyphenyl)-1,3,4-thiadiazol-2-amine: 0.414 or 0.207 g (MW: 207.25), 5-(4-nitrophenyl)-1,3,4-thiadiazol-2-amine: 0.222 g (MW: 222.22), 3-(5-amino-1,3,4-thiadiazol-2-yl)-2H-chromen-2-one: 0.245 g (MW: 245.26), 5,5'-((butane-1,4-diylbis(sulfanediyl))bis(4,1-phenylene))bis(1,3,4-thiadiazol-2-amine): 0.320 or 0.160 g (MW: 320.47)

**Solvents:**

This reaction carry out under solvent-free conditions.

**Products (Molecular weight, MW):**

**Products 4(a-v):
4a:** 0.361 g (MW: 401.53), **4b:** 0.394 g (MW: 432.50), **4c:** 0.406 g (MW: 466.40), **4d:** 0.439 g (MW: 477.58), **4e:** 0.424 g (MW: 446.53), **4f:** 0.405 g (MW: 435.97), **4g:** 0.487 g (MW: 507.65), **4h:** 0.385 g (MW: 442.36), **4i:** 0.364 g (MW: 433.53), **4j:** 0.416 g (MW:462.52), **4k:** 0.452 g (MW: 486.41), **4l:** 405 g (MW: 449.53), **4m:** 431 g (MW: 468.37), **4n:** 0.399 g (MW: 511.39), **4o:** 0.318 g (MW: 435.50), **4p:** 0.354 g (MW: 447.51), **4q:** 0.371 g (MW: 463.60), **4r:** 0.376 g (MW: 453.55), **4s:** 426 g ( MW: 467.59), **4t:** 0.416 g (MW: 489.97), **4u:** 0.333 g (MW: 387.50),  **4v:** 0.345 g (MW: 460.55).

**Products 6(a-d):**

**6a:** 0.348 g (MW: 489.97), **6b:** 0.388 g (MW: 485.56), **6c:** 0.397 g (MW: 472.48), **6d:** 0.402 g (MW: 485.56).

**Products 8(a-d):
8a:** 0.291 g (MW: 415.51), **8b:** 0.334 g (MW: 445.54), **8c:** 0.396 g (MW: 609.74), **8d:** 0.293 g (MW: 401.48).

**Products 9(a-d):
9a:** 0.432 g (MW: 696.89), **9b:** 0.492 g (MW: 756.94), **9c:** 0.553 g (MW: 921.14), **9d:** 0.428 g (MW: 668.83).

**Products 11(a-c):
11a:** 0.360 g (MW: 599.63), **11b:** 0.433 g (MW: 609.64), **11c:** 0.438 g (MW: 575.74).

**Products 12(a-c):
12a:** 0.501 g (MW: 878.79), **12b:** 0.611 g (MW: 898.81), **12c:** 0.582 g (MW: 831.01).

**Calculation of green chemistry metrics for one representative entry, viz. 4a.**

$$EMY =\frac{Mass of isolated product}{Mass of non - benign reagents}\times1\boldsymbol{00}$$

- $\boldsymbol{EMY}\mathbf{=}\frac{\mathbf{0.361}}{\mathbf{0.140+0}\boldsymbol{.120+0.177}}\boldsymbol{\times100=82.61}\boldsymbol{\%}$

$$AE =\frac{\mathrm{Molecular} \mathrm{Weight} \mathrm{of} \mathrm{Produc}s}{\mathrm{Total} \mathrm{Molecular} \mathrm{Weight} \mathrm{of} \mathrm{Reactant}s}\times1\boldsymbol{00}$$

- $\boldsymbol{AE}\mathbf{=}\frac{\mathbf{4}\boldsymbol{01.53}}{\mathbf{140.18+120.15+177.22}}\boldsymbol{\times100}\mathbf{=91.77}\boldsymbol{\%}$

$$AEf=AE\times Yield \%$$

- $\boldsymbol{AEf=91.77\times90\%=82.59 \%}$

$$CE=\frac{Amount of carbon in the product}{Total carbon in reactants}\times100$$

- $\boldsymbol{CE}\mathbf{=}\frac{\boldsymbol{24\times0}\boldsymbol{.0009}}{\boldsymbol{8\times0.001+8\times0.001+8}\boldsymbol{\times0.001}}\boldsymbol{\times100=90\%}$

$$RME =\frac{Mass of isolated product}{Total mass of reactant}\times1\boldsymbol{00}$$

- $\boldsymbol{RME}\mathbf{=}\frac{\mathbf{0.361}}{\mathbf{0.140+0}\boldsymbol{.120+0.177}}\boldsymbol{\times100=82.61 \%}$

$$OE =\frac{\mathrm{RME}}{\mathrm{AE}}\times1\boldsymbol{00}$$

- $\boldsymbol{OE}\mathbf{=}\frac{\mathbf{82.61}}{\mathbf{91.77}}\boldsymbol{\times100}\boldsymbol{=90.02\%}$

$$E-factor=\frac{Mass of raw materials - Mass of product}{Mass of product}$$

- $\boldsymbol{E-factor}\mathbf{=}\frac{\mathbf{0.140+0.120+0.177-0.361}}{\mathbf{0.361}}\boldsymbol{=0.21 g/g}$

| E-factor  (g/g) | OE  (%) | RME  (%) | CE  (%) | AEf  (%) | AE  (%) | EMY  (%) | Yield  (%) | Product | Sr. No. |
| --- | --- | --- | --- | --- | --- | --- | --- | --- | --- |
| 0.21 | 90.02 | 82.61 | 90 | 82.59 | 91.77 | 82.61 | 90 | 4a | 1 |
| 0.19 | 91.20 | 84.19 | 91 | 84.00 | 92.31 | 84.19 | 91 | 4b | 2 |
| 0.24 | 87.13 | 80.88 | 87 | 80.76 | 92.83 | 80.88 | 87 | 4c | 3 |
| 0.17 | 92.03 | 85.58 | 92 | 85.55 | 92.99 | 85.58 | 92 | 4d | 4 |
| 0.14 | 95.06 | 87.97 | 95 | 87.91 | 92.54 | 87.97 | 95 | 4e | 5 |
| 0.16 | 93.09 | 85.99 | 93 | 85.90 | 92.37 | 85.99 | 93 | 4f | 6 |
| 0.11 | 96.06 | 89.69 | 96 | 89.64 | 93.37 | 89.69 | 96 | 4g | 7 |
| 0.24 | 86.87 | 80.33 | 87 | 80.45 | 92.47 | 80.33 | 87 | 4h | 8 |
| 0.29 | 84.06 | 77.61 | 84 | 77.56 | 92.33 | 77.61 | 84 | 4i | 9 |
| 0.20 | 90.04 | 83.53 | 90 | 83.49 | 92.77 | 83.53 | 90 | 4j | 10 |
| 0.15 | 93.01 | 86.59 | 93 | 86.58 | 93.10 | 86.59 | 93 | 4k | 11 |
| 0.20 | 90.20 | 83.51 | 90 | 83.32 | 92.58 | 83.51 | 90 | 4l | 12 |
| 0.17 | 92.10 | 85.52 | 92 | 85.43 | 92.86 | 85.52 | 92 | 4m | 13 |
| 0.37 | 78.09 | 72.94 | 78 | 72.86 | 93.41 | 72.94 | 78 | 4n | 14 |
| 0.47 | 73.10 | 67.95 | 73 | 67.85 | 92.95 | 67.95 | 73 | 4o | 15 |
| 0.36 | 79.19 | 73.29 | 79 | 73.11 | 92.55 | 73.29 | 79 | 4p | 16 |
| 0.35 | 80.13 | 74.35 | 80 | 74.23 | 92.79 | 74.35 | 80 | 4q | 17 |
| 0.25 | 83.00 | 79.83 | 83 | 79.83 | 96.18 | 79.83 | 83 | 4r | 18 |
| 0.18 | 91.21 | 84.69 | 91 | 84.49 | 92.85 | 84.69 | 91 | 4s | 19 |
| 0.26 | 85.07 | 79.24 | 85 | 79.18 | 93.15 | 79.24 | 85 | 4t | 20 |
| 0.27 | 86.04 | 78.72 | 86 | 78.68 | 91.49 | 78.72 | 86 | 4u | 21 |
| 0.44 | 75.00 | 69.56 | 75 | 69.56 | 92.74 | 69.56 | 75 | 4v | 22 |
| 0.51 | 71.16 | 66.29 | 71 | 66.14 | 93.15 | 66.29 | 71 | 6a | 23 |
| 0.34 | 80.00 | 74.47 | 80 | 74.47 | 93.09 | 74.47 | 80 | 6b | 24 |
| 0.31 | 82.19 | 76.49 | 82 | 76.32 | 93.07 | 76.49 | 82 | 6c | 25 |
| 0.26 | 85.17 | 79.13 | 85 | 78.97 | 92.91 | 79.13 | 85 | 6d | 26 |
| 0.55 | 70.12 | 64.52 | 70 | 64.41 | 92.02 | 64.52 | 70 | 8a | 27 |
| 0.44 | 75.05 | 69.44 | 75 | 69.39 | 92.52 | 69.44 | 75 | 8b | 28 |
| 0.63 | 65.03 | 61.40 | 65 | 61.37 | 94.42 | 61.40 | 65 | 8c | 29 |
| 0.49 | 73.07 | 67.05 | 73 | 66.98 | 91.76 | 67.05 | 73 | 8d | 30 |
| 0.78 | 62.07 | 56.25 | 62 | 56.19 | 90.63 | 56.25 | 62 | 9a | 31 |
| 0.68 | 65.08 | 59.42 | 65 | 59.35 | 91.31 | 59.42 | 65 | 9b | 32 |
| 0.79 | 60.11 | 55.75 | 60 | 55.65 | 92.75 | 55.75 | 60 | 9c | 33 |
| 0.73 | 64.07 | 57.84 | 64 | 57.77 | 90.27 | 57.84 | 64 | 9d | 34 |
| 0.76 | 60.10 | 56.69 | 60 | 56.60 | 94.33 | 56.69 | 60 | 11a | 35 |
| 0.49 | 71.10 | 67.13 | 71 | 67.04 | 94.42 | 67.13 | 71 | 11b | 36 |
| 0.39 | 76.18 | 71.69 | 76 | 71.52 | 94.11 | 71.69 | 76 | 11c | 37 |
| 0.90 | 57.07 | 52.74 | 57 | 52.68 | 92.42 | 52.74 | 57 | 12a | 38 |
| 0.59 | 68.04 | 62.99 | 68 | 62.95 | 92.58 | 62.99 | 68 | 12b | 39 |
| 0.55 | 70.12 | 64.52 | 70 | 64.41 | 92.02 | 64.52 | 70 | 12c | 40 |
| **Table S3.** Calculated green metrics (EMY, AE, AEf, CE, RME, OE and E-factor) for the synthesized compounds (**4a**-**12c**) | | | | | | | | | |

**References**

1. Khansole, G. S.; Prasad, D.; Angulwar, J. A.; Atar, A. B.; Nagaraja, B. M.; Jadhav, A. H.; Bhosale, V. N., Tetrabutylammonium hydrogen sulfate mediated three-component reaction for the synthesis of thiadiazolo [2,3-b] quinazolin-6-(7*H*)-ones and antioxidant activity. *Mater*. *Today: Proc*. **9**, 653-660, DOI: https://doi.org/10.1016/j.matpr.2018.10.389 (2019).

2. Abou-Shehada, S.; Mampuys, P.; Maes, B.; Clark, J.; Summerton, L., An evaluation of credentials of a multicomponent reaction for the synthesis of isothioureas through the use of a holistic CHEM21 green metrics toolkit. *Green Chem*. **19**, 249-258, DOI: https://doi.org/ 10.1039/C6GC01928E (2017).

3. Brahmachari, G.; Nayek, N.; Karmakar, I.; Nurjamal, K.; Chandra, S. K.; Bhowmick, A., Series of functionalized 5-(2-arylimidazo [1,2-a] pyridin-3-yl) pyrimidine-2,4 (1*H*, 3*H*)-diones: a water-mediated three-component catalyst-free protocol revisited. *J*. *Org*. *Chem*. **85**, 8405-8414, DOI: https://doi.org/10.1021/acs.joc.0c00732 (2020).

4. Chen, Y.-X.; Zhang, M.; Zhang, S.-Z.; Hao, Z.-Q.; Zhang, Z.-H., Copper-decorated covalent organic framework as a heterogeneous photocatalyst for phosphorylation of terminal alkynes. *Green Chem*. **24**, 4071-4081, DOI: https://doi.org/10.1039/d2gc00754a (2022).

5. Moeini Korbekandi, M.; Mohammadpoor-Baltork, I.; Moghadam, M.; Tangestaninejad, S.; Mirkhani, V.; Omidvar, A.; Notash, B., Diphenhydramine hydrochloride–CuCl as a new catalyst for the synthesis of tetrahydrocinnolin-5 (1*H*)-ones. *ACS omega*, **8**, 15883-15895 DOI: https://doi.org/10.1021/acsomega.2c06765 (2023).

6. McElroy, C. R.; Constantinou, A.; Jones, L. C.; Summerton, L.; Clark, J. H., Towards a holistic approach to metrics for the 21st century pharmaceutical industry. *Green Chem*. **17**, 3111-3121, DOI: https://doi.org/10.1039/c5gc00340g (2015).
